# The Sentinel Sleep Theory: Unweaving the biological function of REM sleep

**DOI:** 10.1016/j.sleepx.2026.100186

**Published:** 2026-04-09

**Authors:** Raffael Brito Spinassi

**Affiliations:** Atibaia, São Paulo, Brazil

## Abstract

The biological function of Rapid Eye Movement (REM) sleep remains one of neuroscience’s great mysteries. In this theoretical research paper, I present the evolutionary theory that explains why REM sleep exists. I demonstrate that REM sleep functions to heighten brain alertness to significantly mitigate the high vulnerability inherent in non‑REM sleep—especially in deep sleep. Every organism with a nervous system must undergo non-REM sleep, a necessity that accompanies a negative and potentially lethal consequence: a higher risk of dying. Because non‑REM sleep substantially reduces alertness and increases the death risk, REM sleep evolved as an adaptive countermeasure, making it a *necessary adaptation* for any organism that must sleep. My theory—grounded in evolutionary biology and voluminous empirical evidence—provides an eclectic and far‑reaching explanatory and predictive capacity. This is because it integrates hundreds of pieces of evidence and generates numerous testable hypotheses that cross multiple scientific fields, such as genetics, phylogenetics, embryology, physiology, endocrinology, and immunology. In this paper, I discuss **452 references**, most of which serve to support my theory. Based on the available evidence, *all empirically testable predictions that I was able to verify were corroborated*. Furthermore, the theory also resisted numerous other attempts at refutation. Here, I also challenge traditional views (e.g., REM sleep aids learning and memory), arguing that these apparent functions are byproducts rather than primary evolutionary drivers. Thus, this basic research may contribute to advancing theoretical neuroscience and the planning and execution of future REM sleep research.

## Highlights

• REM sleep's primary function is to reduce the vulnerability caused by N-REM sleep.
• REM sleep parameters are directly related to the body's protection or vulnerability.
• REM sleep is cyclic due to its protective function.
• REM sleep probably emerged as a brief awakening from N-REM sleep.
• REM density is a measure of the organism's alertness.

## Graphical abstract

**Figure:**
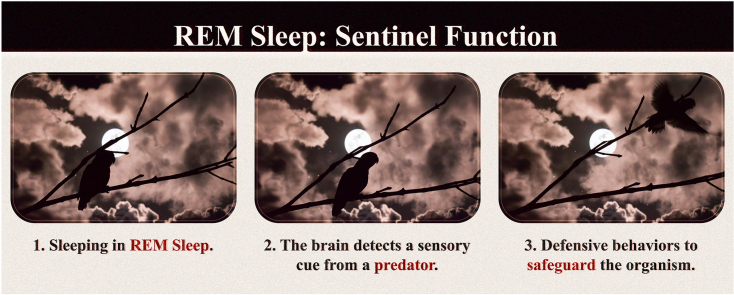

## 1 Introduction

Imagine yourself as a wild animal. The place where you live and sleep—nature—is often dangerous and lethal. Therefore, you must constantly struggle to preserve your life. It turns out that your brain, when sleeping, drastically reduces your attention to the environment and your ability to respond to any dangers. Sleep is necessary, but it greatly hinders your arduous task of staying alive. Whatever the function (or functions) of this deep and restorative sleep may be, its weaknesses—reduced attention, diminished motor responsiveness, and compromised safety—cannot be eliminated. If your only option were to remain at the mercy of this necessary but dangerous sleep, your chances of survival would be drastically shortened.

*But what if there were a neural mechanism capable of increasing your safety while you sleep?* Well, in that case, your chances of surviving despite the dangers of sleep would begin to look far more reasonable. Here, I propose that the neural state we call Rapid Eye Movement sleep (or REM sleep) is this mechanism. This is a simple—and effective—solution to a problem that could cost you your life.

But make no mistake. The simplicity of this mechanism does not translate into ease of unraveling its function. Many scientists (especially psychologists, neuroscientists, and physicians) have been trying to discover what REM sleep is for more than 70 years, ever since Eugene Aserinsky and Nathaniel Kleitman [1], in a landmark and foundational study, contributed to its discovery and reporting. An even earlier work by Maria Denisova and Nicholai Figurin, originally published in Russian in 1926, is the first study in which the authors presented data on what is now recognized as REM sleep. It is available in an English translation [2] and likewise establishes the authors as important figures in the discovery and description of REM sleep.

Why is it so difficult, at the moment, for scientists to describe what REM sleep is? The primary reason is that they still do not understand the biological function of this sleep state. The biological function of REM sleep remains an unsolved question and stands as one of the major enigmas of neuroscience—indeed, of science [[3], [4], [5], [6], [7]]. And when you do not know the function of a mechanism, you are limited to describing its physical and behavioral aspects. My objective in this theoretical work is to unweave the evolutionary reason for the existence of REM sleep. I will begin by summarizing some of its fundamental characteristics.

REM sleep is many things: a brain state, a behavior, a sleep state, a dreaming state, as well as a paradoxical state [8,9]. Scientists classify REM sleep as a sleep state because arousal thresholds increase in this state [10,11], causing the organism to stop responding behaviorally to the external environment in the same way it does during wakefulness [12]. Indeed, the arousal thresholds of mammals can be as high during REM sleep as they are during N-REM sleep [[10], [11], [12], [13], [14]].

During REM sleep, the sleeping organism (with an elevated arousal threshold) exhibits neural activity similar to that of wakefulness [8,12]. The physiology during REM sleep is so similar to wakefulness that the electroencephalogram (EEG) shows electrical activity almost indistinguishable from that occurring in the brain during wakefulness [4,12]. This is why REM sleep was originally termed paradoxical sleep [9].

Especially in mammals and birds, both the REM sleep period and the non-REM (or N-REM) sleep period are marked by specific and easily distinguishable physiological changes [15,16]. The physiological changes that occur during the REM period contrast with those of the N-REM period and exhibit comparatively *higher rates* [17]. Unsurprisingly, the REM period increases energy expenditure [18]. After all, metabolic activity, blood pressure, and respiratory and heart rates rise to levels that appear as if the organism were awake [[16], [17], [18]].

During REM sleep, brain metabolism increases by about 20% due to the higher intensity of neural activity, making it clear that the brain does not rest in this state [4,6]. Considering that the reverberation of neural patterns during sleep is energetically more costly than neuronal silencing [19] and that REM sleep causes a significant energy expenditure, this cost indicates that REM sleep plays a critical role. After all, non-random elimination (or natural selection) is highly effective at eliminating waste. Nothing so costly lasts for several million years unless it serves an important function—a frequently neglected evolutionary consequence [20].

Many scientists have tried to uncover the function of REM sleep, but their proposals were not unanimously accepted because they are incapable of explaining an abundance of disparate facts pertaining to the domain of REM sleep and are inconsistent with the evidence, or at least with parts of it. Here are some of the various hypotheses already proposed: *learning* [21]; *sentinel function* [22]; *psychological health* [23]; *reverse learning* [24]; *brain warming function* [25]; *energy regulation* [26]; *sensorimotor integration* [27]; and *defensive activation of the visual cortex* [28].

The article that Snyder published in 1966 is particularly relevant for my discussion. In it, the author presented the “sentinel hypothesis” to try to explain the function of REM sleep. Although this concept was later developed (e.g., Ref. [29]), the sentinel function of REM sleep remained a *hypothesis*. My goal in this article is to develop this hypothesis into a *theory* of the function of REM sleep. This highlights the disparity between my work and Snyder's.

My scientific contribution in this article is to present a comprehensive conceptual framework—supported by extensive empirical evidence—that will turn the sentinel *hypothesis* into the *theory* of sentinel sleep. This work is a theoretical article that aims to contribute to elucidating the *primary* function of REM sleep. To demonstrate the validity and robustness of my theory, I need to demonstrate that numerous hypotheses derived from it are true (i.e., factually verified). Therefore, I must present the theory in a way that allows it to be refuted or corroborated by testing the hypotheses derived from it.

## 2 Methodology

To develop and test my theory, I adopted the *hypothetico-deductive* (H-D) method—a classic scientific investigation procedure for constructing and justifying scientific theories [[30], [31], [32]]. This method is based on starting from a theory or hypothesis, formulated conjecturally and still lacking justification, and then deriving logically deduced conclusions. The next step is for the scientist to test these conclusions (or predictions) against empirical evidence [32,33]. The aim of this method is to determine the validity of the theory by testing specific predictions. If empirical evidence corroborates the deduced predictions, the theory gains support; if, on the other hand, empirical evidence contradicts those predictions, it also challenges (or falsifies) the theory [30,32,33].

To test a theory, we can employ the following procedures [32,34,35]: (1) logically compare its specific predictions, in order to verify whether the theory is internally consistent; (2) examine the logical structure of the theory, in order to verify whether it possesses an empirical, scientific character, or whether it is a tautology; (3) compare it with other theories or hypotheses, in order to verify whether the newly proposed theory can effectively advance scientific knowledge once it is rigorously tested; (4) derive specific predictions, in order to test them against empirical evidence; (5) verify that it convincingly solves significant empirical and conceptual problems; (6) ensure that it possesses great explanatory power, being able to unify disparate empirical evidence; (7) ensure that it postulates only what is necessary (i.e., it must be parsimonious, following Ockham's razor, but without compromising its explanatory power).

I followed these seven procedures to test the Sentinel Sleep Theory, ensuring its internal consistency, empirical character, scientific value, easily testable predictions, ability to satisfactorily solve significant empirical and conceptual problems, great explanatory power, and parsimony.

I chose the H-D method due to the following methodological strengths:

1. **Easy to test empirically.** This method allows the scientist to formulate hypotheses that generate specific predictions that can be corroborated or refuted by empirical evidence.
2. **Logical rigor.** The chain “theory ⇒ hypotheses ⇒ specific predictions ⇒ empirical testing” provides methodological rigor and allows us to critically evaluate predictions.
3. **Cumulative corroboration.** In the case of theories that resist successive attempts at refutation, they accumulate a greater number of corroborations, giving rise to new research and hypotheses that expand and refine the theoretical framework.
4. **Reproducibility.** Due to the specific predictions, other researchers can test the hypotheses to verify whether they obtain the same results, thus strengthening the reliability of the results obtained.
5. **Experimental orientation.** The H-D method encourages scientists to design controlled and reproducible experiments. This also helps strengthen the reliability of the results obtained.
6. **Interdisciplinary flexibility.** With appropriate caveats (see below), we can apply this method in different scientific fields.

However, it is evident that, like any method, the H-D approach entails its own limitations. Below, I list some of its main shortcomings:

1. **It is not immune to the sampling problem.** Although the core of the H-D method is deductive reasoning, it is not entirely devoid of induction (see below). This makes it necessary to address the challenges inherent in the representative sample that the scientist has to select from a population.
2. **It prevents us, in its strictest sense, from confirming a theory.** While excellent for falsification, the H-D method, taken literally, is exclusively negative: it allows us to be certain that certain theories are false, but prevents us from being certain (or as certain as possible) that a theory is true or probable [33,36]. In other words, even if we have voluminous concordant results, they are incapable of confirming a hypothesis or theory. All they can do is indicate that we have not yet refuted it.
3. **It depends on auxiliary hypotheses.** False-negative results can result from flaws in experimental design or in data interpretation. Moreover, the H-D method also relies on assumptions concerning mechanisms, measuring instruments, laboratory conditions, *et cetera.*
4. **Without appropriate caveats, it is inadequate for certain sciences.** The H-D method is particularly unsuitable for testing probabilistic theories, a variety that, as Mayr [30] pointed out, includes the majority of biological theories. Any exceptions scientists may find to probabilistic theories do not necessarily indicate that those theories have been falsified. In the case of sciences such as evolutionary biology, for instance, it is difficult, and perhaps even impossible, to conclusively falsify certain theories [30].
5. **In its strict sense, it is inflexible to nuance.** For a strict Popperian, a theory or hypothesis can be refuted by a single reliable observation that refutes it [33,35,36]. In other words, the H-D method—combined with radical falsificationism—demands that we categorically abandon a theory or hypothesis if a single well-conducted study yields negative results. This inflexibility may work for the exact sciences; however, as Mayr [30] pointed out, it is inappropriate for sciences such as biology—especially evolutionary biology.
6. **Multiple compatibility problem.** A single set of evidence may be compatible with more than one alternative hypothesis or theory, making it difficult for scientists to determine more easily which explanation is correct. For instance, scientists may use the fact that brain temperature increases during REM sleep [25,37] to support the hypothesis that the function of REM sleep is to regulate brain temperature. However, this same evidence also supports the explanation that such warming occurs due to increased blood flow [38,39]. For this reason, the H-D method often requires that we rely on independent and additional evidence and arguments—a strategy I adopted in this paper to deal with this limitation (See Section “S1” of Appendix A, where I discuss the issue of brain warming during REM sleep.).

Considering its limitations, I decided to complement the H-D method with additional strategies capable of compensating for them, thereby enhancing the rigor of my research. Equally important: I did not adopt the H-D method in its most extreme form, as such an approach is inappropriate in biology—particularly in evolutionary biology (I will return to this point henceforth). This methodological flexibility is necessary when dealing with the real world—a realm full of nuances. We must remember that scientific methods serve to help us elucidate the truth, but not to oppress us. Hereinafter, I detail the strategies I employed to circumvent the limitations of the H-D method. The first of these involves a set of techniques I employed to address the sampling problem, ensuring that the sample is both appropriate and representative. However, before delving into these techniques, I must explain why the H-D method also encompasses an inductive direction.

First, without inductive reasoning, it is difficult (and perhaps impossible) for scientists to formulate general assertions from which they can then derive specific predictions. The general and specific hypotheses derived from the theory—as well as the theory itself—rely on reasoning that moves from particular cases (observational data, patterns of evidence, and analogies with known systems) to general conclusions. Second, the H-D method also depends on auxiliary premises, since one must include assumptions regarding mechanisms, measuring instruments, laboratory conditions, scientific procedures, *et cetera*. It turns out that these auxiliary premises also depend on generalizations—another inductive direction.

Moreover, although strict falsificationists (or radical Popperians) tend to avoid the issue, what one hopes to achieve in testing a theory is to arrive at the objective truth about the world—even if our knowledge of that truth is approximate or incomplete [30,33,40]. After all, this is the very aim of scientific inquiry [30,33,40]. If you disagree, recall that one of the central roles of science is to generate specific predictions upon which practitioners—physicians, engineers, psychologists, pharmacists, neurologists, *et cetera*—can confidently base their work [33].

Therefore, once we have tested a theory developed using the H-D method, we assimilate the positive results, in practice, inductively. This is because we test the specific predictions we deduced from the theory by observing a finite amount of empirical evidence that confirms (or refutes) those predictions. And if the tests corroborate the predictions (a positive outcome), we then extrapolate to assert that the theory possesses validity, corroboration, or truthfulness. That is, in the H-D method, we also generalize the results. In other words, after testing the specific predictions, we generalize from particular cases to assert that the theory has either been corroborated or falsified. (Note that strict Popperians would never agree that a theory has truth or that it has been confirmed [32], p. 10, but I am not one of them.)

Thus, since the H-D approach also entails an inductive direction, this naturally leads us to the sampling problem. That is, my theory requires a body of empirical evidence capable of adequately representing the target population—in this case, the full range of organisms exhibiting REM sleep. Therefore, two critical questions regarding the validity of my theory are (1) What is the appropriate sample size (i.e., the number of studies collected and critically analyzed): and (2) Is this size sufficiently substantial and unbiased to support the generalizations I am proposing. After all, for us to seriously consider any generalization, the sample must be both voluminous and impartial. In the case of this work, this meant I needed to aggregate a sufficient and unbiased *corpus* of empirical evidence. Only then could my theory hold value, being able to accurately encompass and explain the primary function of REM sleep, including its finer details.

As for the first question—“what should the sample size be”—it is evident that there is no magic number, no exact and universal answer. What I must do is clearly explain why I selected the references included here and what the inclusion and exclusion criteria were. To satisfy the criterion of sufficient sampling, I selected 452 bibliographic references (including the references contained in the supplementary material). Considering that many theoretical articles typically contain between 100 and 250 sources, 452 is a large volume. However, volume *per se* is insufficient: large samples are not immune to bias. If properly selected, 452 references constitute a sufficient sample for a theoretical article, capable of conferring a robust and credible character to the theory. Therefore, I needed to adopt strategies to ensure an appropriate bibliographic *corpus*.

In order to minimize, as much as possible, any biases, I adopted the following techniques when selecting the references:

1. ***I prioritized articles that could—directly or indirectly—refute the hypotheses I derived from my theory.*** Since numerous studies easily corroborated many of the hypotheses, they are present throughout the text as corroboration. During my literature search, I also identified numerous pieces of evidence that appeared, at first glance, to refute my hypotheses. However, upon scrutinizing this evidence, I found that it, in fact, corroborates the hypotheses. I devoted Section “S1” of Appendix A to presenting this evidence, as well as my arguments explaining why it corroborates (rather than refutes) the hypotheses. Furthermore, many of the references in Section S1 are there because reviewers pointed them out as refutations of my theory. Again, after scrutinizing them, I was able to demonstrate that they corroborate the theory. (The very fact that I was able to explain—based on empirical evidence—all the apparent refutations proves the value of the theory.)
2. ***I included studies conducted in a wide range of animal species.*** This research technique minimizes bias by fulfilling the criterion to adequately represent biological variation. After all, since I aim to explain the biological function of REM sleep, the empirical evidence must sufficiently encompass the breadth of biological diversity. Some of the animals considered here include: zebrafish, cuttlefish, octopuses, Drosophila, reptiles, Nemestrina monkeys, chimpanzees, humans, rats, mice, birds, sheep, giraffes, cats, guinea pigs, lambs, ferrets, dolphins, belugas, orcas, porpoises, whales, and fur seals. Still within the issue of diversity, the evidence must also include the two principal modes by which the brain rests: sleeping with both hemispheres simultaneously (bihemispheric sleep) and sleeping with only one hemisphere at a time (unihemispheric sleep). Finally, it must also include animals whose sleep includes distinct patterns of temporal organization: monophasic sleep (e.g., humans), biphasic sleep (e.g., birds and insects), and polyphasic sleep (e.g., birds, cats, and many wild mammals).
3. ***I triangulated between multiple sources of evidence, findings, and investigations.*** That is, I incorporated evidence from diverse scientific disciplines (interdisciplinarity). After all, if a theory is supported by evidence originating from various scientific fields, our confidence in it increases, since it is more likely to be true. For this reason, I included evidence from biology, embryology, homology, phylogenetics, genetics, evolutionary biology, physiology, neurophysiology, endocrinology, immunology, neurobiology, neurochemistry, neuropharmacology, ontogenetics, and other areas. With this interdisciplinary approach, I aim to ensure that the theoretical generalization I am proposing derives from an epistemologically robust bibliographic *corpus*, less susceptible to disciplinary or publication biases.

I conducted the research over a five-year period. I began selecting bibliographic sources on February 6, 2021. I amassed a substantial number of references in that initial year of investigation and, over the subsequent years, continued to curate an even larger number of them to discuss in my work. I stopped including and analyzing new references on April 6, 2026. Developing my theory over five years allowed me to refine it progressively as I incorporated new empirical evidence to test it against the hypotheses. To locate peer-reviewed scientific publications, I searched in PubMed and Scopus using keywords related to the topics of my research. While I prioritized more recent empirical research, I also included older research. I did not set any limits on the dates of the research I selected for my work. Furthermore, much of the research I discovered and added to my work was due to its presence in articles I had already selected.

As a selection criterion for sources, I included peer-reviewed publications (scientific articles and book chapters) and a few books written by renowned authors in their respective fields (e.g., Antonio Damasio, Ernst Mayr, Larry Laudan, Richard Dawkins, and Sidarta Ribeiro), with special emphasis on technical books. Regarding the scientific research, I included studies of any kind. For instance, I included Experimental Studies, Theoretical Papers, Comparative Studies, Reviews, Evolutionary Analyses, and Meta-Analyses. I selected the articles especially from well-reputed scientific journals (e.g., Current Biology, eLife, Journal of Sleep Research, Nature Communications, Nature Reviews Neuroscience, Nature, Neuron, PLoS Biology, PLoS One, PNAS, Scientific Reports, Sleep Medicine Reviews, Sleep Medicine, Sleep, and The Journal of Clinical Endocrinology & Metabolism). However, to avoid biasing my research by including only well-regarded journals, I also included research from lesser-known but equally important journals (what matters most, after all, is the quality of the individual article). I excluded non-peer-reviewed scientific articles. That is, I did not include preprints (note that I included two reviewed preprints, from eLife's new publishing model). Finally, although I prioritized English, especially for scientific papers, I did not exclude sources based on language.

To address the limitation of reliance on auxiliary hypotheses in the H-D method, *I assumed that no single study can falsify the theory or its hypotheses*. This is because no study is immune to methodological or interpretive errors. Furthermore, data quality also matters. Therefore, whenever possible, I prioritized citing multiple sources—especially for the most critical arguments. In this way, by drawing on convergent results from replicated research (including via different methods), we can place greater confidence in the evidence. I also gave precedence to studies whose investigators imposed stricter variable controls and included one or more control groups. To minimize (as much as possible) interpretive mistakes on my part, I examined all evidence with the utmost thoroughness of which I am capable. I constructed arguments that align with the current body of evidence, and I was careful to consider alternative hypotheses.

To address the limitation posed by the H-D method's inappropriateness for certain sciences when used without proper caveats, I assumed that, in biology, exceptions are incapable of refuting certain theories [30]. Therefore, even if I found any exceptions, they would be incapable of falsifying the hypotheses or the Sentinel Sleep Theory. Some scientists might consider that I adopt this assumption to immunize my theory against refutation, but they would be mistaken. Given that variation among individuals in a biological population is a cornerstone of evolutionary theory [30,[41], [42], [43], [44]], it would be naïve to presume that exceptions could overturn the theory I am proposing. Indeed, it is precisely because variation exists among animals that non-random elimination can remove those less well adapted [30,[40], [41], [42], [43]].

Moreover, *we must also acknowledge that REM sleep may be more or less optimized in different species*. By this, I mean we should *not* expect REM sleep to be enhanced—or potentiated—in the same manner across the entire animal kingdom. Certain species may exhibit particularly efficient REM sleep, while others may display less efficiency. Let us consider an illustrative example below.

In Section 4, I will argue that REM sleep parameters—in a more optimized form of REM sleep—must adapt to the organism's current level of vulnerability or protection. After all, if an organism is already better protected, it is energetically inefficient to fail to adjust REM sleep parameters. Conversely, if the organism is more vulnerable, it becomes necessary to modify those parameters to further enhance the protective function that REM sleep can provide. The key point here is that this variant of REM sleep is what I call an *optimized version*. That does not imply that all animals possess REM sleep in an optimized form; variations in energetic efficiency may occur, and, consequently, such exceptions cannot falsify the hypotheses concerning the efficiency gains achieved by tuning sleep parameters when an animal already possesses a certain quantity of protection.

Another example is the hypothesis that REM sleep is dispensable in species that sleep with only one hemisphere at a time (see Section 4.3, where I discuss this issue in detail). Given that an active cerebral hemisphere during sleep can confer sufficient protection to the organism, REM sleep becomes unnecessary. However, this *does not* imply that all unihemispheric sleepers entirely lack REM sleep; vestiges may still be present. In this case, the optimized strategy would indeed be to eliminate REM sleep. Yet we must consider that *non-random elimination may not have had sufficient time to achieve this energy-cost optimization*. Therefore, I emphasize again: *we should expect exceptions*. Equally important, these exceptions cannot falsify the hypothesis that it is energetically efficient to eliminate REM sleep when the organism already benefits from the protection afforded by a continuously active cerebral hemisphere during sleep.

Note that the same approach I used to address the H-D method's inappropriateness for certain sciences also served to mitigate its lack of sensitivity to nuance. Finally, to manage the limitation of multiple compatibility of evidence, I drew on additional, independent lines of argument and data. By employing all of these strategies to circumvent the H-D method's limitations, I aim to ensure greater methodological rigor in my work.

For limitations of this work, see the “S2” Section of Appendix A.

## 3 N-REM sleep is highly necessary, but dangerous

Before presenting my theory, I must first engage in a necessary digression: the importance of N-REM sleep. There is still no consensus on the function (or functions) of N-REM sleep. Despite this, it is evident that it serves an essential biological function. *N-REM sleep is not merely a dispensable luxury; it is strictly necessary for the brain, for the body, and for the survival of the organism* [5,18,45,47]. For the brain to function normally, sleep is a necessary condition [5].

A defining characteristic of this behavioral state is the marked reduction in alertness to the immediately surrounding environment [[47], [48], [49], [50]]. Something that clearly distinguishes the state of sleep from the state of wakefulness is the reduced responsiveness to environmental stimuli [48,50,51]. Sleep undermines attention and, eventually, suspends consciousness (in those who possess it) [49,52,53].

As the brain is gradually subjected to deeper sleep (stage 3 of N-REM sleep), its alertness mechanisms are inactivated. *When in the deepest stage of sleep, the brain exhibits the greatest inactivation of its alertness mechanisms (*e.g.*, in the brainstem, anterior cingulate cortex, and thalamus)* [19,49,54,55]. However, this inactivation is not total. Even during N-REM sleep, the brain (albeit mildly) monitors the surrounding environment for potential dangers and can respond differentially to specific prominent stimuli (e.g., unfamiliar sounds) [55,56].

During wakefulness, the organism readily responds to exteroceptive stimuli intercepted by some “sensory portal” (a term used by Ref. [53] that I will borrow here). During N-REM sleep, however, exteroceptive stimuli need to be more intense for the organism to respond to them [50,55]. Therefore, from an adaptive perspective, sleep could seem illogical, effectively a contradiction. The greater neural inactivation characteristic of N-REM sleep—where firing rates and energy use reach their lowest levels during the day—certainly constitutes a substantial risk to the survival of the organism. After all, greater neural inactivation equals greater vulnerability [4,[47], [48], [49], [50],^56^,^57^]. This is why sleeping animals are highly vulnerable to predation [47].

If N-REM sleep did not serve a critical biological function, the central nervous system of countless species would have, over the course of evolution, overcome the need to undergo such a highly vulnerable mental and behavioral state [4,18,47]. Therefore, *the fact that N-REM sleep persisted throughout evolution is because it is strictly necessary* (even if we do not yet know exactly why). Here, I set out to address the biological function of REM sleep, not that of N-REM sleep. Of the latter, only two characteristics are pertinent. The first is that it is present in all animal species with a nervous system, no matter how simple and decentralized it is [45,51,[57], [58], [59]]. The second is that it substantially reduces alertness to the surrounding environment, making the organism highly vulnerable to predation [47].

## 4 The Sentinel Sleep Theory: hypotheses and predictions

Based on the voluminous empirical evidence I collected and analyzed, I developed various explanations to build the conceptual framework of the theory and derived hypotheses and specific predictions. This process involved analyzing vast findings, comparing existing hypotheses, and identifying inconsistencies to form the theory's core assertions. Subsequently, I verified the theory by checking the scientific literature I collected to see if its predictions aligned with the evidence. As the evidence corroborates the predictions, the theory gained strength.

In its most general form, my theory is based on eleven facts and six inferences (see Table 1). The central tenet of the theory is that REM sleep serves to reduce the vulnerability of N-REM sleep. That is why it is in the context of the exacerbated vulnerability and the non-negotiable need for N-REM sleep that we can better understand the function of REM sleep. Another tenet of the theory is that REM sleep parameters (duration, latency, and density) should be modulated by any factors related to protection or vulnerability for the following reasons. (The teleological language I used serves only to explain more easily.)

**Table 1:** Structure of the sentinel sleep theory.

Facts and inferences^a^		References
**Fact No. 1:**	Emotions serve to ensure (directly or indirectly) the organism's survival; among other effects, they make the organism less vulnerable to predation, thereby contributing to its survival. (Emphasis on the fight-or-flight response.)	[52,55,56,[60], [61], [62], [63], [64], [65]].
**Fact No. 2:**	N-REM sleep reduces both environmental alertness and emotional responsiveness, leaving the organism highly vulnerable to predation, thus risking its survival.	[4,47,48,50,51,55,56].
**Fact No. 3:**	N-REM sleep is a non-negotiable necessity for organisms with a nervous system, even if decentralized. In other words, N-REM cannot be eliminated in animals with a nervous system, as it is required for the brain to function properly and for the animal to survive.	[5,18,45,66,46,51,57,58,59].
**Fact No. 4:**	REM sleep currently involves the distinctive neural activation of regions responsible for alertness, attention, and emotional processing (e.g., cingulate cortex, amygdala, hippocampal formation, striatum, and thalamus). In other words, REM sleep is a state of heightened alertness, attention, and emotional responsiveness.	[[67], [68], [69], [70], [71], [72]].
**Fact No. 5:**	Animals show greater alertness after waking up from REM sleep than after waking up from N-REM sleep. This allows a state of high readiness to defend itself from danger.	[19,22,[73], [74], [75], [76], [77]].
**Fact No. 6:**	REM sleep has specific characteristics that allow the animal to awaken quickly after detecting stimuli associated with predators or dangers (e.g., rapid and specific reactivity to predatory stimuli, rapid increase in pupil size, and rapid increase in the ability to move when detecting a predatory stimulus). Which ensures a successful defense against any events capable of threatening the animal's life.	[77].
**Fact No. 7:**	An organism's chances of survival depend on the presence of certain attributes that favor its survival. Thus, not all have the same chances (or probability) of survival.	[30,78,79]
**Inference No. 1:**	The attribute of momentarily increasing alertness, attention, and emotional responsiveness during sleep can contribute to the organism's survival.	Inference 1 is a logical consequence of facts 1 to 5.
**Fact No. 8:**	During REM sleep, brain metabolism increases by approximately 20% due to the heightened intensity of neural activity.	[4,6].
**Fact No. 9:**	Neuronal activity consumes much more energy than neuronal silencing.	[19,80].
**Fact No. 10:**	Natural selection (or non-random elimination) is prolific in removing waste. Nothing so costly tends to last for several million years unless it serves an important function.	[20,81].
**Fact No. 11:**	REM sleep has existed for several million years. Numerous lines of evidence indicate the possibility that REM sleep originated early in animal evolution, approximately 450 million years ago, that is, long before the branch of amniotes.	[12,66,49,51,[82], [83], [84], [85], [86], [87], [88]].
**Inference No. 2:**	The high energy expenditure of REM sleep and its persistence over millions of years imply that it plays an important role in the survival of organisms that possess it. In other words, REM sleep requires a strong evolutionary justification.	Inference 2 is a logical consequence of facts 8 to 11.
**Inference No. 3:**	The primary function of REM sleep is to compensate for the high vulnerability of N-REM sleep. REM sleep is an important biological mechanism that helps increase the organism's chances of survival—a strong evolutionary justification.	Inference 3 is a logical consequence of facts 1 to 11 and inferences 1 and 2.
**Inference No. 4:**	If (for some reason) the organism is more vulnerable and REM sleep parameters do not adapt to compensate for this vulnerability, the protective function of REM sleep will be less efficient, risking its survival.	Inference 4 is a logical consequence of inference 3.
**Inference No. 5:**	If (for some reason) the organism is more protected and REM sleep parameters do not adapt to save energy, the protective function of REM sleep will be energetically inefficient. It will spend resources that could be invested in survival, such as collecting food and seeking shelter.	Inference 5 is a logical consequence of inference 3 and facts 8 and 9.
**Inference No. 6:**	REM sleep parameters (duration, latency, and density) should depend on any factors that affect the organism's protection or vulnerability. They must adapt to conserve energy (when the organism is already protected due to another factor besides REM sleep) or to invest more energy to intensify the protective function (when the organism is vulnerable).	Inference 6 is a logical consequence of inferences 3, 4, and 5 and fact 10.

a “Inference” means a reasoning concluded from the facts listed in the table.

When one is less vulnerable (or more protected), paying the high energy cost invested in the parameters of REM sleep is an unjustifiable strategy. A better strategy is to invest less energy in these parameters, allowing the organism to pay a lower price. In contrast, when one is more vulnerable (or less protected), paying the additional energy cost necessary to intensify the parameters of REM sleep is a justifiable strategy. After all, survival is at stake in this circumstance. In the first case, the high energy cost is unnecessary; in the second, it is utilitarian. Therefore, *for REM sleep to be efficient both as a protective mechanism and in energy consumption, it needs to adapt to any circumstance that reduces or increases the organism's vulnerability*.

Regarding the function of REM sleep, there needs to be a balance between investing energy in the protective function (a justified expense) and saving it when the organism is already more protected for some other reason (an unjustified expense). This implies that the parameters of REM sleep must be determined based on information provided by all varieties of neural maps—interoceptive, proprioceptive, and exteroceptive—because they inform the brain of the current condition of the body and whether any sensory portal detected something capable of affecting the body of the organism, such as a threat [19].

Briefly, **interoception** maps the state of internal organs and the visceral milieu, conveying sensations such as fatigue, pain, hunger, or illness. **Proprioception** maps the state of the musculoskeletal system, providing information about muscle strength, tension, and body position relative to space. Finally, **exteroception** maps the perception of the external environment, particularly regarding its potential impact on the body, such as novelty or the presence of external threats [53]. Concerning the locus responsible for integrating these three mapping systems, the neuroscience literature robustly identifies the insular cortex as a critical neural center for this function [[89], [90], [91], [92], [93], [94]].

Given the central importance of the concept of *vulnerability* to my theory, I need to provide an operational definition. Before doing so, however, it should be noted that vulnerability and protection are two sides of the same coin. This is because stating that an organism is *more vulnerable* is equivalent to stating that it is *less protected*. Conversely, stating that it is *less vulnerable* is equivalent to stating that it is *more protected*. For this reason, throughout this work, I use the terms “protection” and “vulnerability” to refer to distinct and opposing sides of the same coin.

I define *vulnerability* as a neural mapping of the bodily state (i.e., an internal representation) that informs the brain—either automatically or consciously—about the extent to which internal homeostasis has deviated in a given direction. This means that the content forming this state is always relative to the body of the organism in which it arises. It is content that describes and informs about the conditions of biological regulation within the organism, including the state of all internal operations and the current state of its organs [63]. My mechanistic formulation anchors the concept of “vulnerability” not in the external environment (which also includes the environment external to the brain, i.e., the entire body except the brain), but rather in a neuro-homeostatic representation relative to a threat. In other words, it anchors vulnerability in the neural mapping of the bodily state in response to the threat. This implies that, in organisms endowed with consciousness, vulnerability is the *feeling* corresponding to a specific homeostatic state that signals a real, potential, or even imagined threat.

Confronting certain stimuli and events can—actually or potentially—compromise homeostasis. It is due to this danger, even if only potential, that the organism enters an internal state that may appropriately be called *vulnerable* (or *less protected*, if we consider the other side of the coin). One example is an animal moving into a novel environment. Novelty may entail danger, and therefore it is important to respond to it with caution [95]. This is another way of stating that novelty can place the organism in an internal state of perceived vulnerability—even if unconsciously. And how could the organism feel vulnerable without an internal state informing it of exactly that? For this reason, all varieties of neural maps—interoceptive, proprioceptive, and exteroceptive—are central components in the task of mapping and informing the brain about vulnerability (or protection) relative to biological regulation. Such vulnerability may arise both from the internal milieu (the body itself) and from the external milieu (the environment).

Another illustrative example is depression. By leaving the organism with less energy and greater fatigue [56,65,96,97], the neurochemical disruptions of depression place the organism in a state of increased vulnerability. In other words, depression *negatively* deregulates internal homeostasis, contributing to the emergence of a state that I am calling *vulnerable*. And how are these neurochemical disruptions of depression signaled to the brain? This occurs through interoceptive information from the body, which is initially integrated by the brainstem nuclei [63].

There are also cases in which the line between vulnerability and protection is more tenuous. For example, the risk of a threat engenders an internal state in which the organism perceives itself as vulnerable. However, vulnerability stems from the threat and its neural mapping, rather than from the neurochemical and physiological state of the body that arises in response to that threat. Therefore, to avoid ambiguities and misunderstandings regarding my operational definition, *it is necessary to distinguish between the neural mapping that signals the threat and generates the state of vulnerability, on the one hand, and the neurochemical and physiological responses to that threat, on the other*. When an organism perceives an external threat (such as a predator), vulnerability exists as a bodily state that derives directly from the mapping and recognition of that threat and the way in which it disrupts the organism's biological regulation. However, when we analyze the set of neurochemical and physiological responses to that predator, it becomes incorrect to state that this set constitutes a vulnerable state. Quite the opposite, it is precisely these responses that protect the organism's biological regulation and provide it with the chemical means necessary to have any chance of reacting to the threat.

Thus, as the threat escalates, the organism prepares for a fight-or-flight reaction; a response that, in fact, constitutes a mechanism that enhances the individual's protection by providing an internal chemical and physiological means capable of averting the threat and safeguarding life itself [52,60,61,62,64]. Although threats may indeed arise from the external environment (e.g., a predator), it is within the body's internal environment that the signs of such threats are intercepted, mapped, and subsequently signaled to the central nervous system. And then it is mapped again. As a result, the body can then release a cascade of neurochemical and neurophysiological responses to address the threat and maintain homeostasis [52,60,63,64]. This is why it is necessary to dissociate the neural mapping that signals the threat, on the one hand, from the neurochemical and physiological responses to that threat, on the other.

It is evident that, in relation to a threat that has not yet been mapped by the body and brain, the organism will only register that threat and the homeostatic danger associated with it after interacting with it directly. Therefore, the example I provided above should be understood in the context of an organism that has already previously registered the threat.

Moreover, it is also important to distinguish the homeostatic effects of an adaptive and acute stress response (protective) from those of a pathological and chronic stress state (which generates a perception of vulnerability and exhaustion and is associated with depression). A stress response that persists over time ceases to be protective and becomes, in itself, a problematic, negative homeostatic deviation that induces vulnerability. This means that the quality (protective or vulnerability-generating) of the neuroendocrine stress response depends on both its duration and its context.

An important fact that we must consider is that, in organisms that enclose a centralized nervous system and a vast repertoire of innate mechanisms that enable and regulate life (e.g., immune responses, basic reflexes, metabolism, pain and pleasure behaviors, emotions, and feelings), the brain is innately endowed with the capacity to react to certain emotionally competent stimuli [52,[98], [99], [100]]. Therefore, although learning throughout life is important for establishing which stimuli, events, and actions contribute to protecting the organism or to making it vulnerable, we must also remember that many animals are already born equipped with a vast innate emotional repertoire that requires no prior experience [52,[98], [99], [100]]. *Protection and vulnerability, therefore, are guided and modulated by both innate and learned components*.

It should be noted that this definition of vulnerability explicitly requires distinguishing between an objective risk and a neurally represented risk. As will become clearer henceforth, *what matters for my theory and for the parameters of REM sleep is the neurally represented vulnerability, which does not necessarily correspond to an external and objective risk at all times*. Clarifying this is important to prevent any misunderstanding of what I am proposing. An organism can be objectively vulnerable without perceiving itself as vulnerable. This is because, in order to perceive it, the organism must not only already have a neural mapping of the threat but also intercept—at the present moment—some sensory cue of that threat. In the absence of an already mapped threat (in the form of sensory stimuli or cues), there would be, according to my definition, no internal representation of the homeostatic deviation related to that threat. The organism would therefore be subjectively “not vulnerable” according to the criterion I presented, even though it is in imminent danger.

To better understand what I am proposing, the fundamental questions we must ask are these: In relation to what is an organism more or less vulnerable, more or less protected? How are protection or vulnerability translated into neurochemical and neurophysiological terms? What reference does the brain use to determine whether the organism is vulnerable or protected? *The answer to these questions is homeostasis*. Homeostasis is the reference to which the brain turns in order to “know” whether the organism and its internal operations are safe and functional. More precisely, the answer lies in the homeostatic deviation—whether positive or negative—that an event, action, or stimulus causes in the mechanism of biological regulation. Events that disturb the body's regulated equilibrium range induce a state of vulnerability (if the deviation is negative), whereas events that contribute to or defend regulation generate a state of protection (if the deviation is positive). Put differently, certain events and stimuli may disrupt homeostatic regulation by increasing the threat to the organism's life. Conversely, certain events and stimuli may contribute to homeostatic regulation by enhancing the efficiency of biological regulation or by not interfering with it.

And whatever the homeostatic deviation may be, the body will respond with automatic chemical and neural responses that constitute distinct patterns we conventionally call *emotions* [52,63]. The outcome of these emotions is to place the organism in a neurobiological condition that is directly or indirectly favorable to its survival and well-being [52,60,63]. In sum, *an organism's vulnerability is intimately related to its biological regulation*.

**Fig. 1:**
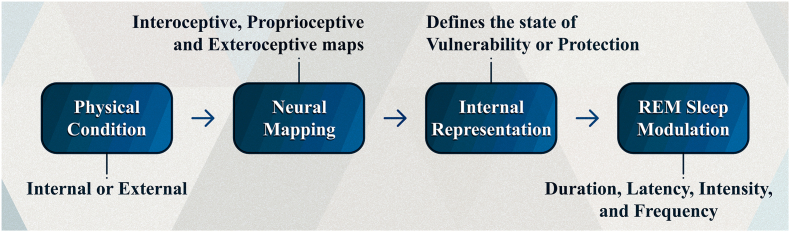
*Diagram of how REM sleep is regulated by internal or external conditions*. The first step is a stimulus, event, or action that occurs inside or outside the body. Then, due to neural and humoral pathways, the brain maps the stimulus. This mapping can be in the form of interoceptive, proprioceptive, or exteroceptive information. In turn, these varieties of neural maps create an internal representation of how vulnerable or protected the organism is. Finally, this homeostatic representation of vulnerability (or protection) modulates the parameters of REM sleep: its duration, latency, intensity, and frequency.

Considering what I just proposed, it becomes evident why it is necessary to dissociate vulnerability from the threat itself (whether external or internal) and to anchor it instead in the mental representation that the brain creates of the body's state in relation to that threat or to any other homeostatic challenge. After all, it is this mental representation—a concrete neurobiological signal—that, once processed by the brain, generates consequences for biological regulation and can affect the circuits that regulate REM sleep. As we shall see in greater detail henceforth, any factor related to protection (or vulnerability) affects the circuits of REM sleep. What I am proposing is that an internal or external condition (e.g., greater muscular strength or exposure to a novel environment) is mapped by the brain (e.g., through proprioceptive or exteroceptive pathways), which then generates a neural representation (not necessarily conscious) of that condition. Finally, this representation modulates the circuits of REM sleep. Below (Fig. 1), I illustrate how REM sleep is modulated.

This formulation (in Fig. 1) provides a neuroanatomical substrate for my theory, postulating that the brain continuously integrates information from three distinct sources to assess vulnerability. These three sources are interoceptive, proprioceptive, and exteroceptive maps. Understanding that REM sleep is modulated by these three varieties of neural maps allows us to understand why it is influenced by any kind of physiological alteration, whether pathological or not. This is because threats to biological regulation may arise either from the body's internal milieu (e.g., an illness) or from the external environment (e.g., a predator). Therefore, given that an organism's vulnerability (or protection) varies depending on the conditions of both the internal milieu and the external environment, the parameters of REM sleep (e.g., duration, latency, and intensity) must necessarily be guided and modulated by the information provided by interoceptive, proprioceptive, and exteroceptive maps.

In sum, my approach to defining vulnerability allows the theory to connect a wide range of factors—from muscle strength to immune status—under a unifying principle: their impact on the organism's biological regulation. The definition I presented situates vulnerability not in the external environment, but in the internal mapping of the body's state in relation to threats. This framework explains why factors as diverse as a novel environment (an exteroceptive challenge), depression (an interoceptive deviation), or muscular weakness (a proprioceptive state) can all predictably modulate REM sleep parameters. All of these factors converge to generate an internal representation of negative homeostatic deviation, which the theory identifies as a state of vulnerability.

Another important issue for my theory to be better understood and evaluated is the role of REM sleep in learning and memory. In the Section “S3” of Appendix A, I argue in detail that failing to recognize that the learning and memory improvements associated with REM sleep are byproducts of its primary function contributes to distancing us from the correct answers.

For my theory to be validated or invalidated, I need to test the veracity of inferences 3 and 6 of Table 1. To do so, I need to list numerous hypotheses derived from my theory and test each of them. If the hypotheses are true, then my theory will also be true. Each of the items below—summarizing the Sentinel Sleep Theory—is a general hypothesis that forms the conceptual body of the theory. I dedicated one Section to each of these general hypotheses (4.1, 4.2, 4.3, 4.4, and 4.5). My goal in each Section is to explain and test the validity of the general hypotheses by verifying the specific hypotheses generated from the general hypotheses (see Table 2). In addition to verifying the veracity of the general and specific hypotheses, I will also demonstrate their factual foundation.

1. ***REM sleep is highly adaptive.*** In the absence of what we happen to call “REM sleep,” the crucial N-REM sleep would leave the organism highly vulnerable. When, by mere chance, a genetic mutation contributed to the emergence of an organism whose vulnerability due to N-REM sleep was reduced, non-random elimination promptly favored this adaptive mutation. And given the high adaptive value of this novelty, it did not remain restricted to the lineage in which it originally debuted. It spread widely across various species.
2. ***REM sleep is cyclical due to its protective function.*** The function of REM sleep—to significantly reduce the vulnerability of N-REM sleep—reaches its full potential when it occurs periodically throughout N-REM sleep, rather than occurring only once.
3. ***The primary biological function of REM sleep is to reduce the vulnerability caused by N-REM sleep.*** While deep sleep is necessary for the brain, it renders the organism substantially vulnerable, compromising its survival. The REM period makes the brain more active—in a state of sleeping vigilance—to increase the organism's alertness to its surroundings, resulting in greater protection. After all, the greater the brain's alertness to the immediate environment, the higher the chances of the organism surviving when a sensory portal detects a sudden threat.
4. ***The parameters of REM sleep depend on the organism's vulnerability.*** The time the brain invests in the REM period, the duration of each episode, its latency (i.e., the period between the onset of sleep and the occurrence of the first REM sleep episode), and its density (or intensity), depend on the current vulnerability (or level of protection) of the organism's body. This is something that is communicated to the brain by all varieties of mental mappings—interoceptive, proprioceptive, and exteroceptive. Generally, the better protected the organism is (lower vulnerability), the less time the brain will invest in REM sleep, and the longer its latency; the less protected the organism is (higher vulnerability), the more time the brain will invest in REM sleep, and the shorter its latency.
5. ***REM sleep probably evolved from a brief awakening from N-REM sleep.*** The most plausible scenario regarding the evolutionary origin of REM sleep is that it emerged from an error. This error caused the organism to briefly wake up from N-REM sleep before its usual awakening, providing a limited but effective adaptive advantage. Consequently, this trait spread and, over the course of species evolution, became more complex. Eventually, this protective mechanism became REM sleep as we know it today.

**Table 2:** General and specific hypotheses derived from Sentinel Sleep Theory.

General hypotheses	Specific hypotheses	Status^a^	References
REM sleep is highly adaptive.	*Hypothesis 1*: organisms that have REM sleep during bihemispheric N-REM sleep have a better chance of surviving than those that do not have it.	Logically confirmed.^b^	[77,101]. (And this article itself.)
	*Hypothesis 2*: REM sleep is a necessary adaptation for organisms that sleep with both cerebral hemispheres.	Factually and logically confirmed.	[12,66,83,85,86,88]. (And this article itself.)
	*Hypothesis 3*: given the high vulnerability of deep sleep (or quiet sleep), there was a strong evolutionary pressure for animals to develop vigilant sleep (or active sleep).	Factually and logically confirmed.	[12,66,83,85,86,88]. (And this article itself.)
	*Hypothesis 4*: predation played a significant role in the evolution of REM sleep.	Factually and logically confirmed.	[48,77]. (And this article itself.)
REM sleep is cyclical due to its protective function.	*Hypothesis 5*: the presence of more than one REM episode offers more efficient protection, increasing the organism's chances of survival.	Logically confirmed.^b^	This article itself. (See the arguments I developed in Section 4.2.)
The primary biological function of REM sleep is to reduce the vulnerability caused by N-REM sleep.	*Hypothesis 6*: REM sleep activates neural regions involved in threat detection.	Factually confirmed.	[4,22,28,52,54,56,71,99,102,[103], [104], [105], [106], [107], [108]].
	*Hypothesis 7*: REM sleep activates neural regions involved in emotional processing.	Factually confirmed.	[4,22,52,54,56,71,99,102,[105], [106], [107], [108], [109]].
	*Hypothesis 8*: REM sleep activates neural regions involved in attention.	Factually confirmed.	[4,22,28,52,54,56,71,99,102,[103], [104], [105], [106], [107], [108], [109]].
	*Hypothesis 9*: REM sleep activates neural regions involved in pain processing.	Factually confirmed.	[19,71,[110], [111], [112], [113], [114], [115]].
	*Hypothesis 10*: REM sleep is necessary when N-REM sleep occurs in both hemispheres.	Factually confirmed.	[[116], [117], [118], [119], [120], [121], [122]].
	*Hypothesis 11*: REM sleep is dispensable when N-REM sleep occurs only in one hemisphere. (Dispensable in the sense that the organism already has sufficient protection provided by an active hemisphere. “Dispensable” does not mean that it cannot appear (with some duration) in some species. Non-random elimination may not have had time to remove REM sleep in organisms in which it makes no sense.)	Factually confirmed.	[[116], [117], [118], [119], [120], [121], [122], [123], [124], [125], [126]].
	*Hypothesis 12*: in organisms that have *only* unihemispheric sleep, REM sleep is useless. Either it does not exist or there are some remnants due to the evolutionary past.	Factually confirmed.	[117,[123], [124], [125], [126], [127]].
	*Hypothesis 13*: in organisms that possess both bihemispheric and unihemispheric sleep, suppression of REM sleep during unihemispheric sleep will generally not accompany REM sleep rebound.	Factually confirmed.	[117].
	*Hypothesis 14*: in organisms that possess both bihemispheric and unihemispheric sleep, suppression of REM sleep during unihemispheric sleep can rarely cause a small rebound of REM sleep.	Factually confirmed.	[117].
	*Hypothesis 15*: upon awakening from REM sleep, the body presents full alertness and sensory and motor efficiency.	Factually confirmed.	[19,22,[73], [74], [75], [76], [77]].
	*Hypothesis 16*: REM sleep makes waking up easier.	Factually confirmed.	[76,77,[128], [129], [130]].
	*Hypothesis 17*: spontaneous awakenings occur more frequently during, or shortly after, REM sleep.	Factually confirmed.	[76,77,[128], [129], [130]].
	*Hypothesis 18*: REM sleep does not suffer a “negative rebound”. Sleeping more one night increases REM sleep time, but does not reduce REM sleep time in the subsequent night.	Factually confirmed.	[76,131].
	*Hypothesis 19*: REM sleep suppression does not significantly compromise any neural function other than the protective function.	Factually confirmed.	[4,19,76,132,[133], [134], [135], [136]].
The parameters of REM sleep depend on the organism's vulnerability.	*Hypothesis 20*: The parameters of REM sleep—its duration, latency to the first episode, and density—depend on information provided by all varieties of neural maps: interoceptive, proprioceptive, and exteroceptive.	Factually confirmed.	[[137], [138], [139], [140], [141], [142], [143], [144], [145], [146], [147], [148],[149], [150], [151], [152], [153], [154], [155], [156], [157], [158], [159], [160], [161], [162], [163], [164], [165], [166], [167], [168], [169], [170], [171], [172], [173], [174]].
	*Hypothesis 21*: total REM sleep time is shorter in organisms with higher body fat.	Factually confirmed.	[141,144,155,170].
	*Hypothesis 22*: the latency to the first REM episode is greater in organisms with greater body fat.	Factually confirmed.	[141,155].
	*Hypothesis 23*: the density (or intensity) of REM sleep tends to be lower in organisms with greater body fat.	Factually confirmed.	[155].
	*Hypothesis 24*: non-obese sedentary individuals tend to present more REM sleep time compared to more active individuals.	Factually confirmed.	[148,165,174].
	*Hypothesis 25*: non-obese sedentary individuals tend to present a shorter latency to the first REM episode compared to more active individuals.	Factually confirmed.	[148,165,174].
	*Hypothesis 26*: non-obese sedentary individuals tend to present greater REM sleep density compared to more active individuals.	Not confirmed nor refuted.^c^	
	*Hypothesis 27*: total REM sleep time tends to be shorter in organisms with greater muscle strength or in those who exercised recently.	Factually confirmed.	[142,143,148,149,158,165,[174], [175], [176], [177]].
	*Hypothesis 28*: the latency to the first REM episode is greater in organisms with greater muscular strength or in those who exercised recently.	Factually confirmed.	[142,143,148,165,174,177].
	*Hypothesis 29*: REM sleep density is lower in organisms with greater muscular strength or in those who exercised recently.	Not confirmed nor refuted.^c^	
	*Hypothesis 30*: recent exposure to a new environment (or new stimuli) increases REM sleep time.	Factually confirmed.	[140,146,147,153,157,159,166,169,172].
	*Hypothesis 31*: recent exposure to a new environment (or new stimuli) reduces the latency to the first REM episode.	Factually confirmed.	[157,159].
	*Hypothesis 32*: recent exposure to a new environment (or new stimuli) increases REM sleep density.	Not confirmed nor refuted.^c^	
	*Hypothesis 33*: depression increases REM sleep time.	Factually confirmed.	[[137], [138], [139],^160^,^167^,^168^,^173^,^178^,^179^].
	*Hypothesis 34*: depression reduces the latency to the first REM episode.	Factually confirmed.	[[137], [138], [139],^156^,^160^,^163^,^167^,^168^,^173^,[178], [179], [180]].
	*Hypothesis 35*: depression increases REM sleep density.	Factually confirmed.	[[137], [138], [139],^151^,^156^,^160^,^167^,^168^,^173^,^180^,^181^].
	*Hypothesis 36*: stress reduces REM sleep time or suppresses it.	Factually confirmed.	[149,164,[181], [182], [183]].
	*Hypothesis 37*: stress increases the latency to the first REM episode.	Factually confirmed.	[181,182,184,185].
	*Hypothesis 38*: stress increases REM sleep density.	Factually confirmed.	[129,182,[185], [186], [187], [188], [189], [190]].
	*Hypothesis 39*: when other factors remain unchanged, combined vulnerabilities produce more intense effects on REM sleep parameters.	Factually and logically confirmed.	[65,76,[96], [97], [191],^137^,^139^,^151^,^156^,^160^,[162], [163], [164],^167^,^168^,^173^,^192^]. (And this article itself.)
	*Hypothesis 40*: when other factors remain unchanged, combined protections produce more intense effects on REM sleep parameters.	Factually and logically confirmed.	[60,188,193]. (And this article itself.)
	*Hypothesis 41*: REM sleep density is a measure of the organism's level of alertness, which is directly related to the amount of stress, because stress reduces the organism's vulnerability by increasing vigilance.	Factually and logically confirmed.	[60,180,[186], [187], [188],^193^]. (And this article itself.)
	*Hypothesis 42*: bodily immature neonates have more REM sleep compared to bodily mature neonates.	Factually confirmed.	[[194], [195], [196], [197], [198], [199], [200], [201], [202], [203]].
	*Hypothesis 43*: bodily immature neonates have a shorter latency to the first REM episode compared to bodily mature neonates.	Not confirmed nor refuted.^c^	
	*Hypothesis 44*: bodily immature neonates have greater REM sleep density compared to bodily mature neonates.	Not confirmed nor refuted.^c^	
	*Hypothesis 45*: in premature births, REM sleep is even more abundant than in newborns.	Factually confirmed.	[196,[204], [205], [206], [207]].
	*Hypothesis 46*: in premature births, the latency to the first REM episode is even shorter than in neonates.	Not confirmed nor refuted.^c^	
	*Hypothesis 47*: in premature births, REM sleep density is even greater than in newborns.	Not confirmed nor refuted.^c^	
REM sleep probably evolved from a brief awakening from N-REM sleep.	*Hypothesis 48*: REM sleep emerged as an error in the neurobiological mechanisms that control the transition from sleep to wakefulness, causing a brief awakening from N-REM sleep.	Logically plausible.^d^	This article itself. (See the arguments I developed in Section 4.5.)
	*Hypothesis 49*: primeval REM sleep evolved from a brief awakening to an ease of awakening.	Logically plausible.^d^	This article itself. (See the arguments I developed in Section 4.5.)
	*Hypothesis 50*: after evolving into an ease of awakening, primeval REM sleep began to include more than one REM episode.	Logically plausible.^d^	This article itself. (See the arguments I developed in Section 4.5.)
	*Hypothesis 51*: intense muscle atonia appeared after primeval REM sleep began to include more than one REM episode.	Logically plausible.^d^	This article itself. (See the arguments I developed in Section 4.5.)

a “Factually confirmed” means that there is empirical support in scientific literature for the specific hypothesis. “Logically confirmed” means that, due to arguments constructed with the best tools of logical reasoning, we have justification to support the conclusion.

b The logical veracity of hypotheses 1 and 5 is a consequence of all empirically confirmed hypotheses, as well as the arguments I presented here for the protective function of REM sleep.

c Requires more research.

d We will never be able to test these hypotheses empirically.

Of the 51 specific hypotheses I listed in Table 2, four of them are logically plausible and can never be tested empirically (hypotheses 48, 49, 50, and 51), seven could not be confirmed or refuted due to lack of studies (hypotheses 26, 29, 32, 43, 44, 46, and 47), two were confirmed logically (hypotheses 1 and 5), and 38 were empirically confirmed. That is, based on the available evidence, *all the empirically testable hypotheses that I could analyze in this article were corroborated; not a single hypothesis was refuted*. The following Sections (4.1, 4.2, 4.3, 4.4, and 4.5) detail the 38 empirically confirmed hypotheses, as well as the hypotheses that I logically tested and those that are logically plausible.

### 4.1 REM sleep is highly adaptive

Oftentimes, it is the information concerning the circumstances and the specific moment in evolutionary history that provides the most crucial clues for understanding the adaptive utility of a trait or behavior [22]. It is for this reason that it is so necessary for me to address N-REM sleep. The scope of the current article does not cover the function of N-REM sleep. However, it is pertinent for me to briefly address its evolutionary origin. As I already stated, the importance of N-REM sleep must be properly understood for the function of REM sleep to be understood as well. I already addressed (in Section 3) the importance of N-REM sleep. Another way to do so is to address its remote origin and persistence over millions of years of evolution since this behavioral state first emerged.

The term “speculate” is often interpreted in a pejorative sense [43], but such a connotation is completely inappropriate in this context. Manifestly, there was no one present to observe the onset of sleep when it first occurred. Moreover, fossils do not include records of organisms' sleep [208]. Therefore, any scientific inquiries into the evolutionary origin of sleep are necessarily speculative. In effect, what interests me is the widely corroborated (and practically indisputable) fact that N-REM sleep is evolutionarily older than REM sleep [50,51,59,76,209,210], even though we are unable to pinpoint exactly when (and in which lineage) it began.

Sleep debuted in invertebrates, as scientists already observed it empirically in zebrafish (*Danio rerio*), fruit flies (*Drosophila melanogaster*), jellyfish (*Cassiopea*), and worms (*C. elegans*) [50,51,59]. Given its predominance in both invertebrates and vertebrates, sleep is certainly a very primeval behavioral state, whose origin may predate the Cambrian Period [76], which extends from about 543 to 485.4 million years ago [211,212].

The fact that sleep was observed even in organisms with relatively simple nervous systems (such as *C. elegans*) implies that *sleep constitutes a necessity for any organism that encloses a nervous system, no matter how simple or decentralized it may be* [51,58,59]. This makes sleep a behavioral state that debuted either concurrently with or shortly after the evolutionary debut of the nervous system. Therefore, the question we must investigate is when the nervous system emerged. Something remarkable about nervous systems and their component units (i.e., the neurons) is that they are highly conserved throughout evolution [211], which manifests the high adaptive value enclosed by them [63]. Despite solid evidence that organisms with nervous systems existed at the beginning of the Cambrian Period, there is still no consensus regarding their evolutionary origin [211].

Paulin and Cahill-Lane [211] estimated that the evolutionary origin of neurons and the nervous system occurred during the Ediacaran Period (the geological period immediately preceding the Cambrian Period), which extends from about 635 to 543 million years ago [213]. This estimate for the evolutionary emergence of sleep implies that over the more than 543 million years of biological evolution since it arose, sleep—despite the vulnerability to which the organism is subjected during its occurrence—prevailed in organisms with a nervous system. Such an imperative, primeval behavioral state, present in all animals with a nervous system, undoubtedly encloses a crucial biological importance.

*Considering the high vulnerability of sleep, what could be done—throughout evolution—to considerably reduce it?* There are two ways to deal with it: (1) non-random elimination removes sleep, or (2) non-random elimination maintains sleep but finds a way to circumvent the problem of the high vulnerability. I will first address the possibility of sleep being eliminated.

Something notable concerning evolution is that any novelties that provide some adaptive advantage (especially if its impact is substantial) enclose a greater propensity to be conserved and spread over time—hence why they become very old [76,78]. The organisms that survive the sieve of non-random elimination in a given generation are those whose genetic constitution engendered a phenotype that possesses what is necessary to survive and reproduce under the prevailing conditions in the specific niche occupied by their species [30,78]. Or, more strictly, to survive and reproduce under the conditions that prevailed in the niche when the ancestral generations of the current members of a given species were subjected to the sieve of non-random elimination [214].

Any properly educated evolutionist knows that non-random elimination is highly effective when it comes to favoring necessary adaptations [30,40]. This means that any necessary adaptation is more prone to spread across various animal lineages, either by debuting independently or by emerging in an ancestral species and remaining throughout its various branches through time [40]. Briefly, every adaptive solution that substantially increments the chances of its bearer surviving and reproducing encloses a high biological value.

The current consensus is that all animals exhibit some form of sleep [57]. Therefore, given the manifest—and substantial—adaptive advantage of a nervous system associated with the absence of the need to subject the organism to sleep, this phenotypic trait, if it existed, would enclose a high biological value. Consequently, the fact that this phenotypic trait *did not* emerge in any lineage is quite revealing. If there were a way to remove sleep from a lineage of organisms without harming them, the genetic information responsible for this phenotypic effect would be strongly favored by non-random elimination.

If it were possible to overcome the need for sleep in the absence of considerable damage to the organism, this phenotypic trait—presence of a nervous system coupled with the absence of sleep—would spread through the various evolutionary branches from the lineage in which it originally emerged. And not only that. Due to the high adaptive value of a nervous system devoid of the need to subject the organism to sleep, this trait would likely emerge independently in various lineages. Since none of these scenarios occurred, we (evolutionists) can conclude, with considerable confidence, that *sleep constitutes an insurmountable necessity for any organism that encloses a nervous system*.

In summary, sleep is too important to be removed from organisms with a nervous system. *Possessing a nervous system inevitably implies the presence of sleep*. And since the solution of removing it is practically impossible, this leads me to the other possibility: finding a way to circumvent the problem of its high vulnerability. What could be done to reduce the vulnerability of sleep? What if the organism's brain, during sleep, underwent considerable neural activation (particularly in regions related to attention, detection of dangerous stimuli, and emotional processing) to make it more alert to the immediate surrounding environment?

The reason for the evolutionary origin of the division of sleep into two periods—N-REM and REM—currently constitutes an enigma to be solved. The question is to elucidate why two sleep states are necessary for the brain [16,50]. A notable aspect of the Sentinel Sleep Theory is that it highlights the answer to this question. N-REM sleep is a non-negotiable biological necessity but encloses a relevant drawback: it makes the organism substantially more vulnerable to predation. Therefore, it is easy to understand that any functionally random evolutionary novelty that led to a significant reduction in the vulnerability of N-REM sleep would inevitably establish itself in the lineage in which it emerged, propagate through various descendant lineages, and would be conserved throughout evolution.

I will present henceforth additional evidence that corroborates my arguments regarding the pressure to develop a way to cope with the heightened vulnerability of N-REM sleep and that REM sleep is a necessary adaptation for those who need to sleep.

Phylogenetic evidence indicates that the central aspects of REM sleep did not evolve independently [16,86,215]. As pointed out by Jaggard and colleagues [66], for more than 50 years, scientists believed that REM sleep was a more recent mechanism, present only in mammals and birds. However, after scientists demonstrated its presence in reptiles, it became believed that REM sleep probably originated in the brainstem of reptiles (the ancestors of birds and mammals) [215]. Several subsequent studies reinforced the fact that at least some reptile species also have REM sleep (e.g., Ref. [216]; Shein-Idelson et al., 2016). The alternation between N-REM and REM sleep in birds, mammals, and some reptiles clearly demonstrates a common origin of these wake-sleep cycle mechanisms, as these animals share a common ancestor [86,215,216].

However, in recent and independent research, scientists showed that, in addition to reptiles, even fish, drosophila, octopuses, and other invertebrate species also have analogs of REM and N-REM sleep [12,66,49,51,[82], [83], [84], [85], [86], [87], [88]]. The above evidence points to the possibility that a state analogous to REM sleep emerged early in animal evolution, long before the branching of amniotes (around 450 million years ago) [66,85]. This initial version would then have become more complex over time until it eventually presented (more recently) the typical characteristics of REM sleep in reptiles, birds, and mammals. (Remember that our estimate of the origin of sleep is that it is over 543 million years old. In other words, the origin of sleep analogous to N-REM sleep remains earlier than the origin of sleep analogous to REM sleep.)

Another possibility is that, instead of a single origin, the REM sleep of reptiles, birds, and mammals and its analogs present in fish, drosophila, octopuses, and other invertebrate species constitute convergent evolution, having debuted independently in evolutionary history [86]. After all, cephalopods (such as octopuses) diverged from vertebrates more than 500 million years ago [86,217,218]. Regardless of the answer—convergent evolution or single origin (homology)—either one strongly corroborates my argument that the sentinel mechanism constitutes a necessary adaptation for any organism that needs to sleep. This phylogenetic and certainly homologous evidence in the case of reptiles, birds, and mammals reinforces my argument regarding the high biological value of REM sleep.

As pointed out by Jaggard and colleagues [66], basic analogs of both quiet sleep and active sleep (the precursors of N-REM and REM sleep, respectively), as well as N-REM and REM sleep themselves, were discovered from humans to fish, and from drosophila to octopuses. Paradoxical sleep (or active sleep)—similar to the wakeful state—exists from mammals to invertebrates [66]. The evidence of active sleep in drosophila, zebrafish, cuttlefish, and octopuses [12,83,85,86,88] indicates a clear selection pressure—due to predation—throughout the course of evolution for the organisms to develop mechanisms that enable the transition from a quieter sleep to a more active (or protective, as I am arguing) sleep.

This evidence corroborates *hypothesis 3* (see Section 4.5 for an in-depth discussion regarding the pressure to develop a way to cope with the high vulnerability of N-REM sleep). It also corroborates *hypothesis 2*, which states that REM sleep is a necessary adaptation for any organism that needs sleep. In fact, the pressure to develop a mechanism to compensate for the vulnerability of quiet sleep is so great that it is possible that many animals developed it independently. The cuttlefish, for instance, is an animal whose analogue to REM sleep may have independently debuted in this invertebrate species [83].

It is crucial to emphasize that the evolution of sleep architecture was not driven exclusively by predation. Other ecological and physiological factors, such as metabolic rate and foraging strategy, also exerted significant selective pressures. However, these factors do not operate in isolation; they are deeply interconnected. For instance, small animals with high metabolic rates are often compelled to adopt polyphasic sleep patterns due to the need to forage more frequently. This same strategy, although dictated by energetic demands, fragments sleep periods and may, secondarily, reduce the window of continuous vulnerability to predation.

Here is the conclusion of the theme of this Section. *REM sleep is a necessary adaptation for any organism that needs to sleep; it is the solution to the problem of the high vulnerability of N-REM sleep*. I demonstrated that the pressure exerted by predation played a significant role in the evolution of sleep. This reinforces the argument that this pressure is much more complex than previously assumed (see Ref. [48]), thus confirming *hypothesis 4*. Regarding *hypothesis 1*, for ethical reasons, we cannot confirm it in the laboratory. But if I demonstrate throughout this article that the primary function of REM sleep is to reduce the vulnerability of N-REM sleep, then the truth of *hypothesis 1* will be a logical consequence of its function. Despite this difficulty, the findings of Tseng and colleagues [77] can be considered empirical and logical confirmations of *hypothesis 1* (I will discuss this article in more detail in Section 4.3.).

### 4.2 REM sleep is cyclical due to its protective function

The problem of vulnerability that REM sleep solves naturally leads me to another much-debated question regarding it: understanding why it is periodically distributed. Or, to put it another way, why sleep is based on cycles that alternate between N-REM and REM sleep throughout the time the organism rests.

The biological function of the alternation between N-REM and REM sleep is currently unknown [219,220]. From the perspective provided by the Sentinel Sleep Theory, the answer becomes apparent. Indeed, explaining why REM sleep is cyclical is easier than addressing its evolutionary origin. When the brain is periodically subjected to REM sleep—the state of dormant vigilance—it enables a more consistent defense for the organism. If the brain were subjected to only one REM episode during N-REM sleep (e.g., at the beginning of the night), the protection offered by the state of dormant vigilance would be significantly reduced. After all, during the remaining time of sleep, the organism would be deprived of this defense mechanism that contributes to reducing the vulnerability experienced during N-REM sleep.

*Making the brain more alert to the immediate surrounding environment only once during the entire rest period is less efficient as a survival mechanism than doing so based on a periodic distribution*. If we compare an organism with only one REM episode to one with multiple episodes, it becomes clear *a priori* which one has a greater adaptive advantage over the other. This is why non-random elimination favored organisms equipped with the genetic information to develop a central nervous system that—rather than undergoing just one episode of dormant vigilance—was subjected to a greater number of such episodes during N-REM sleep. If the function of REM sleep is to reduce the vulnerability of N-REM sleep, the truth of *hypothesis 5* is a logical consequence of this function.

Given that REM sleep lasts less than N-REM sleep, one might argue that my theory is untenable. However, as I detail in the Section “S4” in Appendix A, this is an absolutist way of analyzing the relationship between the adaptive value of sentinel function and REM sleep duration.

Now that I explained the *why*, it is worth explaining the *how*. I need to discuss how the requirement for periodic vigilance integrates with the neural mechanisms that govern the ultradian cycle, such as the *flip-flop switch model*, which involves REM-on and REM-off neuronal populations in the brainstem [221,222]. The Sentinel Sleep Theory implies that this switch is a system modulated by a constant assessment of vulnerability (or protection) carried out by the brain. This is a profound implication, as it allows us to evaluate the progression of the sleep cycle in an unprecedented way. At the onset of rest, deep N-REM sleep (the most vulnerable state) predominates; as rest progresses, REM sleep episodes become progressively longer and more frequent [76,223,224]. Why does this happen? At least two answers are possible—and one does not exclude the other.

1. ***The homeostatic pressure of N-REM sleep progressively reduces.*** As the homeostatic pressure of N-REM sleep is satisfied (especially at the onset of rest), this allows for a greater “budget” to be allocated to the vigilance mechanism that is REM sleep. This explains why the duration and frequency of REM sleep progressively increase. After all, once the organism has invested the necessary time in deep sleep, it then becomes possible to invest more energy and time in the sentinel function to better safeguard the organism.
2. ***The activation of neural regions associated with threat detection interferes with REM sleep.*** Just as the electrical activation of a neural region impacts the memories that reverberate within the mental stream, the reverberating memories can also recruit or modulate the activity of neural regions that would otherwise be less active. That is, it is possible for reverberant activity to affect active, inactive, and less active areas. Due to the activation of regions such as the amygdala, the reverberant content may begin to include negatively valenced memories. In other words, the brain may begin to reverberate memories from the mental database (previously stored) that are related to threatening stimuli. The limbic system then reacts to the emotionally negative content of these dreams. When the content of a dream is threatening, the amygdala (which is active) contributes to engendering a state of vigilance that prepares the organism for a potential awakening. That is, the brain may “believe” that the internal “threat”—in the form of electrically active past memories—is in fact an external threat arising from the environment.

In other words, throughout rest, the frequent activations of the amygdala and other regions related to threat detection may trigger a false sense of danger in the organism due to the contents reverberating in its mind. This is particularly the case because, as I argue in Section “S3” of Appendix A, the memories that reverberate most often are selected based on a neurobiological mechanism that prioritizes those that are more emotionally salient—especially those negatively valenced. Consequently, this virtual danger ends up affecting the parameters of REM sleep: increasing its duration, frequency, and density. In sum, *the internal world of dreams often contains emotionally charged and threatening scenarios. Consequently, the danger that comes from within—from the brain itself—can also affect REM sleep or, more precisely, all of its parameters*. And it is evident that, if this internal danger reaches a certain threshold, the organism will awaken.

The perspective I just presented (regarding the flip-flop switch) has direct clinical applications. In disorders such as insomnia, in which hypervigilance is a central feature [225], the sleep architecture of insomniac patients may reflect a system pathologically inclined toward the detection of potential threats, with a flip-flop switch excessively sensitive to any signals of vulnerability. The result is fragmented sleep and difficulty in initiating or maintaining deep sleep, both typical characteristics of insomniacs [225,226]. Indeed, it is a highly replicated finding that excessive vigilance and the increase of micro- and macro-arousals in insomniac patients are directly related to REM sleep [[227], [228], [229], [230], [231]]. Further evidence for the argument of a flip-flop switch overly sensitive to signals of vulnerability or danger is provided by studies showing that, in insomniacs, sleep exhibits an emotional bias toward negatively valenced stimuli e.g., Ref. [232,233].

### 4.3 The primary biological function of REM sleep is to reduce the vulnerability caused by N-REM sleep

The central function of neurons and the brain composed of them is to assist the body in the intricate task of managing life (i.e., of administering the organism's survival) [52,53,55]. In organisms equipped with a nervous system (which allows the body and any changes occurring within it to be mapped by the central nervous system), *emotions are one of the most biologically valuable processes operating (automatically) to ensure the organism's life* [52,53,55,63,65]. Some stimuli (whether from other animals, objects, or situations) can automatically trigger an emotional reaction. This is why many neuroscientists and psychologists describe them as *emotionally competent stimuli* or, equivalently, that they possess *emotional competence* [19,52,234,235],

In short, emotions are the integration of all the automatic processes (many of which are independent of each other) involved in life regulation that were acquired over evolution [19,53,62]. These processes—which basically consist of complex sets of neural and chemical responses—are triggered whenever the brain receives an emotionally competent stimulus. The presence (real or recalled) of this biologically relevant stimulus (dangerous or valuable), from the internal or external environment, triggers automatic emotional responses [19,52,62,65]. These responses immediately result in altering—momentarily—the state of both the organism's body and the neural structures that map the body. Ultimately, emotional responses serve to place the organism—indirectly or directly—in a circumstance favorable to its self-preservation, survival, and well-being [52,56,62].

If the primary function of REM sleep is to provide the brain with a higher quantity of alertness to the immediately surrounding environment, contributing to the organism's survival, it is evident that there must be significant activation of neural regions involved in attention, threat detection, and emotional processing. And this activation must occur even if it lacks an obvious sense in this context (such as the primary visual cortex, as I will detail further). Before addressing neural activations that make sense, I will start by discussing the most obvious example of activation that—only superficially—seems senseless in the context of sleep.

The primary visual cortex shows intense neural activation during REM sleep, similar to what occurs during the waking state [4,28,76]. The occipital lobes are almost exclusively dedicated to the sense of vision. The most prominent area of the occipital lobes is the *primary visual cortex*, whose function is to receive visual information from the eyes [56]. *Considering that closed eyes during sleep prevent any visual input, what is the purpose of keeping the visual cortex active?* This question led Eagleman and Vaughn [28] to propose the hypothesis that the function of REM sleep is to activate the visual cortex to prevent neighboring neural regions from taking control of it. From the perspective of Sentinel Sleep Theory, the reason why the visual cortex is intensely activated during REM sleep (analogous to activation during wakefulness) is that the eye is an obvious way to detect distant threats.

As a remote sensing “technology,” the eye holds high survival value [40,236]. The adaptive solution we happen to call the “eye” provides the organism with the possibility of remote sensitivity. Instead of being forced to make physical contact with surrounding elements, an organism with vision can, for example, perceive a predator before colliding with it while being chased [236].

Considering the high importance of the eye—during wakefulness—as a radar for threats, the intense activation of regions related to visual processing during REM sleep supports *hypothesis 6* and, therefore, *hypothesis 20*. It is due to the sentinel function that it makes sense for these regions to be substantially active during this sleep state. *The sentinel function also explains part of the reason why rapid eye movements occur during REM sleep*. After all, during wakefulness, the eyes are devices that play a substantial role in detecting threats. It is obvious to us, as conscious observers, that this activation is senseless. Closed eyes do not see and, therefore, are incapable of detecting threats. However, the automatic processes that regulate REM sleep are not conscious agents—nor are the evolutionary processes that shaped them. They are unaware that, although vision is excellent for perceiving threats during wakefulness, it does not operate during the organism's sleep.

In short, due to the protective function of REM sleep (providing greater alertness to the surrounding environment), the occipital cortex (due to its importance as a remote threat detector during wakefulness) ends up being substantially activated during this sleep state. I demonstrated that, according to Sentinel Sleep Theory, the activation of regions involved in visual processing only superficially appears to be senseless. In general terms, any regions particularly responsible for attention and detecting dangerous stimuli play a fundamental role in REM sleep. It is due to their importance for survival that these regions are activated during REM sleep. Thus, my theory offers a more empirically grounded explanation for the high activation observed in the primary visual cortex during REM sleep than the defensive activation hypothesis by Eagleman and Vaughn [28]. Furthermore, the defensive activation hypothesis lacks robust empirical support. Notably, Knopper and Hansen [237] pointed out that recent and important studies do not fully agree with the data that Eagleman and Vaughn [28] provided to support the defensive activation hypothesis.

Now that I addressed this example of neural activation that superficially appears to be senseless, I will address the activation of brain structures that manifestly make sense from the perspective of REM sleep's protective function. One of them is the *cingulate cortex*—a structure that is part of the limbic system. REM sleep, like many attention paradigms, is positively correlated with increased activity in the cingulate cortex [62,71,[110], [111], [112], [113]]. The cingulate cortex plays a crucial role in processes associated with attention, emotional processing, autonomic and endocrine responses to emotions, and consciousness [19,52,62,106,109], corroborating *hypotheses 6, 7, and 8*.

The distinct subregions of the cingulate cortex and its extensive number of somatosensory input signals make this structure capable of potentially engendering the most integrated perception of the current state of the entire body of the organism at any moment. It is a center that integrates emotions, sensations, and actions [52,62,109]. Therefore, it is not surprising that the anterior cingulate cortex is crucially involved in processing emotional states related to pain perception [19,114,115]. The fact that the anterior cingulate cortex plays a crucial role in pain processing is particularly relevant to my discussion. After all, physiological pain encloses a protective function [115]. Therefore, considering the protective function of REM sleep, it is crucial (and expected) that regions processing pain be activated during this sleep period. Thus, the activation of the cingulate cortex during REM sleep also corroborates *hypothesis 9*.

Given that the cingulate cortex receives signals from major sensory portals, it is possible that it contributes to generating a neural pattern that maps, according to the appropriate causal sequence, the relationship between the appearance of a stimulus and the changes occurring in the body in response to it [62]. Upon being perceived, a stimulus can be easily communicated to the cingulate cortex via signals from the thalamus and direct signals from higher-order cortices in the lateral parietal, temporopolar, and inferotemporal regions [62].

These characteristics make the cingulate cortex highly appropriate for the protective function exercised by REM sleep. The integrated perception of the body's state enabled by the cingulate cortex, as well as the pain processing carried out by this neural region, is very useful in the context of REM sleep. Since N-REM sleep is a state of high vulnerability, the increased neural activation of the cingulate cortex during REM sleep allows the brain to better analyze the organism's current state. Therefore, this structure crucially contributes to the protective role played by REM sleep.

Another brain structure whose activation makes sense from the perspective of the protective function of REM sleep is the *amygdala*. After all, it is a fundamental structure for detecting threats and triggering physiological and behavioral responses to danger. The amygdala is so important for vigilance and attention that, when electrically stimulated in certain areas, it puts the brain into an even more intense state of vigilance and attention [4,103,104,238]. Additionally, it also plays a crucial role in both emotional processing and the regulation of the arousal state [6,17,239]. The amygdala occupies a privileged position in the brain. When it detects a threat, it quickly dominates the rest of the brain—especially the prefrontal cortex—to project the organism's attention onto whatever the threat is [238].

Considering all these facts, as well as the fundamental role of emotions as the managers of life [52,53,63], and that arousal refers to the condition in which the organism is alert to the surrounding environment [240], it is entirely appropriate that the amygdala is involved (and with a prominent role) in REM sleep. In fact, based on the function of detecting threats and triggering physiological and behavioral responses to danger, we can predict that *the amygdala plays an important role in the regulation of REM sleep*. Based on this prediction, a strong correlation between REM sleep and the intense activation of the amygdala is expected.

Evidence supports this prediction: the amygdala plays an important role in the regulation of REM sleep [239] and is much more intensely activated during REM sleep than during wakefulness [4,54,71,102,105]. The central importance of the amygdala to REM sleep is also evident when we analyze what happens when this structure is inhibited. Tetrodotoxin (a potent neurotoxin) can temporarily inhibit the action of neurons and tracts. When applied to the central nucleus of the amygdala, tetrodotoxin inhibits it. The consequences of this are revealing: a significant reduction in REM sleep duration and the number of REM episodes [239,241]. A scrutiny of the functions of the amygdala will allow me to demonstrate more clearly why it plays a central role in REM sleep.

The amygdala plays a crucial role—during wakefulness—in assessing the valence of received stimuli and, if negative, triggering the appropriate responses to ensure the organism's survival [52,56,99]. The amygdala is particularly relevant to survival because it performs the function of receiving and learning about biologically relevant stimuli, especially emotionally competent stimuli with negative valence—exactly those crucial for survival. This is why activity in the amygdala is more closely associated with the emotion of fear [4,19,52,56,99,107,108]. Part of the amygdala's function is to associate an external stimulus with its consequence for the organism, whether that consequence is positive (a reward) or negative (a punishment), encompassing all gradations between these extremes. Putting it another way, the amygdala also serves to assign valence (a biological value) to received sensory stimuli [19,99,107,242].

Due to its sparse connections with cortical areas, the amygdala can influence the action of other neural regions, which is equivalent to saying that it can influence the action of other cognitive functions (e.g., modulate attention and perception) [19]. When the amygdala receives an emotionally competent stimulus (e.g., through neural projections from visual cortices), this stimulus is analyzed for its valence to determine the presence or absence of danger. If the valence of the stimulus is negative (i.e., if it consists of a threatening stimulus), the amygdala is activated. When this happens, it triggers the appropriate cascade of physiological and behavioral reactions (e.g., changes in heart rate, respiratory rate, pupil dilation, cutaneous blood flow, sweating, and facial muscle movements). It can accomplish all this by signaling to other neural regions (e.g., brainstem, hypothalamus, cingulate cortex, somatosensory cortices, and monoaminergic nuclei) and to the body (e.g., endocrine glands, viscera, and musculoskeletal system). This set of reactions is what we happen to call *emotion*s [17,19,52,53,56,62,108,243,244].

Physiological and behavioral reactions triggered by the amygdala serve the purpose of safeguarding the organism [19,56,108]. Therefore, it is particularly relevant that information from all sensory portals is projected to the amygdala, with each sensory portal having a distinct projection pattern. It is the interconnections within the amygdala that allow information from different sensory portals to be integrated [4]. All of this previous evidence regarding the characteristics of the amygdala makes it highly suitable for the protective function performed by REM sleep, and supports *hypotheses 6, 7, and 8*. Thus, it is not surprising that the amygdala plays a central role in the regulation of REM sleep. The sentinel function of REM sleep allows me to easily explain both the intense activation of the cortical amygdala during this sleep state and its distinctive regulatory role.

To prevent anyone from misinterpreting my arguments, I want to emphasize the following. It might seem that I am employing circular reasoning when I claim, for example, that the distinctive activation of the amygdala during REM sleep corroborates the sentinel function of REM sleep. As if I were using the premise of the sentinel function of REM sleep to conclude that the amygdala being active during REM sleep corroborates the sentinel function. This would be a serious misinterpretation of my arguments. What I am actually using as a premise is the well-known fact that the amygdala performs a protective function *during wakefulness*. Consequently, its distinctive activation during REM sleep corroborates the sentinel function of REM sleep. There is no circularity here. And the same applies to the arguments I developed regarding the activation of the cingulate cortex and other neural regions during REM sleep.

For the sentinel function of REM sleep to be performed, it is necessary that the regions responsible—during wakefulness—for attention, vigilance, and emotional processing be activated during REM sleep. It is already well-documented in the scientific literature that limbic structures exhibit high neural activation during REM sleep [68,70]. Through Positron Emission Tomography (PET), Statistical Parametric Mapping (SPM), and neuroimaging studies, scientists demonstrated that numerous regions of the limbic system—emotion-related regions—are differentially active during REM sleep. The cingulate cortex (especially the anterior region), both amygdaloid complexes, the hippocampal formation, the striatum, and the left thalamus experience an increase in both blood flow and electroencephalographic activity during REM sleep [67,69,71,72].

Moreover, not only does the limbic system become prominently more active during the REM period, but the paralimbic structures also exhibit high neural activation during this sleep period [67]. The amygdalofugal pathways to the right parietal operculum, thalamic nuclei, entorhinal cortex, dorsal midbrain, pontine tegmentum, and anteroinferior portions of the insula are also notably activated during the REM period [67,69,70,245]. This heightened activation of the limbic system during REM sleep—the set of neural regions involved in emotional processing—as well as the paralimbic structures (also involved in emotion), is precisely what the sentinel function of REM sleep predicts.

A brief digression is necessary. The higher-order neural regions involved in emotional processing have traditionally been grouped under the label *limbic system* [17,246] Despite the term “limbic system” still being widely used in discussions concerning the neural mechanisms responsible for emotions, it is important to note that there is no single emotional system [4]. Some neural structures undoubtedly involved in emotional processing (e.g., the anterior cingulate cortex, the amygdala, and the insula) also have other functions [4,242]. In this case, therefore, there is no one-to-one correspondence between a neural region and a function [4,5].

Indeed, given the high biological value of emotions, any evolutionary biologist can easily perceive how the strategy of a one-to-one correspondence between a neural region (or system) and an emotional function would, in all likelihood, have been eliminated. After all, it is not an *Evolutionarily Stable Strategy* (ESS). It is biologically advantageous for emotional processing to be divided among various regions. This way, when one of them is compromised, the others can still perform the task.

Another brain structure whose activation makes sense is the *thalamus*. After all, among other functions, the thalamus is involved in attention and alertness [[247], [248], [249]]. This structure is so important for attention that damage to higher-order thalamic regions—such as the mediodorsal nucleus and the pulvinar nucleus—can result in severe attention deficits (e.g., Ref. [250,251]). Therefore, considering *hypothesis 8*, the thalamus should be active during REM sleep. Functional neuroimaging demonstrates that during the N-REM period, the thalamus is inactivated. However, during REM sleep, the thalamic nuclei are activated [252,72], confirming *hypothesis 8*.

Additional evidence related to alertness and vigilance comes from the Hypothalamic–Pituitary–Adrenal (HPA) axis: a system that connects the hypothalamus, the pituitary gland, and the adrenal glands, and is responsible for regulating the release of hormones that play a crucial role in the physiological stress response (primarily cortisol and adrenaline) [238]. For the protective function of REM sleep to operate, this physiological state must be associated with activation of the HPA axis. After all, one of the central functions of the HPA axis is to keep the organism alert and vigilant [61,[253], [254], [255]]. The evidence shows that REM sleep is indeed associated with HPA axis activity, and that they mutually influence one another [[256], [257], [258], [259]].

According to my theory, this influence operates bidirectionally for the following reason: REM sleep, due to its protective function, needs to be influenced by the HPA axis given that this axis maintains a state of alertness and vigilance. In turn, REM sleep can affect the HPA axis because of the content the brain reverberates during the REM period. After all, evoked memories also trigger emotions. And if those evoked memories—due to their emotional content—demand action from the HPA axis, then it will be activated.

To continue with the factual foundation of the Sentinel Sleep Theory, I will now analyze unihemispheric sleep. This will allow me to test, through a “natural experiment”, a key prediction of my theory. As will become evident, the fact that REM sleep almost *never* occurs in a brain undergoing unihemispheric sleep strongly supports my arguments about the protective function of REM sleep. This evidence provides powerful support for my claims that REM sleep is a necessary adaptation specifically for organisms undergoing bihemispheric sleep, where both hemispheres are simultaneously vulnerable.

For certain animals, the environmental pressure against the brain being subjected to sleep in both cerebral hemispheres is so substantial that they ended up developing, through non-random elimination, unihemispheric sleep [4,17,76]. Their brains can sleep using only one cerebral hemisphere at a time [76,118]. In certain environments and niches, if the organism's brain were subjected to N-REM sleep in both hemispheres, the organism would face serious problems. Its survival would be severely compromised—either due to heightened vulnerability caused by the low levels of alertness characteristic of N-REM sleep or due to the need to maintain movement [76,118].

Unihemispheric sleep allows only one hemisphere to undergo much-needed N-REM sleep. Putting it another way, *unihemispheric sleep prevents both hemispheres from becoming significantly more inactive and, consequently, prevents the organism from becoming significantly more vulnerable* [19,76,118]. In unihemispheric sleep, the neural mechanisms involved in promoting the waking state predominate in one cerebral hemisphere (as indicated by desynchronized electroencephalographic activity with high-frequency and low-amplitude waves), while the neural mechanisms involved in promoting the N-REM sleep state predominate in the other (as indicated by low-frequency and high-amplitude waves) [76,118,260]. Due to this evolutionary strategy, one hemisphere can lower its alertness (an imperative characteristic of N-REM sleep) while the other hemisphere ensures that vigilance and attention to the surrounding environment are maintained—preventing the organism from being subjected to substantial vulnerability.

For cetaceans (e.g., dolphins, belugas, orcas, porpoises, and whales), unihemispheric sleep constitutes the only form of sleep [76,118]. This characteristic allows cetaceans to maintain constant movement, ensuring periodic surfacing for breathing [76,118]. Repeated studies on cetaceans failed to find any amount of REM sleep in these animals [117,[123], [124], [125], [126]]. The list of parameters used to analyze sleep in bottlenose dolphins (the most studied cetacean species) includes brain temperature, respiratory rate, electrocardiogram, eye movements, electromyogram, lateral geniculate bodies and hippocampi, and the electroencephalogram of the cortical hemispheres [261,262]. Only a single study in the literature demonstrated the presence of REM sleep in the pilot whale, which lasted only 6 min and occurred only once [127]. Thus, cetaceans either have a negligible amount or no REM sleep [123]. Therefore, this evidence corroborates *hypotheses 11 and 12*.

The fact that cetaceans lack REM sleep has been interpreted as evidence that the need for REM sleep is overridden if the brain maintains, in one of its hemispheres, elevated levels of electrical activity capable of sustaining continuous motor activity and a high level of alertness [76]. I argue, based on my theory, that this observation is entirely correct. Since the function of REM sleep is to provide greater defense to the organism during the vulnerable N-REM sleep, its absence in cetaceans is further evidence in support of the Sentinel Sleep Theory. After all, with the unilateral occurrence of N-REM sleep in these animals, there is sufficient neural activation to ensure consistent defense against any threats in the surrounding environment, making REM sleep unnecessary. For REM sleep to exist in animals that sleep with only one hemisphere would be a huge waste of energy.

As we know, cetaceans evolved from terrestrial mammalian ancestors [263,264]. When we combine this fact with *all* the arguments and evidence I presented here (in this article) to demonstrate the function of REM sleep, it becomes evident that the ancestors of modern cetaceans gradually lost REM sleep because it became dispensable for the reasons I elucidated above. Therefore, it is clear that current cetaceans may exhibit remnants of REM sleep from their ancestors—perhaps too subtle to be detected with the methods currently employed. However, if we could travel back in time and analyze the sleep of cetacean ancestors, we would observe increasing remnants of REM sleep until it became evident, as we approached the branching point from terrestrial mammals, manifesting as the distinct REM sleep known in terrestrial mammals. Therefore, for *hypotheses 11 and 12* to be corroborated, it is not necessary for cetaceans to be entirely devoid of REM sleep, as remnants of it are expected due to their evolutionary history.

Unlike cetaceans, other animals have both unihemispheric and bihemispheric sleep. Birds are examples of this [15,265]. A relevant fact for my discussion is that REM sleep occurs in them especially when the brain is subjected to bilateral N-REM sleep; *in birds, REM sleep is absent or occurs in very small amounts whenever unihemispheric sleep occurs* [116,[118], [119], [120], [121], [122]]. This corroborates *hypotheses 10 and 11*. The same explanation I presented for cetaceans in the previous paragraphs applies to birds. When a bird's brain is subjected to unihemispheric sleep, there is sufficient neural activation to ensure environmental vigilance. However, the same does not happen when the brain is subjected to bihemispheric N-REM sleep. That is why REM sleep is present in birds when they undergo bihemispheric N-REM sleep. I will discuss henceforth another animal that has both bihemispheric and unihemispheric sleep.

The northern fur seal (*Callorhinus ursinus*) is a semiaquatic mammal: it can sleep both in seawater (where it spends most of its life) and on land [117]. Lyamin and colleagues [117] demonstrated that when the studied fur seals slept in water, REM sleep was either effectively suppressed or significantly reduced: from 80 min (when on a dry platform) to 3 min per day (when in water); a reduction of 96.4%. During the first three to seven days in water, no REM sleep was recorded in any of the fur seals; in one of the four fur seals, REM sleep occurred on only one of the eleven days of analysis. After undergoing this almost complete suppression of REM sleep and returning to sleep on the dry platform, the fur seals either exhibited minimal REM sleep rebound or no rebound at all. When the fur seals left the dry platform and returned to the water, bihemispheric sleep was replaced by unihemispheric sleep. While in seawater, their N-REM sleep was predominantly unihemispheric (94% of all N-REM sleep was unihemispheric in this condition). In comparison, when on the dry platform, unihemispheric N-REM sleep was reduced (61% of all N-REM sleep was unihemispheric in this condition). *And again (as with birds), unihemispheric N-REM sleep was associated with the absence of REM sleep*.

From the perspective of the Sentinel Sleep Theory, the reason the fur seals did not exhibit REM sleep rebound (or exhibited minimal rebound) is due to the biological function of REM sleep. Since their brains were predominantly subjected to unihemispheric sleep while they remained in water, the fur seals were sufficiently protected. Their brains were sufficiently vigilant to the surrounding environment. Thus, REM sleep was dispensable. As I already stated, REM sleep is necessary only when N-REM sleep occurs in both hemispheres.

Regarding the minimal REM sleep rebound observed, it may be due to the following reason. The fur seals clearly enclose neural mechanisms that control REM sleep suppression, activated whenever unihemispheric sleep occurs. As I will elaborate further, REM sleep rebound constitutes a defense mechanism triggered whenever REM sleep is suppressed. It turns out that in this case there is conflicting information. On one hand, whenever unihemispheric N-REM sleep occurs, the organism is protected, making REM sleep dispensable. On the other hand, whenever unihemispheric N-REM sleep occurs, REM sleep is suppressed, making REM sleep rebound necessary.

Therefore, the reason behind the minimal rebound observed may simply be because REM sleep was suppressed when the organism's brain was subjected to unihemispheric N-REM sleep. However, since the organism was sufficiently protected by being subjected to N-REM sleep in only one hemisphere, the rebound was minimal instead of lasting as long as the suppression occurred. We must consider that non-random elimination may not have had time to eliminate this rebound when it makes no sense to have it. Therefore, it is expected that many animals will present minimal rebound even after their brain is subjected to unihemispheric N-REM sleep. Thus, the study by Lyamin and colleagues [117] corroborates *hypotheses 10, 11, 13, and 14*.

Another fact that corroborates the Sentinel Sleep Theory is the way organisms respond when awakened from REM sleep. When an organism (human or non-human) is awakened from REM sleep, it exhibits full alertness (an obvious adaptive advantage) [19,76,77]. The biological relevance of the REM period is evident from the fact that animals, when awakened during this period, respond more effectively and demonstrate superior sensory and motor function compared with those awakened from N-REM sleep—who exhibit sensory, cognitive, and motor deficits that take several minutes to dissipate [19,22,[73], [74], [75], [76], [77]]. And in addition to the fact that there is empirical support in non-human animals for sentinel sleep [77], my theory also has empirical support in humans [101,266]. All this evidence corroborates *hypothesis 15*. If the biological function of the REM period is to reduce the vulnerability of N-REM sleep, then the heightened readiness demonstrated by organisms awakened from REM sleep is precisely what would be expected. This readiness is a consequence of the sentinel function of REM sleep.

Additionally, another relevant fact is the habitual occurrence of spontaneous awakenings during or immediately after REM sleep, which led scientists to believe that the REM period serves to facilitate the transition from N-REM sleep to wakefulness [76,[128], [129], [130]]. All the aforementioned evidence corroborates *hypotheses 16 and 17*.

Considering the protective function of REM sleep, we must expect animals to wake up during REM sleep if they detect a stimulus associated with a predator. The study by Tseng and colleagues [77] provides robust evidence supporting this prediction. In their research, the authors tested whether animals react to predator stimuli during REM sleep. Among other findings, they observed that animals exposed to predator stimuli woke up during REM sleep but not during N-REM sleep. Furthermore, the researchers also demonstrated that the intensity of alertness during REM sleep was higher. Another relevant finding reported is that the same neurons fundamental for defensive responses during wakefulness are activated during REM sleep. All these results indicated, as Tseng and colleagues stated, that REM sleep enables rapid awakening in response to predatory stimuli, ensuring a successful defense against any threats to the animal's life. All these findings corroborate *hypotheses 1, 4, 15, 16, and 17*.

To conclude this Section, I will discuss what happens when REM sleep is suppressed. Organisms that undergo total REM sleep deprivation experience a vigorous compensatory return known as *REM sleep rebound*. This rebound is characterized by a subsequent increase in both the time the brain invests in the REM period and the intensity of this period, leading to more intense intrusive dreams [19,76]. REM sleep rebound is proportional to the duration of its suppression, but—and this is particularly relevant to my arguments—*the opposite is not true*. As affirmed by Ribeiro [76], increasing N-REM sleep time also increases REM sleep, but it does not cause a subsequent “negative rebound”, which corroborates *hypothesis 18*. The reason this negative rebound does not occur is obvious from the perspective of the Sentinel Sleep Theory: doing so would compromise vigilance during sleep and, consequently, the organism's safety.

REM sleep rebound is due to its sentinel function. This biological mechanism that provides greater protection during sleep—the REM period—proved to be so fundamental throughout evolution that it is present in a vast number of distinct species. Due to its biological value, major or total suppression of this protective mechanism represents an abrupt increase in the organism's vulnerability during N-REM sleep. When the brain is subjected to major or total suppression of the REM period, it activates a defense mechanism: REM sleep rebound. If REM sleep is suppressed, the brain demands a subsequent compensatory investment in REM sleep to offset the heightened vulnerability it was exposed to during REM sleep suppression. *The evolutionary pressure to develop a protective sleep was so high that even this protective sleep has a protective mechanism: REM sleep rebound* (See Sections 4.1 and 4.5 for a deeper discussion of this evolutionary pressure.).

The sentinel function of REM sleep explains why its suppression (partial or total) does not result in neural or cognitive impairments for the organism. Contrary to what is claimed by many scientists [4,21,56,76], the primary function of REM sleep is *not* to contribute to learning, but rather to provide greater protection to the highly vulnerable N-REM sleep (See Section “S3” of Appendix A, where I justify this assertion.). This is why patients medicated with antidepressants can exhibit near-complete or complete REM sleep inhibition for years without showing any notable deficits in learning and the capacity to form new memories, while maintaining normal brain functionality. REM sleep inhibition is an effect caused by practically all antidepressants, with some even interfering with the homeostatic regulation of REM sleep [4,19,76,[132], [133], [134], [135], [136]]. These facts corroborate *hypothesis 19*.

The greatest harm that REM sleep inhibition causes to an organism is the substantial increase in its vulnerability during sleep. Therefore, it is completely possible that REM sleep suppression does not significantly compromise any neural function other than the protective function it provides. Note that the preceding statements refer *exclusively* to REM sleep suppression. It is crucial to distinguish between the effects of exclusive REM sleep suppression and REM sleep suppression accompanied by N-REM sleep suppression. We must consider this distinction because, as Lyamin and colleagues [123] stated, it is common for scientists to also suppress N-REM sleep when studying REM sleep suppression.

I understand how the statement that “REM sleep suppression does not significantly compromise any neural function other than the protective function” is too strong. However, considering all the evidence supporting my theory, this conclusion seems correct to me. I consider it important to clearly state what seems correct to assert based on the evidence and my theory. This way, other scientists can test my assertion to determine whether I am wrong or not. Based on this work, this strong statement must be thoroughly tested henceforth. Only then will we know for sure. Therefore, we should be careful with this particular statement until we know the answer for sure.

### 4.4 The parameters of REM sleep depend on the organism's vulnerability

This Section contains potentially the most innovative and empirically rich contribution of my theory: the hypothesis that REM sleep parameters (duration, latency, density, and frequency) are dynamically modulated by the organism's neurally-represented state of vulnerability. This concept is the engine of my theory, and its power lies in the operational definition of vulnerability I presented in Section 4.

An obvious prediction of the Sentinel Sleep Theory is that the brain of organisms with greater body fat or muscle strength will spend less time in the REM period. After all, greater weight or muscle strength leaves the organism more protected compared to its peers lacking this protection. An organism with lower body weight or lower muscle strength is more vulnerable compared to another organism of the same species with greater weight or muscle strength. This is why increasing muscle strength or weight should be accompanied by a reduction in the time the brain invests in REM sleep. Furthermore, a longer latency to the first REM period is also predicted. In less vulnerable organisms—either due to a greater amount of body mass or greater muscle strength—the onset of the first REM period can delay beyond the usual time. Since the organism is better protected, the brain can dedicate more time to the fundamental N-REM sleep before transitioning to the sentinel stage. Finally, a lower density of REM sleep is also predicted in less vulnerable organisms.

Before proceeding, I need to justify these statements. In humans, domestic animals, and laboratory animals, muscle atrophy typically limits mobility and physical performance and increases the likelihood of injury as the animal ages. In contrast, *in wild animals, muscular performance can mean the difference between life and death*. After all, muscle atrophy reduces the physical capacity to avoid predators, directly affecting the animal's chances of survival [267,268]. Muscle strength is, therefore, a variable directly related to protection (when muscles are developed) or vulnerability (when muscles are atrophied).

Regarding body mass, it is also related to fitness; reducing it can significantly impact physical fitness in both the short and long term [269]. Moreover, it is easy to understand that an organism with a higher amount of body fat is physically more protected (or less vulnerable) compared to a leaner one. *Concerning the physical blows needed to take down a prey, it is more challenging for a predator to kill a fat prey than a lean one*. A predator that sinks its teeth or claws into a lean prey can more easily reach its vital organs. The same cannot be said for a fat prey. Body fat is, therefore, a variable directly related to protection (when there is a lot of body fat) or vulnerability (when there is less body fat).

However, we must consider that weight increases protection only up to a certain point. The correlation between higher body fat and greater protection is not linear; it is a curve. Beyond a certain point, excess weight begins to hinder the body more than help protect it. Ultimately, we must consider that weight affects some important abilities, such as the capacity to escape.

Among the hypotheses related to how body fat and muscle strength affect REM sleep parameters, except for *hypotheses 26 and 29*, all other hypotheses (21, 22, 23, 24, 25, 27, and 28) are supported by empirical research [[141], [142], [143], [144],^148^,^149^,^152^,^155^,^158^,^165^,^170^,[174], [175], [176], [177]]. Therefore, all this evidence also corroborates *hypothesis 20*. In the Section “S5” of Appendix A, I discuss some of the above studies in more detail, analyzing them from the perspective of Sentinel Sleep Theory.

Considering the sentinel function of REM sleep, another prediction is that organisms exposed to an unknown environment (and therefore rich in sensory information) should show a significant increase in REM sleep time, a shorter latency to the first REM episode, as well as a greater intensity. After all, *the unknown includes the possibility of danger*. This is equivalent to stating that an unfamiliar environment subjects the organism to greater vulnerability. As described by Kahneman [95]:

To survive in a frequently dangerous world, an organism should react cautiously to a novel stimulus, with withdrawal and fear. Survival prospects are poor for an animal that is not suspicious of novelty. However, it is also adaptive for the initial caution to fade if the stimulus is actually safe.

When the organism rests in a familiar environment, the brain benefits from this familiarity, especially if the environment does not include (in recent experiences) a constant level of dangerousness. Under this condition, the brain can invest less time in the REM period and may even delay its onset slightly (longer latency). However, when the organism is in an unknown resting place, vigilance against any possible threats needs to be higher. This is why, whenever the organism is exposed to an unknown environment, the brain will invest more time in the REM period, its intensity will be greater, and it will be more imperative that it does not delay its onset (shorter latency). *The possibility of danger demands a greater amount of REM sleep, a shorter latency to the first REM episode, and a greater intensity of REM sleep*.

It has been consistently demonstrated that exposing an animal to a rich sensory experience during wakefulness (e.g., being exposed to a new environment) significantly increases the time the brain invests in REM sleep and reduces REM sleep latency (in some cases, without altering total sleep time) [140,146,147,153,157,159,166,169,172]. This evidence corroborates *hypotheses 30 and 31* and also implicates fear as an emotion capable of affecting REM sleep, corroborating *hypothesis 20*.

A not-so-obvious prediction of the Sentinel Sleep Theory is that, besides body mass and muscle strength, any other factors that increase or decrease the organism's vulnerability will also affect REM sleep. After all, it is not only body mass and muscle strength that influence the organism's vulnerability; other factors can also make it more or less vulnerable. It is possible to extend the discussion beyond the obvious factors. This leads me to discuss stress and depression. I will start with depression.

A notable characteristic of depression is that it places the organism in a state of increased vulnerability—leaving it with low energy and greater fatigue (proprioceptive information that the brain maps) [56,65,96,191,97]. Fatigue is so common in depressed patients that it occurs in more than 90% of patients [[270], [271], [272]]. Therefore, according to the Sentinel Sleep Theory, depression should cause the brain to invest more time in REM sleep, reduce the latency to the first REM episode, and increase its density (or intensity). It may also cause the first REM episode to be longer. When the organism is more vulnerable (e.g., due to depression), the first REM episode may last longer precisely because of this vulnerability. Since N-REM sleep predominates at the beginning of sleep [76], this vulnerability combined with another vulnerability (e.g., depression) may result in a longer first REM episode. *When other factors remain unchanged, combined vulnerabilities produce more intense effects on REM sleep parameters*.

All these predictions were consistently confirmed (although not under the context of my theory). Depressed patients exhibit a decrease in N-REM sleep, an increase in total REM sleep time, shorter REM sleep latency, a prolonged first REM episode, and greater intensity (or density) of REM sleep (especially in the first REM period) [56,[137], [138], [139],^151^,^156^,^160^,[162], [163], [164],^167^,^168^,^173^,^180^,^192^]. These REM sleep abnormalities (especially longer duration, higher frequency, and shorter latency) also manifest in animal models of depression [178,179]. All this evidence corroborates *hypotheses 33, 34, 35, and 39* and, therefore, *hypothesis 20*.

Of course, *vulnerability* is not measured directly. It is inferred here from the neurobiological conditions that depression imposes on the organism. Thus, it is the biochemical dysregulations (e.g., in the serotonergic, noradrenergic, and cholinergic systems) caused by depression that result in a more vulnerable organism. It is these biochemical dysregulations that affect the circuits of the brainstem and other cortical regions (e.g., the amygdala) that regulate REM sleep. Now that I addressed depression, I will discuss stress.

Stress commonly impacts all body systems (e.g., cardiovascular, muscular, endocrine, nervous, respiratory, reproductive, and gastrointestinal systems). Regarding the cardiovascular system, acute stress increases heart rate, dilates the heart, intensifies heart muscle contractions, and reduces blood flow in organs that are not involved in rapid motor activity to redirect it to the large muscles—something particularly relevant in the context of fight or flight [61,64].

Regarding the endocrine system, stress increases the production of hormones that activate the physiological responses to it—one of which is cortisol [61]. When the brain detects a stressful situation—whether recalled or actually present—it triggers a cascade of stress-related hormones that serve the purpose of preparing the body to fight or flee. *This fight-or-flight response constitutes one of the primary survival mechanisms for an organism. Without this mechanism, a predator would be unable to capture its prey, and a prey would be unable to escape from its predator* [52,60,61,64]. In short, the immediate result of stress is to favor, directly or indirectly, the survival of the organism.

Someone might assume that it is incorrect for me to assert that stress makes the organism better protected (or less vulnerable). This person might argue that “as a prey, the stress during a fight-or-flight reaction indicates that I am being hunted, which is equivalent to saying that I am vulnerable.” Thinking this way is incorrect. The vulnerability is due to the predator, not the stress. It is the stress that allows a prey to have some chance of successfully escaping from a predator. Without stress (and the other components of the fight-or-flight reaction), this would be impossible [52,60,61,62,64]. Stress automatically provides a prey with an internal state whose purpose is to enable behavioral responses appropriate to the context of fleeing or fighting (e.g., increased heart rate, increased blood pressure, and increased blood flow directed to the arteries of large muscles), thereby increasing its chances of survival. Therefore, what is truly incorrect is to assert that stress does not contribute to reducing the organism's vulnerability.

Considering that stress (due to the physiological state that favors survival) reduces the organism's vulnerability, this implies that any organism under the influence of stress hormones will have its REM sleep affected. According to the Sentinel Sleep Theory, stress should cause the brain to invest less time in REM sleep, increase the latency to the first REM episode, and increase the density of REM sleep. It may also cause the first REM episode to be (albeit subtly) shorter than the others. After all, given that N-REM sleep is highly important and that it predominates at the beginning of sleep [76], with the organism being better protected, the brain can dedicate less time to REM sleep and more time to N-REM sleep.

The reason it is expected that REM sleep density increases (rather than decreases) under the influence of stress is that stress leaves the organism prepared for a fight-or-flight response. This makes REM sleep more intense. Putting it another way, stress makes the organism more easily awakened during REM sleep because, among other effects, stress reduces the organism's vulnerability by increasing vigilance [60,193]. Therefore, considering that both REM sleep and stress reduce the organism's vulnerability by increasing vigilance, the combination of both results in greater intensity of REM sleep. *Just as combined vulnerabilities produce more intense effects on REM sleep parameters, combined protections also do the same*.

Feinberg and colleagues [188] already proposed that the density (or intensity) of REM sleep may be related to the level of arousal. Some data support this hypothesis [187]. Here I assert, based on the Sentinel Sleep Theory, that REM sleep density is indeed directly related to arousal (or alertness, or vigilance, or attention). *My argument is that REM sleep density is proportional to the level of alertness*. In other words, REM sleep density is a measure of the organism's level of alertness. This implies that the organism will awaken if REM sleep density reaches a very high intensity (i.e., a threshold). Therefore, it is not surprising that nocturnal awakenings are frequent in patients with Major Depressive Disorder (MDD) [167,180,[273], [274], [275], [276]]. (Remember that depression is associated with a higher density of REM sleep.) I will present henceforth some additional evidence that corroborates what I stated in this and the two preceding paragraphs.

When N-REM sleep predominates, cortisol levels reach their minimum; when REM sleep predominates, cortisol levels increase, approaching the cortisol levels associated with alertness during wakefulness—the peak is reached when the organism awakens [76]. In the study by Feng and colleagues [182], the REM sleep density in participants underwent a significant increase after being subjected to stressful situations; moreover, they were more likely to spontaneously awaken during sleep when under stress. In the research by Rodenbeck and Hajak [190], the authors demonstrated that the number of spontaneous awakenings was correlated with cortisol levels. In the study by Barbato and colleagues [186], the authors demonstrated that the propensity for spontaneous awakening is greater in REM sleep when it exhibits a high density. The same was demonstrated (especially in younger individuals) in the study by Ficca and colleagues [129]. In short, *most spontaneous awakenings are preceded by a high density of REM sleep* ([180,187], see Table 1 of this article).

Now that I demonstrated the evidence that corroborates my conclusion that REM sleep density is a measure of the brain's alertness level, I will present henceforth the evidence that corroborates my other assertions regarding the effect of stress on REM sleep.

Mental tension significantly reduces REM sleep time [149]. And acute cortisol administration in humans increases N-REM sleep, suppresses or substantially reduces REM sleep, and increases the latency of the first REM episode [164,181]. In rodents, stress induces a reduction in both N-REM and REM sleep, with the amount of reduction varying according to the type of stress experienced and the duration of exposure to it [183]. In humans, stress reduces both N-REM and REM sleep, increases REM sleep latency, and increases REM sleep density [182,184,185,189].

In the study by Gonnissen and colleagues [277], the researchers analyzed the effects of sleep fragmentation. To do so, they recruited a group of healthy male participants (*n* = 12). Two conditions were compared: (1) a day without sleep fragmentation and (2) a day with sleep fragmentation. In the non-fragmented sleep condition, the average REM sleep time was 83.5 min, while in the fragmented sleep condition it was 69.4 min: a statistically significant reduction (*p* < 0.05). There was no statistical significance between conditions regarding N-REM sleep latency, wake time, total sleep time, and total time in stage N1. The total sleep time did not change significantly because the reduced REM sleep time was equivalent to the increased time in stage N2. Something particularly relevant is that nighttime cortisol levels were significantly higher in the fragmented sleep condition compared to the non-fragmented condition. Based on my theory, I assert that—given that sleep fragmentation elevates cortisol levels [190,277]—stress due to fragmentation reduces REM sleep.

However, a bit of caution is necessary. Acute administration of cortisol inhibits or suppresses REM sleep, which is consistent with my theory, but the physiological and endocrine reality of the HPA axis and cortisol itself is more nuanced. In the second half of the rest period, the amount of cortisol reaches a peak that directly relates to the greater amount of REM sleep typical of this second half [[278], [279], [280], [281]]. In sum, *while N-REM sleep is associated with a reduction in cortisol amounts, particularly during the deepest stage of sleep, we observe greater amounts of cortisol in REM sleep relative to N-REM sleep* [282,283].

**Fig. 2:**
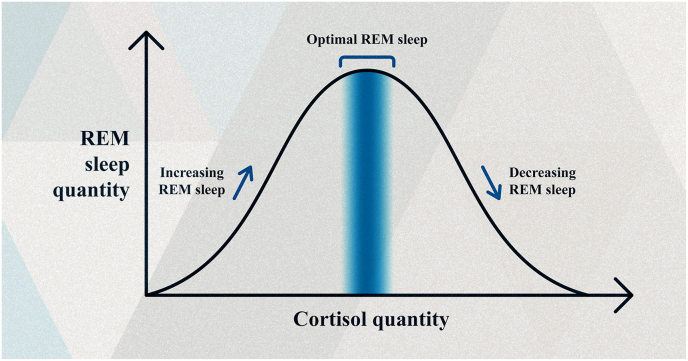
*Curve model of the relationship between REM sleep and the amount of cortisol*. To operate efficiently, REM sleep requires a certain amount of cortisol. After all, cortisol is a crucial component for maintaining the organism protected by modulating attention and vigilance. Therefore, the efficiency of REM sleep as a protective mechanism depends on an appropriate amount of cortisol (the blue bar at the center of the curve indicates the range in which REM sleep operates at its maximum efficiency). However, since REM sleep is modulated by interoceptive information, beyond a certain point the amount of cortisol begins to reduce REM sleep. This occurs because cortisol plays a protective role. This implies that, given the greater protection provided by higher levels of cortisol, REM sleep (which also plays a protective role) becomes less necessary. It is important to remember that REM sleep is energetically costly [4,6] and, for this reason, it is essential that it adjusts when the organism is already better protected due to some other defense mechanism—such as cortisol, for example.

At first glance, these facts appear to contradict the predictions of my theory if we think of a linear model in which REM sleep decreases proportionally to the amount of cortisol. If that were the case, increasing cortisol would progressively reduce REM sleep. However, this linear model is incorrect. The appropriate model is a curve, since cortisol rises alongside REM sleep up to a certain point. In other words, *REM sleep requires a certain amount of cortisol to operate at optimal efficiency*. It is worth recalling that cortisol plays a critical role in safeguarding the organism [52,60,61,62,64], It is only beyond a certain threshold that cortisol begins to inhibit REM sleep, at which point the curve representing REM sleep starts to decline (see Fig. 2). If cortisol levels increase further, REM sleep will eventually be effectively suppressed. Therefore, the evidence above does not refute the predictions of my theory; we just need to analyze the issue with the appropriate caution.

The study by Schmid and colleagues [164] is interesting because the researchers attempted to replicate—in depressed participants—the widely reported suppression of REM sleep as a result of acute cortisol administration. As the researchers stated, they were unable to do so. The reason, from the perspective of Sentinel Sleep Theory, is simple. Considering that this sample included depressed participants, the presence of this disorder prevented the suppression of REM sleep. *Depression, due to the vulnerability it imposes on the organism, prevents REM sleep from being suppressed, even under acute cortisol administration*.

In major depression, the HPA axis becomes overactive [284,285]. Therefore, it would be superficially expected that REM sleep duration would decrease and its latency would increase. However, we must remember that the body is more vulnerable during depression (and it seems precisely for this reason that the HPA axis must become hyperactive, given its role in bodily protection via stress). Consequently—given the heightened vulnerability associated with depression—REM sleep must last longer and have a shorter latency. In other words, vulnerability due to depression takes precedence as information for determining REM sleep parameters, since it compromises the organism's safety; a consequence that, in nature, directly endangers its life. But this does not imply that the HPA axis does not affect REM sleep in depressed patients. In these individuals, since the HPA axis becomes overactive, it is possible that the interoceptive information from the increased circulation of stress hormones contributes to raising REM sleep density.

It is possible to better understand how the HPA axis affects REM sleep by considering that REM sleep requires a certain amount of stress to operate properly (remember that stress serves a protective role). This implies that, within certain limits, increasing the amount of stress helps make REM sleep more efficient as a protective mechanism. REM sleep becomes less efficient—under the influence of stress—only once stress levels begin to interfere with the organism's sleep, that is, when stress is high enough to awaken the organism. Stress can inhibit REM sleep (in non-depressed individuals) only at higher doses, because the brain interprets that the body is already sufficiently protected, making REM sleep less necessary (resulting in shorter duration and longer latency). Moreover, as we have seen, even higher doses of cortisol can actually suppress REM sleep in non-depressed individuals, as it becomes dispensable given the sufficient bodily protection afforded by extremely high amounts of stress.

All the aforementioned evidence regarding stress corroborates *hypotheses 36, 37, 38, 40, and 41*. Therefore, it also corroborates *hypothesis 20*. In summary, *the time invested in REM sleep is inversely proportional to muscle strength and body weight, but directly proportional to vulnerability*. Increasing vulnerability causes the brain to invest more time in the REM period, reduce the latency to the first REM episode, and increase REM sleep intensity; reducing vulnerability causes the brain to invest less time in the REM period, increase the latency to the first REM episode, and reduce REM sleep intensity (except when the organism is under the influence of stress hormones).

Whenever the organism is less vulnerable, REM sleep is significantly reduced, allowing the brain to dedicate more time to the essential N-REM sleep. This is why a reduction in total REM sleep time may, in some cases, be accompanied by an increase in total N-REM sleep time. However, a marked reduction in REM sleep may also be accompanied by no change in N-REM sleep time. In general, a reduction in REM sleep is accompanied by a reduction in total sleep time. After all, reducing REM sleep time naturally affects total sleep duration.

To further corroborate the arguments I developed for the sentinel function of REM sleep, I will analyze REM sleep in neonates. As described by Ribeiro [76], *the amount of REM sleep is strongly correlated with physical immaturity at birth*. Animals that exhibit high autonomy shortly after birth (e.g., sheep, guinea pigs, and giraffes) have a lower amount of REM sleep: about 1 h per day. On the other hand, mammals that are physically immature at birth (e.g., platypuses and humans) show abundant REM sleep at birth, especially in the early stages of life [76]. A newborn human is incapable of moving, feeding, defending, or cleaning itself. Similarly, a baby platypus is also unable to perform these actions and cannot regulate its own temperature without needing to establish physical contact with its mother [76]. The high physical immaturity (or fragility, or vulnerability) with which countless organisms begin life represents a clear disadvantage, requiring regular parental care [76].

Thus, it is not surprising that high neonatal vulnerability is correlated with a large amount of REM sleep [19,76,196,246] Newborn humans sleep an average of 16 to 18 h a day, and about 50% (or more) of this time is spent in REM sleep. In prematurely born babies (who sleep even more), REM sleep time is much more predominant, occurring in about 80% of total sleep time [19,198,246]. In addition to humans, scientists also identified a substantially large amount of REM sleep in neonates of numerous species: in chimpanzees [194], nemestrina monkeys [200], rats [195,197], cats [199], guinea pigs [199], lambs [201,202], and in ferrets [203].

Analyzing ocular activity in fetuses provides another corroboration for my arguments, so I will dedicate this paragraph to that. The density of ocular movements is a way to assess REM sleep activity [286]. Fetal ocular movements consolidate from 23 weeks of gestation, allowing scientists to observe the rapid eye movements typical of REM sleep [206,287]. Fetal rapid eye movements potentially denote the existence of REM sleep, although this is inconclusive [206]. Despite this limitation, given the possibility that these movements indicate the presence of REM sleep, it is interesting to analyze the results of the study by Okawa and colleagues [206]. In this study, the researchers analyzed, in real-time and over 60 min, eye movements in fetuses with a gestational age between 24 and 39 weeks. The results revealed that the period of rapid eye movements was much longer than the period without rapid eye movements. In other studies, scientists showed that between 28 and 30 weeks of gestation, the fetus spends most of its time in REM sleep, with subtle signs of N-REM state [204,205,207]. *As gestation progresses, REM sleep time is progressively reduced, from 80% (at 30 weeks) to 67% (between 33 and 35 weeks) and then to 58% (between 36 and 38 weeks)* [196].

One might attempt to refute my arguments by referencing comparative studies across species that suggest that a potential function of REM sleep is to promote neonatal brain development (e.g., Ref. [[288], [289], [290], [291], [292]]). This would explain the vast amount of time spent in REM sleep in neonates. However, as pointed out by Capellini and colleagues [48], the aforementioned studies have two major flaws. The first is that the authors did not account for the similarity among the species studied due to their common ancestry, an omission that can lead to erroneous conclusions [[293], [294], [295]]. The second flaw is that the comparability of the data has been repeatedly questioned (e.g., Ref. [296,297]; and again by Ref. [48]).

The research by Capellini and colleagues [48] is important to my discussion because the authors did not find support for the hypothesis that one function of REM sleep is to promote neonatal brain development. According to the comparative evidence across species in their study, the need for REM sleep was not significantly greater in species with lower neonatal brain mass, even after adjusting for allometry. What makes Capellini and colleagues’ study robust is that the scientists considered the similarity among the species studied due to their common ancestry. Moreover, they also relied on high-quality data, taking into account the shortcomings of the aforementioned studies. Capellini and colleagues reported that both N-REM and REM sleep showed significant negative correlations with neonatal body mass and with gestation duration, demonstrating that REM sleep does not promote neonatal brain development. In summary, even after controlling the laboratory conditions and phylogeny, the results of their study *did not* support the hypothesis that REM sleep serves to promote neonatal brain development.

All the evidence I presented above corroborates *hypotheses 42 and 45* and, therefore, *hypothesis 20*. Consequently, the correlation between an excessive amount of REM sleep and greater physical immaturity at birth constitutes a corroboration of the Sentinel Sleep Theory. This is exactly what it predicts. After all, since the time the brain dedicates to REM sleep depends directly on the organism's vulnerability, it is predicted that physically immature newborns have abundant REM sleep compared to more physically mature newborns—being more abundant in premature births and even more so in fetuses with greater physical immaturity. In the context of greater physical immaturity, especially in premature cases, neural information from proprioceptive mappings seems to be particularly relevant to determine the parameters of REM sleep (e.g., its duration).

Moreover, it is possible that the variable “vulnerability” encompasses not only external risk (primarily predation) but also internal risk arising from the developmental process itself. After all, development is highly sensitive and vulnerable [298,299]. This perspective suggests that REM sleep parameters (especially its quantity) are also finely tuned according to developmental needs, both of the brain and of the body as a whole. This applies particularly to species whose postnatal development is more extensive, which may explain why certain species exhibit much more abundant postnatal REM sleep.

It is important to note that the sentinel function of REM sleep can be fully executed only when the organism has reached a mature physical development. When, due to some danger, an organism with sufficient physical maturity is awakened from REM sleep, it is fully capable of defending itself (or the group, or its offspring) with all the vigor that waking up during this period enables. In contrast, many neonates are incapable of such a defensive response. The protective function of REM sleep cannot be accompanied by an appropriate defensive behavioral response at this early (and highly vulnerable) stage of ontogenetic development. *REM sleep is a sentinel mechanism that provides greater protection to the organism during the highly vulnerable N-REM sleep, but this protection can only be effectively achieved if the organism is capable (given the appropriate physical maturity) of fighting or fleeing*.

To conclude this Section, I will discuss some factors that increase the robustness of an argument whose arguer relies on a correlation to conclude causality. Although insufficient on its own, what usually serves as evidence to support an assertion concerning a cause is a correlation between two events [300,301]. When one aims to demonstrate that *A* causes *B*, one also aims to demonstrate that it makes sense for *A* to cause *B*. The better the connection (or explanation) established between the cause and the effect, the stronger the argument will be [301].

Moreover, an arguer who aims to establish that *A* causes *B* will increase the robustness of the argument by demonstrating that the causal direction goes from *A* to *B*, but not from *B* to *A*. After all, a correlation does not indicate a direction of causality (when it exists) [300,301,302]. If the causality from *B* to *A* is as plausible as from *A* to *B*, then it will be impossible to determine a unique causal direction; in this case, it may be that both are causing each other [301,302]. Therefore, clearly demonstrating the implausibility of going from *B* to *A* strengthens the argument for a causal direction from *A* to *B*. This is what I will attempt to do to demonstrate that increasing bodily protection causes specific and predictable changes in REM sleep parameters. I will focus on three of the causal arguments I developed.

1. ***Correlation between greater physical strength and less REM sleep time.*** What is causing what here? The causal direction is clearly not from REM sleep to greater physical strength. Who would argue that having less REM sleep causes greater muscle strength? Patients medicated with antidepressants experience a total (or near-total) suppression of REM sleep [19,76,134], but they do not develop the muscles typical of high-performance athletes. Clearly, it is not the REM sleep that causes greater muscle strength. It is the greater muscle strength that causes specific changes in REM sleep parameters (e.g., the time invested in it). This reinforces my argument that the direction of causality is from greater muscle strength to REM sleep parameters.
2. ***Correlation between greater body mass and changes in REM sleep parameters.*** In obese individuals, the changes in REM sleep parameters are analogous to those of high-performance athletes: less REM sleep time and greater latency to the first REM episode. If these changes were responsible for causing obesity, high-performance athletes would constantly be at the mercy of persistent obesity. The alteration of these parameters does not cause obesity. It is obesity that alters these parameters. This reinforces my argument that the direction of causality is from obesity to REM sleep parameters.
3. ***Correlation between depression and changes in REM sleep parameters.*** Most humans have REM sleep. Rare are the people who do not [303]. For practical purposes, it is convenient to simplify: virtually all humans have REM sleep. However, not all have depression. In 2023, an estimate presented on the *World Health Organization* (WHO) website pointed to an incidence of depression in about 3.8% of the world's population [304]. The causal direction in this case is clearly not from REM sleep to depression. The alteration of REM sleep parameters does not cause depression. If it did, we would all have depression. After all, as I showed, emotional states (e.g., fear and stress) are also correlated with changes in REM sleep parameters. We all experienced these emotions, but not all of us developed depression. It is not the REM sleep that causes depression. It is the depression that causes specific changes in REM sleep parameters. This reinforces my argument that the direction of causality is from depression to REM sleep parameters.

Another important factor is the complexity of causal relationships. Many causes possess a complex chain of causal relations in series. Failing to consider this is an error. It may be that *A* causes *C*, but that this causal relationship occurs due to a third causal factor, *B*, operating between factors *A* and *C*. In this case, it would be more accurate to say that *A* causes *C* indirectly [302]:

A⟶B⟶C

When a causal relationship possesses a causality structure in series, it can be described as complex [302]. And that is precisely what is happening in the causal relationships I addressed in this Section. It is not physical exercise (or depression, or stress, *et cetera*) that directly causes changes in REM sleep parameters. Physical exercise increases muscle strength, which in turn reduces the organism's physical vulnerability. And it is this reduction in vulnerability (or its increase in other cases) that causes specific changes in REM sleep parameters. Vulnerability (or the organism's level of protection) is the intermediate causal factor in this complex chain of causal relationships in series.

To reinforce my causal arguments, it remains for me to analyze the possibility of a common cause that could explain the correlations I discussed. I will focus on the correlation between greater physical strength and less REM sleep time. Someone might argue that greater physical exertion requires more restorative processes dependent on N-REM sleep, thus costing the time available for REM sleep. Is this plausible? It is a possibility. However, this argument is a double-edged sword: it can be used both to refute and to corroborate my arguments.

According to this line of reasoning, exerting more effort reduces REM sleep due to restorative processes dependent on N-REM sleep and exerting less effort increases REM sleep by requiring less of these processes. It turns out that sedentary behavior in non-obese individuals is correlated with more REM sleep time and shorter REM sleep latency [165,174]. And this is something predicted from the Sentinel Sleep Theory. After all, non-obese sedentary individuals lack both the greater muscle strength of more active individuals and the higher body fat of obese individuals. The most reasonable conclusion is that the greater physical vulnerability of non-obese sedentary individuals is the relevant causal factor here. Sedentary behavior reduces muscle strength, and this, in turn, makes the organism more vulnerable than its more active peers, requiring more REM sleep and a shorter latency to the first REM episode.

Moreover, in the study by Kitamura and colleagues [152], which I discussed at the beginning of this Section, the following variables *were not significant* in the comparison between groups: age difference (*p* = 0.860), time of physical exertion (*p* = 0.579), and BMI (*p* = 0.920). One variable that *was significant* between the groups is precisely the difference in muscle mass (*p* < 0.001). With this, I aim to demonstrate that since the time of physical exertion (and other variables) did not show a significant difference between the groups, what explains the difference in REM sleep parameters is precisely the difference in muscle mass. Therefore, the double-edged sword proves much more favorable to one interpretation than the other.

The necessary criteria to establish a causal relationship are: (1) it is true that the cause occurred; (2) it is true that the effect occurred; (3) the cause precedes the effect (specific temporal relationship); (4) considering the stipulated conditions, it is practically impossible for the cause to occur and the effect not to occur; (5) the cause plays a crucial role (if the cause does not occur, the effect also does not occur); (6) there is no common cause to explain the cause and effect; [300]. The causal arguments I developed based on correlational evidence meet these criteria and, therefore, constitute solid arguments.

An arguer who relies on a correlation to conclude causality (*post hoc* argument) makes the argument fallacious only when the sole evidence used to corroborate it is the correlation itself [302]. This is why correlation alone is incapable of conclusively establishing causality [300,301,302]. The arguments I developed in this Section to demonstrate causality are not based solely on correlation itself. I showed that the number of correlations is too large to be considered a coincidence. I provided a robust explanation that connects the causes to the effects. I demonstrated the implausibility of the causal direction occurring in the opposite sense in three of the cases analyzed. I demonstrated that there is no common cause to explain the correlations. And finally, I demonstrated that there is a complex chain of causal connections in a temporal sequence. *Therefore, I provided sufficient evidence to conclude causality*.

Even if there are some residual flaws in the causal arguments I developed, they do not compromise them, nor do they turn them into *post hoc* fallacies. Any critical questioning that may arise from my causal arguments will only indicate the need to carry out empirical tests aimed at refuting this causality. This is precisely one of my objectives with this manuscript.

### 4.5 REM sleep probably evolved from a brief awakening from N-REM sleep

Addressing the origin and evolution of REM sleep will allow me to demonstrate that the theory I developed here makes evolutionary sense—a necessity for any arguer aiming to explain the biological function of a trait. This is why this Section exists.

For obvious reasons, we (scientists) are incapable of knowing with certainty how the behavioral state we happen to call “REM sleep” first emerged in evolutionary history. Some of its facts will inevitably continue to escape us. They will remain forever as objects of speculation—no matter how well-founded these speculations may be. There are mainly four pieces of information about primeval REM sleep that we can never know factually: (1) how many genes were responsible for engendering this behavioral state the first time it emerged in an organism, (2) the number of REM episodes in that primeval occurrence, (3) its latency, and (4) its intensity (or density).

Up to this point, I described the evolutionary origin of REM sleep as if it were due to the action of only one specific gene. This may certainly have been the case. However, it could also have been based on the joint action of two or more genes. Scientists have long known that the formation of a phenotypic trait often involves the influence of more than one gene—what is termed *polygeny* [44,305]. When a gene has a phenotypic effect, this effect (in the vast majority of cases) is not due to the gene *per se* because phenotypic traits are often engendered through the action of multiple genes [44,305]. Therefore, the onset of REM sleep could have been based on the action of more than one gene rather than just a single one. The definitive answer to this question, however, we will never know for sure.

I am unable to empirically analyze REM sleep in its primeval occurrence, but I can develop *a priori* arguments about its initial complexity and the number of episodes. This is what I will present hereinafter in the form of a historical narrative.

Because we deal with past events (e.g., the origin of a new trait), we, evolutionary biologists, are unable to empirically test our object of study. Evolutionary phenomena are inaccessible to experimental methods. Thus, to obtain answers to evolutionary questions, we must resort to a non-experimental method called *historical narratives* [40]. This method is based on the formulation of a narrative about past events, primarily supported by their consequences, and whose explanatory value must be tested. To do so, one must rely on any evidence that can refute or corroborate the predictions generated from the historical narrative [40].

Before presenting the historical narrative I developed to explain the evolutionary origin of REM sleep and its subsequent evolution, I will introduce certain crucial concepts that will serve to ground the proposed narrative. These will be used as a more secure starting point on which to base my speculations. By ensuring a solid foundation, I hope that the proposed narrative is not far from the truth.

As determined by the first law of probability, *the probability of two events occurring together is never greater than the probability of each event occurring separately* [[306], [307], [308], [309]]. Putting it another way, the coincidence (i.e., the joint incidence) of two or more events implies multiplied probability [43,306].

The first law of probability is closely related to the mathematical concept of complexity, according to which complexity constitutes a statistical concept [42,43,310,311,312]. Under this sense, complexity is *a priori* associated with high statistical improbability, being inversely proportional to its probability of occurring. The greater the complexity of something, the lower its probability of occurring, and vice versa [310,311,313]. Complex (or statistically improbable) things do not arise suddenly. To be achieved, complexity—especially in the biological context—requires a countless number of sufficiently simple intermediate steps [42,43].

Biological complexity is distinguished from inorganic complexity (which is comparatively more limited) due to the attribute of *functionality* (e.g., walking, running, flying, swimming, or digging). In general, the functionality that defines the high biological complexity encompasses all mechanisms directly or indirectly responsible for the conservation of life (due to the maintenance of a chemical balance favorable to it) and for reproduction. In addition to these, also included are the mechanisms that allow the organism the ability to find energy and process it, to replace all aging subcomponents that die, and to defend itself from physical injuries and diseases [43,52,63,78].

The high statistical improbability manifested by living beings emerges in the world as a product of a long series of intermediate evolutionary steps that are simple enough (compared to the previous steps) to occur by chance—being, therefore, functionally random [42,43].

Everything I discussed in the preceding paragraphs is particularly relevant to the *a priori* arguments about the primeval occurrence of REM sleep that I will develop hereinafter. But before introducing and explaining the historical narrative, I will first provide a brief overview of the current complexity of REM sleep.

REM sleep is generated by the coordinated action of various neurotransmitter systems in the brainstem, forebrain, and hypothalamus, and by the activation of several brain regions (e.g., amygdala, hippocampus, motor cortex, cingulate cortex [especially the anterior region], brainstem, thalamus, and visual association cortex); it includes intense muscle atonia; and it is based (in humans) on an amount of four to six REM episodes throughout the entire rest period [3,5,56,105,314,315,316]. Muscle tone is present during N-REM sleep but is low and does not compare to the intense muscle atonia associated with REM sleep, which practically paralyzes the body. With few exceptions, most of the body remains incapable of movement during REM sleep. The muscles involved in breathing move, but more mildly. Meanwhile, the muscles that control eye movements, as well as the muscles of the inner ear, move intensely [3,5,17].

If REM sleep, at its inception, already involved multiple neural regions, included five alternating episodes, and featured intense muscle atonia, we would be dealing with a highly complex mechanism, based on a series of independent events cooperating for the same purpose: reducing the organism's vulnerability. All this complexity would naturally require efficient coordination between all neural regions involved in this primeval occurrence of REM sleep. However, the probability of all these independent events occurring together is negligible. Therefore, the primeval occurrence of REM sleep was—in all likelihood—not like this.

The scenario of a highly complex primeval REM sleep is equivalent to a huge stroke of luck, a high statistical improbability. After all, the complexity in this case is both structural and behavioral. Therefore, I can assert—with the confidence derived from statistics—that the primeval REM sleep was not based on a highly improbable event. Its onset, for the sake of plausibility, had to be simple. We cannot postulate a primeval occurrence of REM sleep based on multiple independent events occurring simultaneously, as the probability of this occurring is far lower than the probability of just one of these events occurring.

This leads me to the following questions: *What is the simplest possible scenario for the primeval occurrence of REM sleep? What scenario requires the least statistical improbability?* Considering that the neural mechanisms responsible for regulating N-REM sleep obviously already existed, the most probable scenario (due to its simplicity) is that REM sleep emerged as an error causing a brief awakening from N-REM sleep. (*Note that the term “error” should be understood in the sense of a failure in the control of the transition from N-REM sleep to wakefulness, causing the organism to awaken before the usual time*.) Consequently, this error provided a limited but not non-existent adaptive advantage for the organism. After all, briefly waking up from N-REM sleep—a highly vulnerable state—can contribute to survival. This contribution was limited, but the chances of survival were higher for the organism that briefly woke up from N-REM sleep than for those that remained asleep. The brief awakening allowed for more efficient scanning of the surrounding environment for the presence of any potential dangers. From this, it is easy to see that any subsequent modification (due to a functionally random mutation) that enhanced this function would clearly be favored by non-random elimination. What improvement might have occurred next?

The next evolutionary step was probably the brief awakening turning into an *ease* of awakening. Now, instead of REM sleep fully awakening the organism, the brain enters a state that only facilitates awakening. Thus, the advantage of greater neural activity—to ensure vigilance and readiness to fight or flee—is harnessed without affecting the organism's sleep. The selective pressure for this transformation occurred because the awakening caused by the primeval REM sleep inevitably affected the organism's sleep. Notably, at this stage (of the ease of awakening), the intense muscle atonia (due to the high complexity of this mechanism) was probably still absent. Considering that N-REM sleep already had milder muscle atonia, it is possible that REM sleep had it too. However, due to the high intensity of neural activation, this milder muscle atonia (assuming its presence) was probably unable to prevent the organism from moving during REM sleep.

This seems like a problem for my historical narrative. After all, it is obvious that an organism moving while asleep attracts predators or even competitors from its own species [76]. However, we must ask the following. Who is more vulnerable: an immobile organism while in N-REM sleep (with low levels of attention, vigilance, and readiness, especially in deep sleep), or a sleepwalking organism during REM sleep (with high levels of attention, vigilance, and readiness)? Which one is better prepared to fight or flee? The answer is self-evident.

The next evolutionary step was probably the occurrence of more than one period of REM sleep. Now, instead of just one, the organism had more than one REM episode (probably two, but there could have been more). The addition of one or more REM episodes provided a more considerable adaptive advantage than the previous version, with just one episode during the entire rest period. After all, with more than one sentinel period during the so-vulnerable N-REM sleep, the organism's brain had more opportunities to effectively scan the surrounding environment. At this stage, the intense muscle atonia was probably also absent.

It is at this point in the narrative that selective pressure for the development of intense atonia of the striated muscles intensified. The presence of more than one REM episode—especially when this number exceeded two—created growing pressure to develop a mechanism capable of significantly *reducing* the movements of striated muscles during REM sleep. The development of this mechanism was probably the next evolutionary step. Subsequently, this mechanism became more complex to the point of effectively *paralyzing* striated muscle movements during REM sleep. (The temporary paralysis of muscle movements certainly came after their reduction. After all, a mechanism to reduce striated muscle movements is less complex [or statistically more probable] than a mechanism to paralyze them. Furthermore, N-REM sleep already had mild atonia, which probably served as the basis for the mechanism of striated muscle paralysis in REM sleep.)

In this historical narrative I proposed, each step is a small modification that confers a selective advantage over the previous state. Small enough to be likely to arise from a *de novo* mutation (a mutation in an individual's DNA sequence that was not inherited from its parents). Although we cannot know for sure, the *a priori* arguments I developed here should generally not be far from the truth concerning the primeval REM sleep and its subsequent evolution over countless generations. Obviously, no fossil is (or will be) able to corroborate these claims; fossils do not contain records of sleep [76,208]. One fact about the primeval occurrence of REM sleep is the certainty that we will never know for sure how it began. The primeval REM sleep and its subsequent evolution will retain some secrets. The factual details of the evolutionary origin of both N-REM and REM sleep, as well as the origin of this separation, will remain *in perpetuum* as objects of speculation. What we must ensure (for as reliable an understanding as possible) is that these speculations are well-founded. This is what I hope to have achieved with the historical narrative I developed in this Section.

## 5 Conclusion

The primary function of REM sleep is to reduce the vulnerability caused by N-REM sleep. (Let this statement not be interpreted as if I were claiming that secondary functions do not exist. This is a separate issue and is beyond the scope of an already overlong article.) I explained why and how REM sleep emerged, why and how it became more complex throughout the evolution of species, and why and how REM sleep parameters depend on factors associated with body protection or vulnerability. Considering all the strategies I adopted to select the references and mitigate any biases (see Section 2), it is safe to assert that the assertions I concluded from the data I analyzed are epistemically justified and possess methodological integrity.

A critical characteristic of a good scientific theory is its empirical testability. Thus, the greater the amount of empirical information a theory gathers (i.e., its *empirical content*), the greater the number of possibilities for falsification it contains [32]. The theory I am proposing here contains a vast empirical content, contributing to its robustness, given the numerous ways in which we can test it.

To test the Sentinel Sleep Theory, I drew on a substantial body of evidence and testable hypotheses. After testing the hypotheses and analyzing the evidence, I concluded that they corroborate the Sentinel Sleep Theory. Furthermore, I showed that numerous attempts to refute it failed. Finally, something even more important: I listed 51 specific hypotheses derived from my theory, of which 39 are empirically testable, which allows other scientists to exhaustively test the theory I proposed here—especially the hypotheses in Table 2 for which there is little or no research. This way, we can not only further corroborate it, but also refine it or remove any flaws that I may have been unable to notice or resolve.

The question “Does REM sleep serve the same purpose across different animal lineages?” remains an open problem [6]. However, based on my article, the answer to it becomes manifest. The *primary* function of REM sleep is the same for all organisms that possess this behavioral state. For any organism with a nervous system, supplanting sleep is (apparently) impossible. However, this is not the only way to reduce its high vulnerability. REM sleep solves this problem. *REM sleep is a necessary adaptation for every organism with a nervous system that, therefore, needs to sleep*. A mechanism like REM sleep—given its high biological relevance—would certainly become a priority and imperative in the course of biological evolution; it would inevitably spread widely among animals. And that is exactly what happened.

Since the functionally random genetic mutation that engendered the primeval occurrence of what we now describe as “REM sleep,” non-random elimination ensured the widespread dissemination and persistence of this mechanism responsible for providing greater defense to the organism during the vulnerable N-REM sleep. REM sleep provided a substantial adaptive advantage to its bearers, as it compensates for the high vulnerability to which organisms are subjected during N-REM sleep. For this reason, the sentinel function of REM sleep has not only been conserved throughout evolution but has also undergone remarkable complexification, achieving a high efficiency as a protective mechanism. The biological importance of this mechanism is such that it may even have evolved independently.

It is therefore no surprise that the brainstem—together with other cortical regions such as the amygdala and the hypothalamus—is responsible for generating REM sleep [315,[317], [318], [319], [320]]. After all, this extension of the spinal cord encloses structures that control numerous *survival-related functions*, such as heart rate, respiration, orgasm, swallowing—and, of course, sleep [[321], [322], [323]]. And, as I am arguing, *REM sleep is a basic mechanism directly associated with survival*. The fact that the amygdala and the hypothalamus are also regions that generate REM sleep [318,319] is consistent with my argument that REM sleep plays a direct role in the survival of the organism. After all, both the amygdala and the hypothalamus are crucial neural regions in the physiological fight-or-flight response [61].

REM sleep is regulated directly by information provided by all types of neural mappings: interoceptive (e.g., stress due to the presence of cortisol in the bloodstream), proprioceptive (e.g., muscle strength), and exteroceptive (e.g., exposure to an unknown environment). The information from these three varieties of neural mappings determines the parameters of REM sleep: the time invested in it, its latency, the duration of each episode, the number of episodes, and its intensity (or density). In short, REM sleep is a biological mechanism that evolved to depend on any factors directly or indirectly related to protection and vulnerability (e.g., emotions; body weight, muscle strength, and the bilateral occurrence of N-REM sleep). Therefore, *for REM sleep to be more precisely studied henceforward, any factors directly or indirectly related to the organism's protection or vulnerability should be isolated because they are confounding factors*. Failing to separate the confounding factors that affect REM sleep parameters will lead to disparate results among studies. More precisely guiding future scientific investigations of sleep is one of the central contributions of my article.

The three main reasons for a scientific theory to be accepted as valid and robust are (1) the corroboration it has, (2) the number of attempts that failed to refute it, and (3) its ability to generate testable hypotheses. A theory encompasses hypotheses, facts, and laws (when applicable) to explain a multitude of previously collected evidence and to propose a series of specific predictions about future events—a crucial characteristic of a good scientific theory [42,56,324,325]. What makes a scientific theory good is much more its ability to generate testable hypotheses than its empirical foundation [56]. The more testable hypotheses a theory encompasses in its conceptual body, the better it is. Furthermore, what makes a theory even better is its ability to solve *significant* conceptual and empirical problems [35].

The Sentinel Sleep Theory passes these tests. Throughout this article, I presented an extensive factual basis that solidly supports and corroborates the arguments I developed to demonstrate that the primary function of REM sleep is to act as a sentinel mechanism. Through the Sentinel Sleep Theory, it is possible to accurately explain a substantial amount of disparate facts related to REM sleep; facts that come from numerous animals (e.g., zebrafish, cuttlefish, octopuses, drosophila, reptiles, nemestrina monkeys, chimpanzees, humans, rats, mice, birds, sheep, giraffes, cats, guinea pigs, lambs, ferrets, dolphins, belugas, orcas, porpoises, whales, and fur seals).

The arguments I developed to integrate the conceptual framework of the Sentinel Sleep Theory are consistent with biological, embryological, homologous, phylogenetic, genetic, evolutionary, physiological, neurophysiological, endocrinological, immunological, neurobiological, neurochemical, neuropharmacological, ontogenetic, allometric, and mathematical and statistical evidence. Additionally, numerous attempts to refute it failed (See Section “S1” of Appendix A). Many pieces of evidence that seemed to offer some refutation ended up revealing corroboration under scrutiny. (A lesson that must be considered in future research.) Given the 452 references I discussed here (most of which I used as empirical support), the evidence strongly suggests that I presented the central biological function of REM sleep. The numerous pieces of empirical evidence I gathered to corroborate the Sentinel Sleep Theory and the robust arguments I developed to demonstrate how to explain such evidence are strong enough to ensure that no single article is capable of disproving it. Especially because the quality of the data matters.

In light of all the arguments I developed to compose the conceptual framework of the Sentinel Sleep Theory, the numerous attempts that failed to refute it, the 38 factually confirmed hypotheses, and the 452 references I discussed here, it seems appropriate to state that *the Sentinel Sleep Theory offers the most comprehensive and well-founded explanation to date for the biological function of REM sleep*. No other theory can so robustly explain an enormous number of disparate facts pertaining to the domain of REM sleep, including its origin and subsequent evolution. I do not have space to demonstrate the flaws of every hypothesis already proposed to explain the function of REM sleep, but I can remind the reader what they are: *hypotheses*.

For more details on how my theory solves a greater number of empirical problems compared to rival hypotheses, see Section “S6” of Appendix A. For a list of ways to refute my theory, see Section “S7” of Appendix A.

The purpose of theories is to unweave reality, allowing us to understand a given phenomenon more accurately. That is why one of the most important marks of a good theory is its ability to solve problems. Consequently, the best theories are those that untangle the often-chaotic web of empirical facts that enmesh a scientific domain. With my theory, I collected a highly tangled assemblage of numerous disparate facts and explained them in a simple, clear, and precise manner. The arguments and evidence I presented here lead me to the rational belief that the Sentinel Sleep Theory was able to unweave the vast and chaotic web of facts that once entangled the domain of REM sleep.

## Declaration of competing interest

I have nothing to declare.
