# Supplementary material for: The Sentinel Sleep Theory: Unweaving the biological function of REM sleep
Source: Sleep Med X. 2026 Apr 9;11:100186. doi: 10.1016/j.sleepx.2026.100186 (PMC13208913; doi:10.1016/j.sleepx.2026.100186)
Supplement: Multimedia component 1 [file mmc1.docx]

**Appendix A. Supplementary discussion**

**S1. Attempts at refutation**

***1. If N-REM sleep already provides a certain quantity of alertness and protection during sleep, why is REM sleep necessary?*** First, we must recognize that an organism’s quantity of protection and alertness varies on a spectrum. This means that an organism—depending on its neural state and its physiological and behavioral responses—can be more or less protected; more or less alert. The spectrum of alertness and protection is distributed as follows: during wakefulness, alertness and protection are very high. During N-REM sleep, they are very low. REM sleep occupies a position between these two, lying closer to wakefulness: it is a sleeping wakefulness.

It is easy to understand why N-REM sleep must retain a minimal amount of alertness. After all, having *some* alertness is certainly better than having *none*. However, it can be improved. *Increase alertness a bit more, and the organism will be even better protected*. This is where REM sleep comes in. N-REM sleep does indeed provide some quantity of alertness (Gazzaniga et al., 2016, p. 146; Moyne et al., 2022), but it cannot match the alertness and the physiological and behavioral responses that REM sleep enables (Tseng et al., 2022). Therefore, the fact that N-REM sleep offers some alertness and protection neither precludes the existence of REM sleep nor diminishes its function. Furthermore, the HPA axis—an important protective component (see Section 4.3)—is quietest when N-REM sleep is at its deepest state, something that happens especially during the first half of the rest period (de Feijter et al., 2022; Vgontzas and Chrousos, 2002; Weitzman et al., 1974; Weitzman et al., 1983). That is, N-REM sleep clearly places the organism at a more pronounced vulnerability than REM sleep.

In Section 4.5, I explained why intense muscle atonia probably evolved over the course of biological evolution. However, some doubts may remain regarding the plausibility of my proposal. After all, pronounced muscle atonia during REM sleep can increase the organism’s vulnerability to environmental threats (Lima et al., 2005). Yet we must remember that animals respond more effectively to threats upon waking from REM sleep than from N-REM sleep (Horner et al., 1997b; Tseng et al., 2022; Voss, 2004). Moreover, a recent study indicated that the muscle twitches that occur during REM sleep are capable of causing sensorimotor activity that prepares the organism for wakefulness (Brooks and Peever, 2016). Finally, an even more recent study clearly demonstrated that REM sleep possesses specific characteristics enabling an animal to awaken easily upon detecting a dangerous stimulus (Tseng et al., 2022). These characteristics include rapid and specific reactivity to predatory stimuli, rapid increase in pupil size, and rapid increase in the ability to move when detecting a predatory stimulus. All these characteristics ensure that the animal can efficiently defend itself against threats to its life during REM sleep (Tseng et al., 2022).

***2. Phasic REM can be less responsive than tonic REM*.** Another attempt to refute my theory is to resort to the tonic and phasic periods of REM sleep. REM sleep is not a homogeneous state, comprising at least two phases: tonic and phasic (Simor et al., 2020). Evidence indicates that phasic REM is less responsive to certain environmental stimuli than tonic REM (Atienza et al., 2001; Ermis et al., 2010; Price and Kremen, 1980; Sallinen et al., 1996; Takahara et al., 2002). Given this difference, one might conclude that the Sentinel Sleep Theory does not hold. However, for my theory, it matters little whether phasic REM is less responsive to some environmental stimuli than tonic REM (and understanding why falls outside the scope of this work). What matters for my proposal is *responsiveness to potential threats*, not to any environmental stimulus. Note that *all* of the above studies used auditory stimuli, not dangerous auditory stimuli. *This difference matters*. The studies comparing environmental responsiveness between tonic and phasic REM *did not test the specificity for threatening stimuli*. That is what must be tested in order to corroborate or refute my theory. To overturn my theory, it would be necessary to demonstrate that REM sleep *is not* selectively responsive to dangerous stimuli. Yet the evidence shows that REM sleep is, indeed, selectively responsive to dangerous stimuli (Tseng et al., 2022). What remains is to analyze how this specificity varies (or does not vary) between the phasic and tonic periods.

***3. Powerful species and REM sleep*.** Another (incorrect) attempt to refute my theory is to appeal to the fact that powerful species exhibit REM sleep (Allison and Cicchetti, 1976). If REM sleep serves to protect the organism during sleep, is it necessary for powerful species (such as lions and tigers) to have REM sleep? This is indeed an important question, but we must be cautious about oversimplifications. The idea that predators attack and prey defend is an overly simplistic view (Magalhães et al., 2005). We must remember that no predator is born as an adult, physically mature. Young predators are indeed more vulnerable (Magalhães et al., 2005). Therefore, the notion that predators (or powerful species) should not exhibit REM sleep is an error resulting from this simplification. Predators can also be attacked, especially when they are young (Magalhães et al., 2005).

We must always remember that animals can be both predators and prey. Cats hunt mice, but they are also preyed upon by other animals, such as foxes, coyotes, wolves, large birds of prey (e.g., owls and eagles), and even by other wild cats. Lions—apex predators—are likewise threatened by other animals, including hyenas, buffalo, wild dogs, rhinoceroses, elephants, hippopotamuses, crocodiles, leopards, and even other lions (Kingsley, 2024; Williams, 2025).

For the sake of argument, let us suppose that REM sleep is eliminated in predators. What would happen to their vulnerability during sleep? In contrast to REM sleep, awakening from N-REM sleep (especially from deep sleep) is *not* associated with full alertness or optimal sensory and motor efficiency (Kandel et al., 2013, p. 1157; Lima et al., 2005; Horner et al., 1997b; Ribeiro, 2021; Snyder, 1966; Tseng et al., 2022). Consequently, without REM sleep, predators would become more vulnerable during rest. It would be easier for a physically immature predator to be killed during sleep, either by prey or by another predator. Furthermore, it is possible that the absence of REM sleep would pave the way for a prey species (or more than one) to develop the strategy of attacking their predators while they sleep. After all, without REM sleep, a predator attacked during sleep would awaken completely disoriented and would remain momentarily unable to defend itself with the full vigor that only awakening from REM sleep allows. This strategy could also be developed by other predatory species. It is important to remember that there are numerous reports of predators attacking other predators (Bohannon, 2006).

We must also remember that natural environments are challenging. Predators are strong and generally large (Magalhães et al., 2005), but they are neither invulnerable nor indestructible. Even strong predators can suffer accidents, which increases their vulnerability both during wakefulness and sleep. In addition, predators are not immune to the harmful effects of prolonged hunger, another factor that can place them in a state of greater physical vulnerability. After all, a well-nourished predator has a clearly more advantageous defensive capacity compared to a malnourished predator. (Anyone who watched nature documentaries knows that, on many occasions, predators go without food for long periods.) Therefore, I reiterate: *the idea that strong species should not have REM sleep is incorrect*.

Furthermore, it is possible that *the amount of REM sleep a species exhibits is also a product shaped by that species’ overall life-history strategy*. Apex predators, such as lions, tend to follow a life-history strategy characterized by producing fewer offspring compared to prey species, such as rodents (Burak et al., 2018). This implies that, for a predator, losing even a single offspring carries a greater cost for the species’ survival. Therefore, investing more in REM sleep—even though it is more energetically costly—makes perfect evolutionary sense. By contrast, for prey species, which tend to produce a larger number of offspring throughout their lifetime, losing a single offspring is less costly from the standpoint of species survival. In this case, survival is better ensured through high reproductive rates. That is, for prey species, the cost-efficient evolutionary strategy is to minimize (as much as possible) the total time spent in the vulnerable state of sleep, even if this means less time in the sentinel state (especially compared to predator species).

In sum, the fact that prey species have shorter gestation periods, faster population dynamics, and overall faster life-history strategies (Abrams, 1992; Abrams, 2000; Becks et al., 2012; Cortez and Ellner, 2010; Post and Palkovacs, 2009) may also help explain why predator species exhibit more REM sleep relative to prey species. In other words, what I am arguing is that *the vulnerability REM sleep seeks to counterbalance may not be strictly immediate. Beyond protecting the individual organism, it may also serve to protect offspring or adult pairs of the same species—thereby ensuring, with greater probability, the species’ posterity*. Therefore, if a species produces fewer offspring over its lifetime, has slower population dynamics, and follows a slower life-history strategy, it is evolutionarily advantageous to have more REM sleep.

Below, I will complete the discussion of the amount of REM sleep in predators and prey with an additional argument.

***4. Species more vulnerable to predation have less REM sleep*.** Evidences apparently contrary to the arguments I developed here are provided by the studies of Allison and Cicchetti (1976) and Lesku and colleagues (2006). In these works, the researchers demonstrated that species more vulnerable to predation have less REM sleep time than predator species. However, caution is warranted when interpreting these findings, as the only way for an animal to have significantly more REM sleep time without sacrificing the necessary N-REM sleep is by sleeping longer. And this is something predators can afford to do. This explains why predator species tend to sleep more, whereas prey species tend to sleep less (Allison and Cicchetti, 1976). Furthermore, REM sleep depends on N-REM sleep (Le Bon, 2020). Le Bon and colleagues (2002) found a strong positive correlation between the proportion of N-REM and REM sleep and the frequency of cycles per night: the more REM/N-REM sleep cycles, the greater the ratio between the durations of REM and N-REM sleep.

Therefore, it is naïve to think that, based on the sentinel function, we should necessarily expect prey species to have more REM sleep than predator species. This conclusion is absurd. If you are a prey sleeping in a more vulnerable environment, you cannot afford the luxury of sleeping extensively; that is the luxury of prey species that sleep in safe locations and—most notably—of predator species. The consequence of this is that, even if the function of REM sleep is to act as a protective mechanism, prey species—being forced to sleep for shorter periods—will tend to have significantly less REM sleep than predators. Hence, the fact that prey species have comparatively less REM sleep than predators does not, in any way, invalidate the sentinel function.

***5. REM sleep and the arousal threshold “paradox”*.** Considering the arguments I developed, it might be—superficially—expected that REM sleep would exhibit a lower arousal threshold than N-REM sleep. However, a search through the scientific literature reveals that REM sleep *does not* have a lower arousal threshold than N-REM sleep, and may even be higher (e.g., Ermis et al., 2010; Pilon et al., 2012). Does this constitute a fatal refutation of my arguments? I will demonstrate hereinafter that these facts actually support (rather than refute) my arguments.

In Section 4.5, I argued that at one point in the evolutionary trajectory there was a growing pressure to develop mechanisms to prevent the organism from waking up during REM sleep. If the arousal threshold in REM sleep were lower than that of N-REM sleep, the organism would wake up much more easily, especially considering the high neural activity of several regions associated with alertness and attention (as I demonstrated in Section 4.3). *Therefore, rather than entailing an arousal threshold lower than that of N-REM sleep, it was imperative that REM sleep entail an arousal threshold analogous to or even higher than that of N-REM sleep*.

To provide additional evidence for the assertions of the previous paragraph, I will turn to cholinergic neurons. Cholinergic neurons—responsible for providing the primary source of acetylcholine to the cerebral cortex—are known to help activate the cortex during both wakefulness and REM sleep (Brown et al., 2012; Datta and Siwek, 2002; Deurveilher and Semba, 2011; Watson et al., 2010). One of the effects of acetylcholine is to increase wakefulness (España and Scammell, 2011; Watson et al., 2010). Indeed, the release of acetylcholine during REM sleep in the basal forebrain and pontine reticular formation is significantly greater than during wakefulness (Vazquez and Baghdoyan, 2001; Watson et al., 2010).

What I aim to demonstrate with this evidence is that REM sleep already possesses numerous mechanisms that facilitate awakening, reinforcing my argument that if the arousal threshold during REM sleep were lower, the organism would wake up much more easily, constantly compromising sleep. The sentinel function of REM sleep is to facilitate awakening, but this ease cannot be that high. There must be a limit. Otherwise, almost anything would wake the organism. *The evolution of REM sleep relied on the development of mechanisms that facilitate awakening, but it also involved the co-evolution of mechanisms that prevent this ease of awakening from becoming too easy to the point of disrupting the organism's sleep*.

To better substantiate the preceding assertion, I will discuss serotonin. Electrophysiological, neurochemical, and neuropharmacological evidence indicates that serotonin promotes wakefulness (Brown et al., 2012; Monti and Jantos, 2008). Furthermore, serotonin helps inhibit both REM and N-REM sleep (Boutrel et al., 2002; Horner et al., 1997a; Monti and Jantos, 2008). The high and constant activity of serotonergic neurons during wakefulness contributes to preventing the transition from wakefulness to either REM or N-REM sleep (Boutrel et al., 2002; Brown et al., 2012; Monti and Jantos, 2008). Serotonergic neurons fire less during N-REM sleep and (which is particularly important for my arguments) barely fire at all during REM sleep (Brown et al., 2012; Monti and Jantos, 2008).

Additional evidence comes from orexin neurons, which also play a crucial role in promoting wakefulness (De Luca et al., 2022; Feng et al., 2020; Ito et al., 2023; Mogavero et al., 2023; Ono and Yamanaka, 2017). This role is so crucial that during the transition to wakefulness, orexin neurons fire at an intensive rate (de Lecea and Huerta, 2014; Lee et al., 2005; Mileykovskiy et al., 2005). During wakefulness, orexin neurons are highly active. When directed to target regions, orexin elevates alertness, promotes arousal, and helps sustain the wakefulness state (de Lecea and Huerta, 2014; De Luca et al., 2022; Estabrooke et al., 2001; Ito et al., 2023; Mogavero et al., 2023). However, during REM sleep, orexin release decreases or ceases because orexin neurons reduce their activity or become silent (de Lecea and Huerta, 2014; Estabrooke et al., 2001; Mochizuki et al., 2011; Mogavero et al., 2023).

In addition to orexin neurons contributing to promoting or sustaining wakefulness, they also prevent the expression of both REM and N-REM sleep (De Luca et al., 2022; Estabrooke et al., 2001; Kandel et al., 2021, p. 1097; Mochizuki et al., 2011; Sasaki et al., 2011). To promote arousal, orexin neurons indirectly inhibit sleep by acting on the neurons of the ventrolateral preoptic nucleus (VLPO), a crucial region for initiating and maintaining sleep (De Luca et al., 2022). This is known because acute stimulation of the VLPO induces sleep (De Luca et al., 2022), and because local administration of orexin in the VLPO causes animals to awaken from sleep (Mavanji et al., 2015).

Selective loss of orexin causes the intrusion into wakefulness of typical REM sleep elements, such as paralysis episodes called cataplexy (characterized by the sudden loss of muscle tone) (Dauvilliers et al., 2007; Mochizuki et al., 2011; Ribeiro, 2021). Loss of orexin also causes narcolepsy in rats, humans, and dogs (Dauvilliers et al., 2007; Mileykovskiy et al., 2005; Mochizuki et al., 2011; Sasaki et al., 2011), impairs the maintenance of wakefulness, destabilizes wakefulness and sleep states, and causes fragmented sleep and sudden entry into REM sleep (Dauvilliers et al., 2007; Kandel et al., 2021, p. 1097; Mochizuki et al., 2011; Ribeiro, 2021; Sasaki et al., 2011). As we can see, this abundant evidence solidly supports my arguments for the co-evolution of mechanisms that prevent excessively easy awakening during REM sleep.

In sum, for the sentinel state to be stable and functional, two opposing strategies must co-exist: mechanisms that increase internal vigilance and readiness (e.g., cholinergic and limbic activation) and mechanisms that simultaneously raise the arousal threshold to prevent the state from collapsing into full wakefulness in response to trivial stimuli. This creates a highly specialized state of intense internal alertness coupled with external disconnection from trivial stimuli—a true sleeping sentinel.

***6. The locus coeruleus-norepinephrine system and REM sleep*.** Another attempt to refute my arguments is to appeal to the locus coeruleus (LC). Since the LC is strongly inhibited during REM sleep (Osorio-Forero et al., 2022; Schwartz and Roth, 2008), this fact might seem like an obvious refutation of the Sentinel Sleep Theory. After all, the LC consists of the primary source of a hormone directly associated with stress and arousal: norepinephrine (NE) (Koshmanova et al., 2023; Poe et al., 2020). The locus coeruleus-norepinephrine (LC-NE) system—by increasing the organism's alertness, stress, and arousal—is involved in neurobiological processes that place it as an important component of the fight-or-flight response (España and Scammell, 2011; Osorio-Forero et al., 2022; Ross and Bockstaele, 2021; Yamaguchi et al., 2018).

These facts about the LC-NE system may (superficially, once again) seem like a significant blow to the arguments I am proposing for the protective function of REM sleep. However, a more careful analysis reveals that these facts actually support (rather than refute) the Sentinel Sleep Theory. The reason it is expected that the LC-NE system would be strongly inhibited during REM sleep is that, among other functions, it plays a central role in maintaining wakefulness (Kjaerby et al., 2022; Poe et al., 2020; Watson et al., 2010). Norepinephrine is a hormone known to promote wakefulness (Watson et al., 2010), and it has been widely demonstrated that activating the LC causes the transition from sleep to wakefulness (Carter et al., 2010; Kjaerby et al., 2022; Swift et al., 2018; Yamaguchi et al., 2018). Moreover, the increase in firing frequency in the LC precedes spontaneous awakenings from N-REM sleep (Aston-Jones and Bloom, 1981; Foote et al., 1980; Osorio-Forero et al., 2022; Takahashi et al., 2010). Finally, it has also been shown that mice experience spontaneous awakening from N-REM sleep more frequently when LC neural activity increases (Cardis et al., 2021; Osorio-Forero et al., 2021).

These facts are crucial for understanding why the LC-NE system is almost completely inhibited during REM sleep. They support my argument that there was increasing selective pressure for the development of mechanisms to prevent the organism from waking up too easily during REM sleep. Consequently, any nucleus, region, or brain system directly and crucially involved in the transition from sleep to wakefulness (such as the LC) would have to undergo significant inhibition. *Therefore, even though the LC-NE system is important in the fight-or-flight response—which would make it an ideal candidate to remain highly active during REM sleep—its crucial role in waking the organism ensured its suppression during REM sleep*. If the LC did not play a central role in waking up the organism, this nucleus would certainly be active during REM sleep.

What I hope to have demonstrated is that the arousal threshold of REM sleep and N-REM sleep do not need to differ for my theory to be corroborated. The most important thing, after all, is that REM sleep presents a behavioral response when the organism's brain detects a potentially dangerous stimulus (not just any stimulus; it needs to be a stimulus that is potentially dangerous to that organism). Tseng and colleagues (2022) demonstrated that this behavioral response occurs. Therefore, the theory remains corroborated. *What I am arguing is that REM sleep is biased toward potentially dangerous stimuli—not just* any *external stimuli*. So it is naive to point to a study like Ermis and colleagues (2010) and claim that my theory has been disproved.

This explanation—about the silencing of key wake-promoting systems during REM sleep—reveals how the brain achieves a remarkable and highly adaptive state: it is internally hyper-alert (driven by cholinergic activity and limbic activation) but simultaneously barricaded from both trivial external stimuli and a full transition to wakefulness (via the inhibition of these key arousal systems). This creates a “sleeping sentinel” that is primed to respond to specific, significant threat-related stimuli, as demonstrated by Tseng and colleagues (2022), but is not constantly disrupted by minor sensory input. The silencing of the LC-NE system, for example, is not a contradiction to a vigilance theory, but a crucial feature that allows the sentinel to remain asleep while on duty.

***7. N-REM sleep is heterogeneous*.** Another attempt to refute my arguments is to point out the fact that N-REM sleep is not a homogeneous sleep state in terms of reduced capacity to awaken (Kjaerby et al., 2022). N-REM sleep encloses a complex microarchitecture that includes periodic episodes of micro-awakenings (Kjaerby et al., 2022). Superficially, this fact might seem to present some refutation. However, as with the other examples I provided above, a more careful analysis reveals corroboration rather than refutation. According to the arguments I presented in Section 4.5, REM sleep probably debuted in the form of a brief awakening from N-REM sleep. *Therefore, the presence of micro-awakenings during N-REM sleep only reinforces my argument that REM sleep evolved from a brief awakening from N-REM sleep*. If episodes of micro-awakenings already occurred in the distant past, an error in one of them is precisely what could have caused the brief awakening from N-REM sleep—the event I argued was the primeval occurrence of what later became REM sleep as we know it.

Considering that, at present, N-REM micro-awakenings and REM sleep itself appear to be distinct neural phenomena in terms of function and neuronal circuitry (Kjaerby et al., 2022; Luppi et al., 2024), the following question may arise. How could two distinct physiological phenomena share similar evolutionary origins? Well, this is exactly the result of non-random elimination of individuals over countless generations—that which we know as evolution. The fact that two contemporary physiological phenomena differ in their form and function does not imply that they have separate origins. It remains entirely possible that they both arose from a common evolutionary precursor. Indeed, the principle that distinct traits can share a common evolutionary origin is one of the cornerstones of evolutionary biology (Darwin, 1859; Dawkins, 1997; Mayr, 1982).

Therefore, the mere present-day difference between REM sleep and the micro-awakenings occurring during N-REM sleep cannot be used to refute my hypothesis that REM sleep evolved from brief awakenings. We must also remember the methodological limitation that sleep leaves no fossil record. Thus, we are unable to access data that would allow us to reconstruct a relatively precise history of sleep’s evolution throughout the evolution of organisms.

***8. In certain situations, waking up from N-REM sleep may be easier than waking up from REM sleep*.** Another piece of evidence that seems to refute the Sentinel Sleep Theory is provided by the experiment of Siegel and Langley (1965), whose findings suggest that, on some occasions, it may be easier to awaken from deep sleep than from REM sleep. However, once again, we must interpret this with caution. Based on the sentinel function, we should (obviously) expect a greater ease of awakening during REM sleep. But it is naïve to conclude that this ease applies to *every* type of stimulus. The stimulus that Siegel and Langley used to awaken both cats in the experiment was a series of clicks of varying duration and intensity. More importantly, the cats had learned to associate the end of a series of clicks with a reward: milk. Among other findings, Siegel and Langley (1965) reported that, when exposed to clicks during sleep, the cats awakened more easily during deep sleep than during REM sleep.

However, this result does not refute the Sentinel Sleep Theory. As I am arguing, REM sleep evolved to be *responsive to danger*, not to stimuli associated with an appetitive reward. And, as demonstrated by Tseng and colleagues (2022), REM sleep is indeed particularly responsive to danger. Furthermore, throughout the conditioning and testing phases of Siegel and Langley's experiment, the cats were deprived of food and water. Thus, the results also suggest that N-REM sleep is more responsive to food-related stimuli than REM sleep—especially, perhaps, when the animal is deprived of water and food. What would the results have been if the clicks had been associated with shocks or any other negative and harmful experience for the body? I predict that the clicks would have awakened the cats much more easily during REM sleep than during N-REM sleep. Another prediction: if it is not a dangerous stimulus (e.g., associated with a predator), I predict that the intensity of the stimulus will have to be greater to awaken the organism from REM sleep. Which is exactly what Siegel and Langley's results show.

***9. Brain temperature and REM sleep*.** Let us now consider another way to try to challenge my theory. In most mammals studied to date, brain temperature (both cortical and subcortical) increases during REM sleep and decreases during N-REM sleep (Ungurean et al., 2020; Wehr, 1992). This led many scientists to believe that the primary function of REM sleep is to regulate cortical temperature. The first scientist to propose this hypothesis was Thomas Alvin Wehr, in 1992. However, if the function of REM sleep were to warm the brain, then such warming should also occur in other animals (beyond mammals) that exhibit REM sleep or its analog (Ungurean et al., 2020). But this is not the case. In bearded dragons, cerebral temperature does not increase when the brain transitions from N-REM sleep to REM sleep (Ungurean et al., 2020).

Moreover, the evidence indicates that the brain warms during REM sleep merely as a result of increased blood flow, which is necessary for the brain to carry out REM sleep (Bergel et al., 2018; Denoyer et al., 1991; Parmeggiani, 2007; Pastukhov and Ekimova, 2012; Wehr, 1992). (See also the study by Ungurean and colleagues [2020], in which the authors challenge the notion that the primary function of REM sleep is to warm the brain.) This body of evidence demonstrates that the brain warming associated with REM sleep may be merely a functionless byproduct, arising from mechanisms that require increased neuronal activity—such as the sentinel function. Therefore, the fact that brain temperature rises during REM sleep supports (rather than refutes) the Sentinel Sleep Theory. This is because the sentinel function requires increased neuronal activity—a demand that may elevate brain temperature (albeit subtly).

To better understand how REM sleep relates to temperature, we must also consider that low temperatures can reduce blood flow (Bisschops et al., 2010; Croughwell et al., 1992; Ibayashi et al., 2000; Mrozek et al., 2012; Shepherd et al., 1983). Accordingly, since REM sleep requires increased blood flow while the brain executes it, low temperatures—by virtue of their capacity to reduce blood flow—may suppress REM sleep. This can explain why, in mammals exposed to low temperatures, REM sleep is selectively and strongly suppressed (Amici et al., 1998; Amici et al., 2008; Cerri et al., 2005; van Hasselt et al., 2024).

***10. Immune system and REM sleep*.** To end this Section, I will address evidence related to the immune system. This is another line of evidence that, at first glance, appears to refute my arguments. But we just need to analyze more carefully to see that, in fact, they corroborate it. When disease-causing bacteria invade the body, its entire functioning is profoundly altered to respond to the invasion (Giamarellos-Bourboulis and Raftogiannis, 2012; Krueger et al., 1982). In humans, REM sleep was significantly reduced in response to *Salmonella abortus* endotoxin (Pollmächer et al., 1993). In rabbits inoculated with *Staphylococcus aureus*, from 6 to 10 hours after inoculation, REM sleep was suppressed, remaining at low levels for the rest of the recording period (Toth and Krueger, 1988).

Two other notable findings from the Toth and Krueger (1988) study are that (1) antibiotics (cephalothin) attenuated the effects of this bacterial infection on sleep and (2) inoculation with dead bacteria also produced analogous changes in sleep and other physiological parameters. In summary, during the progression of most infections, REM sleep will, at some point, be inhibited or experience a reduction in its duration (Fang et al., 1995; Imeri and Opp, 2009; Krueger and Majde, 1994).

Some scientists might conclude, based on the above evidence, that it refutes the Sentinel Sleep Theory by assuming that infection leaves the organism vulnerable and, therefore, this vulnerability would increase (rather than reduce) REM sleep. However, this conclusion is incorrect. This is because the vulnerability arises from what the antigen is doing to the body and from its homeostatic effects if the body fails to manage the infection. Furthermore, we must consider that the immune system is a mechanism whose function is *to protect* the organism from bacteria, viruses, parasites, and toxic molecules capable of invading its body (Damasio, 2003, p. 31). The immune system is the first line of *defense* that *protects* vertebrates from threats affecting their integrity, whether arising from within their own body or from external sources, due to an infection (Damasio, 2003, p. 31).

Therefore, since antigens (e.g., bacteria) trigger an immune response composed of numerous antibodies capable of neutralizing the invader (Damasio, 2003, p. 58), the body is flooded with proteins that serve to make it *better protected* against the threat. And what happens to REM sleep when the body is more protected? Naturally, it decreases. As the parameters of REM sleep are determined by interoceptive information, during an infection, interoceptive signals inform the brain that the organism is inundated with protective proteins—especially at the peak of the immune response. This explains why REM sleep decreases rather than increases.

My conclusion is supported by the fact that the administration of cytokines—proteins that play an important role in the immune system—suppresses REM sleep (Opp, 2005). Therefore, the evidence concerning the immune system and its relationship with REM sleep (Fang et al., 1995; Imeri and Opp, 2009; Krueger and Majde, 1994; Pollmächer et al., 1993; Toth and Krueger, 1988) supports the Sentinel Sleep Theory as a whole and, more specifically, *hypothesis 20* of Table 2.

Based on what I am arguing, a crucial question may arise. I am defining an active immune response as “protective,” thereby explaining why an infection inhibits or suppresses REM sleep. However, a state of illness is unequivocally a state of homeostatic deviation and increased vulnerability, even to other threats (e.g., predation). So how can this apparent contradiction be reconciled? The answer is simple: we must separate the information from the neural maps that register the threat (a state of vulnerability) from the immune response to that threat (a state of protection).
 It is clearly true that infection disrupts the organism’s biological regulation, causing the body and the brain to map the homeostatic deviation associated with that infection. This is another way of saying that when a pathogen infiltrates an organism, it places the organism in a vulnerable state. However, it is crucial to distinguish between the neural mapping that signals the threat associated with the homeostatic deviation caused by the infection, on the one hand, and the defensive response to the infection, on the other. We must remember that when the brain and body map a state of vulnerability, they may also respond to it. And when such a response occurs, it increases the organism’s internal protection by providing the appropriate chemical means to cope (as far as possible) with that threat. In other words, vulnerability is established by the neural maps that the brain and body construct to signal how the antigen disrupted (and to what extent it disrupted) the biological regulation. But we must also keep in mind that, as a consequence of this mapping of the infection, the brain and body can respond defensively.

Therefore, by considering how a neurochemical and physiological response to a threat differs from the neural mapping that signals that threat, we can understand and explain why and how an infection affects the parameters of REM sleep.

If the mechanisms and pathways that control REM sleep were conscious agents, it would probably be prudent for the brain, during an infection, to disregard the interoceptive information from the immune response and intensify the sentinel function. After all, an infection objectively places the organism in a vulnerable condition. However, we must always remember that the mechanisms and pathways that control REM sleep *are not conscious agents*. Nor are the evolutionary processes that shaped them. What matters here is that REM sleep is guided and modulated by interoceptive, proprioceptive, and exteroceptive information. This implies that the immune response during an infection will inevitably affect the parameters of REM sleep.

Another nuance that we must consider is that scientific studies of immune system responses in humans are based on a balance between a measurable physiological effect and the safety of the volunteers. And although the limits are somewhat more flexible in nonhuman animals, this balance also applies to them. This is important because it inevitably creates a disparity between a realistic infection scenario and a more controlled and safer one. In more controlled infections, there is a considerable disproportion between the infection and the immune response to that infection. A controlled infection does not perturb biological regulation as intensely and, therefore, causes the immune response to carry more weight than the neural mapping that signals the state of vulnerability. In other words, in a more controlled and safer infection, the interoceptive information from the immune response ends up taking precedence over the interoceptive information that maps the homeostatic threat of the infection. For this reason, we must remember that a controlled and clinically safer infection may still elicit a robust immune response. This is, after all, what underlies the utility of vaccines.

This argument of mine—consistent with evidence from the immune system—suggests that the circuits regulating REM sleep are sensitive to interoceptive, proprioceptive, or exteroceptive information that signals a neurochemical and physiological condition whose effects are more pronounced. Thus, if a protective response (e.g., cytokines) exerts stronger effects than the vulnerable state that elicited it, then the neural signals of the protective response will predominantly influence REM sleep parameters. Conversely, if a state of vulnerability produces stronger effects than a protective response (e.g., cortisol), then the neural signals of that vulnerable state will predominantly influence REM sleep parameters. As we saw in Section 4.4, this is the case of the failed attempt to inhibit REM sleep in depressed patients with the acute application of cortisol. This argument constitutes a testable prediction of my theory and must, within ethical limits, be exhaustively tested going forward.

**S2. Limitations**

As I reported before, due to the lack of studies, I was unable to verify the *hypotheses 26, 29, 32, 43, 44, 46, and 47* of Table 2. Those that refer to premature births are particularly difficult to test, as the presence of REM sleep in fetuses (although likely) is inconclusive (Okawa et al., 2017). What causes this difficulty is the fact that scientists cannot use the electroencephalogram (or EEG) on a fetus in the same way as they would on an adult; this is relevant because scientists rely on EEG data to identify sleep states (Okawa et al., 2017). Note that this lack of studies is not a weakness of the theory, but rather a testament to its ability to generate numerous new testable hypotheses. By identifying these specific knowledge gaps, the Sentinel Sleep Theory provides a clear and productive roadmap for future empirical research, which is a key function of a valuable scientific theory.

Due to the length of my Article, I was unable to address how all other hypotheses previously proposed for the function of REM sleep fit into the conceptual framework of the Sentinel Sleep Theory—a significant gap that we need to address in future work. This limitation, however, does not pose an epistemic threat to my theory. This is because, as I argued in Section “S6” of the Appendix A, my theory solves a greater—and more significant—number of empirical and conceptual problems than any rival hypothesis. And it also has fewer anomalous problems relative to rival hypotheses.
 A more significant limitation is the fact that I was unable to delve deeper into the circuits underlying the Sentinel Sleep Theory. While I outlined some contributions here, this is a work for future empirical and theoretical research. For example, some of the physiological questions of vulnerability and its association with REM sleep parameters that still require further elucidation may be resolved by studying the neural circuits of somatosensory regions and the neurophysiology and neurochemistry of such circuits.

**S3. A significant and detrimental consensus**

Since the current *zeitgeist* is dominated by a consensus towards the hypothesis that the function of REM sleep is to aid learning (Bear et al., 2016, p. 665; Gazzaniga et al., 2016, pp. 150-151; Kandel et al., 2021, pp. 1091-1092; Moruzzi and Eccles, 1966; Ribeiro, 2021), I need to address this issue. After all, this consensus can hinder the proper understanding of the true biological function of REM sleep.

As pointed out by Capellini and colleagues (2008) and Ribeiro (2021), the hypothesis that a function of REM sleep is to aid learning and memory consolidation led many scientists to argue that species with considerable cognitive abilities should require more time invested in REM sleep. However, dolphins—animals whose high intelligence is well-established—do not have REM sleep, while armadillos—less intelligent animals—have it in abundance (Ribeiro, 2021). Moreover, if REM sleep played a crucial role in learning, patients medicated for depression (who experience reduced or suppressed REM sleep) should exhibit learning deficits. But why do they not present it? Why is the time spent in the REM period not strongly correlated with learning in humans (Ribeiro, 2021)? I will discuss henceforth a study that, among other objectives, analyzed the REM sleep learning hypothesis by comparing numerous species.

Capellini and colleagues (2008) conducted their research using a database that, as of June 29, 2007, contained records of REM and N-REM sleep from 127 distinct species across 46 families and 17 orders. The researchers also compiled information about laboratory procedures, as different laboratory conditions and measurement methods can affect data analysis (Campbell and Tobler, 1984; Siegel, 2005). As reported by Capellini and colleagues (2008), after controlling the laboratory conditions and phylogeny, the research results *did not support* any of the traditional explanations claiming that REM or N-REM sleep serves to benefit cognition, aid brain development, or to conserve energy. The evidence from Capellini and colleagues (2008) demonstrates that, despite the association with learning, REM sleep does not play a critical role in it. To further reinforce the argument that REM sleep does not play a critical role in learning, I will present henceforth additional evidence from genetics.

Whenever a neuron undergoes the process of encoding a new memory, coding genes capable of modifying synapses are promptly activated. The so-called *Immediate-Early Genes* (IEGs) are the first genes involved in this process, being activated a few minutes after neuronal electrical reverberation begins (Abraham et al., 1991; Bahrami and Drabløs, 2016; Davis et al., 2003; Ribeiro, 2021). A specific number of IEGs need to be expressed for other genes essential for consolidating long-term memories to also be expressed (Abraham et al., 1991; Davis et al., 2003; Okuno, 2011; Ribeiro, 2021).

Considering the hypothesis that a function of REM sleep is learning—and that IEGs are necessary for long-term synaptic modifications to be caused and for memories to be formed (Okuno, 2011; Ribeiro, 2021)—one would expect to find an increase in IEGs expression during sleep, especially during REM sleep. However, in organisms not exposed to new stimuli during wakefulness, sleep (including REM sleep) strongly suppresses the expression of IEGs rather than increasing it (Decker et al., 2010; Pompeiano et al., 1992; Pompeiano et al., 1994; Pompeiano et al., 1995; Pompeiano et al., 1997; Ribeiro et al., 1999; Ribeiro, 2021). The expression of IEGs increases during REM sleep, but not during N-REM sleep, only when the organism is exposed to new environmental stimuli during recent wakefulness (Ribeiro et al., 1999; Ribeiro, 2021).

What may explain this disparity in the expression of IEGs is the difference between the functions of N-REM and REM sleep. Whatever the function (or functions) of N-REM sleep may be, it does not serve as a sentinel mechanism. The sentinel function is carried out by REM sleep, and it is precisely this function that may explain why the expression of IEGs increases during REM sleep but not during N-REM sleep after the organism is exposed to new—and therefore potentially dangerous—environmental stimuli. In other words, the sentinel function of REM sleep seems to explain very well why the expression of IEGs is suppressed during N-REM sleep even after new environmental stimuli are presented during recent wakefulness. For this assertion to make more sense, I need to elaborate it further.

Registering information (i.e., learning about the surrounding environment and its components, including predators) is a substantial adaptive advantage (Damasio, 2012, pp. 67-68; Damasio, 2019, pp. 61-62). As I discussed earlier, when an organism is exposed to new information (or stimuli), IEGs are promptly activated. A noteworthy aspect of the expression of these genes is that it constitutes a protective mechanism, and it is easy to see why. Let us compare an organism whose IEGs expression takes a long time—so long that we could call them very late genes—with one whose expression truly deserves the term “immediate.” In the first case, the expression of genes essential for forming and consolidating new memories takes so long that, after interacting with a predator, the prey’s brain is unable to quickly modify its synapses to register the new (and biologically relevant) information obtained from that encounter. In the second case, the brain can quickly modify synapses and register new memories related to that predator. The second organism has a clear adaptive advantage over the first. But why does this swift expression of genes involved in memory formation and consolidation make it more protected?

Memories are biologically valuable especially because they allow the organism to store relevant information for its own survival—derived from both the external environment and the body’s internal milieu (Damasio, 2012, pp. 67-68; Damasio, 2019, pp. 61-62, 75-83). Therefore, it is advantageous to respond to sensory novelties with an immediate expression of genes related to the formation and consolidation of new memories because one never knows when such novelty will bring danger with it. Although most of the time the number of neutral stimuli is greater than the number of dangerous ones, it is advantageous to always promptly activate the aforementioned genes precisely because of the times when danger is present. When sensory novelty includes danger, the organism whose memory formation process is faster has a significant advantage over those whose memory formation process is slower—an advantage that can mean the difference between life and death.

This is why an organism that more quickly and effectively stores sensory information obtained from a predator after encountering it is comparatively less vulnerable to it. If you escape from that predator during the first encounter and it (or another of the same species) returns to attack you within a few minutes, the information obtained—and quickly learned by the brain after the first encounter—will make you better protected. After all, the information stored about the predator (e.g., how and where it attacked you) increases your chances of surviving if attacked again by the same predator or any other of the same species. Knowing that you need to avoid its claws or tail makes you better protected compared to a contemporary of yours who did not store this information.

The more information you gather about a predator, and the more quickly your brain registers it, the better you can defend yourself when it attacks you again. Knowing your enemy's attack tactics and typical behaviors increases your chances of staying alive when dealing with it. The more you know your enemy, the better protected you can be from him. This is why *the expression of IEGs constitutes a protective mechanism during wakefulness; it ensures that the organism learns quickly when danger is a possibility*. This brings me to the final part of my argument.

Since IEGs expression constitutes a protective mechanism *during wakefulness*, it is possible to explain—through the sentinel function of REM sleep—its increased expression during REM sleep but not during N-REM sleep after the organism has been exposed to new environmental stimuli. The potential danger inherent in sensory novelty induces the expression of IEGs during wakefulness. Since the function of N-REM sleep is not to serve as a sentinel mechanism (Tseng et al., 2022), nor is it critically related to learning (Capellini et al., 2008), IEGs expression is suppressed during this sleep state. However, things change during the REM period. Since the primary function of REM sleep is to serve a protective role, IEGs expression reoccurs.

The reason this occurs is simple: the expression of IEGs during wakefulness serves to reduce the organism's vulnerability when new stimuli are received. And since the function of REM sleep is also to reduce the organism's vulnerability, the expression of IEGs occurs during REM sleep whenever the organism has recently been exposed to new environmental stimuli. When a significant vulnerability is presented during wakefulness, a protective mechanism that deals with it—the expression of IEGs—is reactivated during REM sleep. This only happens due to the protective function of REM sleep. A notable consequence of this is that, if a predator attacks an organism during REM sleep and after this organism has recently been exposed to new stimuli during wakefulness, the information obtained from that attack will be promptly stored by the brain due to the elevated expression of IEGs. Therefore, considering the sentinel function of REM sleep, what would be strange is if the expression of IEGs were suppressed during this sleep period after recent exposure during wakefulness to new stimuli.

What many scientists failed to realize is that REM sleep's contribution to memory consolidation may be a *byproduct* of its primary function, not the function itself. Indeed, the evidence that Capellini and colleagues (2008) provided indicates exactly this. Consequently, scientists who believe that learning is the function of REM sleep appear to be following the wrong path rather than the right one. If this is indeed the case, the excessive insistence on the learning hypothesis to explain REM sleep’s function will prove to be an inappropriate trajectory for solving this mystery, responsible for guiding scientists away from the true answer instead of closer to it.

But how exactly can REM sleep contribute, as a byproduct, to memory consolidation? We known for a long time that memories must be continuously reactivated in order to exist and be preserved over time (Gazzaniga et al., 2016, p. 268; Kandel et al., 2014; Martin et al., 2000; O’Leary et al., 2024; Pignatelli et al., 2019; Ryan and Frankland, 2022; Swaab et al., 2002; Tononi and Cirelli, 2014). We also know that memories are not reactivated based on the order in which they were learned, but rather on their biological value (Damasio, 1996; Damasio, 2012; Damasio, 2019).

Combining these two lines of evidence, we arrive at the following conclusions. *The memories that reverberate most frequently in the mental flow are those with emotionally relevant content*. And this applies especially to sleep. After all, during wakefulness the organism is inundated with stimuli from the senses, and the brain must update the responses to numerous important questions (Kahneman, 2011; Ribeiro, 2021): Did something new occur? Has a threat emerged? Is homeostatic regulation functioning properly? Should attention be redirected to another event or stimulus? Does this task require additional energy?

Therefore, neural reverberation based on the importance of memories is particularly relevant during sleep to determine which memories reverberate most often in the mental flow. (I use the term “mental flow” to refer to the electrically active neural patterns in the central nervous system due to memories of current or evoked stimuli, actions, or events.) In turn, understanding which memories reverberate most frequently in the mental flow helps us identify which memories are likely to be best preserved (i.e., learned).

But why does REM sleep appear to contribute especially to learning? The answer depends both on neural activation driven by the biological value of memories and on which brain regions are active during REM sleep. The areas of the brain that become active during sleep—and the intensity of that activation—vary considerably and are directly linked to the two main sleep states: REM sleep and N-REM sleep (Han et al., 2024; van der Helm and Walker, 2011). REM sleep, for example, is associated with high neural activity in the occipital cortex, thalamic nuclei, pontine tegmentum, mediobasal prefrontal lobes, as well as in affect-related regions including the amygdala, hippocampus, and anterior cingulate cortex (van der Helm and Walker, 2011). Given the diversity of neural activation across these specific sleep states, REM and N-REM sleep play distinct roles in the memory consolidation process (van der Helm and Walker, 2011).

Although scientists traditionally associated REM sleep with a particular capacity for consolidating emotional memories (Groch et al., 2013; van der Helm and Walker, 2011), a recent study provided evidence that both N-REM sleep and REM sleep contribute to consolidating emotional memories (Yuksel et al., 2025). This evidence reinforces my argument that it is the *frequent activation* of memories within the mental flow—based on their biological value—that drives their consolidation. It is not REM sleep itself that functions to consolidate memories, but rather the electrical activation of those memories during REM sleep—and considering the neurally active regions due to the function of REM sleep—that contributes to their consolidation.

This is why sleep (in general) appears to prioritize the consolidation of emotional memories (Payne et al., 2008; Zhong et al., 2024). *My argument is that this is not a function of sleep—neither N-REM nor REM—but merely a byproduct of the electrical activation based on the biological value*. During sleep, without the overwhelming interference of sensory information from wakefulness, neural electrical activation depends particularly on the importance of the memory. Consequently, it is the emotional memories that reverberate most frequently during sleep and—precisely because of their electrical activation—end up being better consolidated.

At this point, it is worth recalling that one of the neural regions with heightened activity during REM sleep is the hippocampus: a crucial region for memories to be consolidated (Fogwe et al., 2023; Lee and Wilson, 2002; Nolan, 2023; Squire et al., 2015). Moreover, there is evidence that it is hippocampal neural activity during REM sleep (related to theta rhythm) that is involved in memory consolidation (Dragoi et al., 1999; Poe et al., 2000; Stickgold and Walker, 2007; Montgomery et al., 2008; Nishida et al., 2009; Rattenborg et al., 2011). Indeed, Poe and colleagues (2000) provided empirical evidence that it is the *repetition* of neural activity during REM sleep that contributes to memories being consolidated. This is consistent with classical evidence that memory repetition contributes to their retention (e.g., Ebbinghaus, 1913; Hebb, 1961) and also with more recent evidence (e.g., Dudai and Eisenberg, 2004; Karpicke and Roediger, 2008; Yu et al., 2024). There is also evidence that, during N-REM sleep, the electrical reactivation of hippocampal memories similarly contributes to their consolidation (Lee and Wilson, 2002; Nádasdy et al., 1999).

Additional evidence for my claim that it is electrical activation—not REM sleep—that contributes to consolidation comes from the study by Bass and colleagues (2012). In that work, the authors reported that electrical stimulation of the basolateral amygdala complex resulted in markedly improved memory. The following studies likewise reported enhanced memories due to electrical stimulation (Bass et al., 2014; Bass and Manns, 2015; Geva‑Sagiv et al., 2023).

In sum, considering all the evidence above, it is clear that the reason REM sleep contributes to consolidating memories is due to the neural mechanism of electrically activating memories based on their biological value—something that, as I demonstrated, is not confined to REM sleep. N-REM sleep, by that same neural mechanism, also contributes to consolidating emotional memories (Yuksel et al., 2025). Thus, this mechanism explains why sleep—especially REM sleep—plays a critical role in emotional processing (Tempesta et al., 2018) and in psychological health (Kollar et al., 1969). To further clarify what I am proposing, I am not claiming that memory consolidation associated with REM sleep is a byproduct of the sentinel function itself, but rather a byproduct of the neuronal electrical activation that occurs during REM sleep, which, in turn, is associated with the sentinel function.

Neural reverberation being based on biological value and neurally active regions also allows us to reinterpret the proposal of “reverse learning” (Crick and Mitchison, 1983). After all, if spontaneous electrical activation depends on the biological value of memories, then a logical consequence is that less important memories will be activated less frequently. The consequence of this, in turn, is that they will tend to be unlearned after sufficient time passes without being electrically active (Tononi and Cirelli, 2014). That is, while sleep contributes to consolidating important memories, it also contributes to deconsolidating those that are infrequently activated. This explains why REM sleep both forms and eliminates synapses (Li et al., 2017). Note that this mechanism is not confined to REM sleep. Because memories are activated according to their biological value, both REM and N-REM sleep passively contribute to the weakening of less important (or effectively unimportant) memories. Although it is obvious that we should expect the effects of this weakening to be distinct in REM sleep and N-REM sleep, precisely because the neural regions active during these states vary (van der Helm and Walker, 2011).

Finally, the aforementioned mechanism also helps to explain why REM sleep is so strongly associated with dreaming—a strong and well‑established relationship we known for a long time (Desseilles et al., 2011; Gazzaniga et al., 2016, p. 146; Martin et al., 2020; Ribeiro, 2021; Solms, 2000). After all, with more intense neural activation—especially in the limbic system—it is natural that dreams occurring during this sleep stage are more vivid, more intense, and more emotional.

To conclude this Section, I will address a question that may arise within the scientific community: Am I creating a false dichotomy by proposing that memory consolidation associated with REM sleep is a byproduct? Are the two functions mutually exclusive? Or is it possible that they are deeply interconnected? The very process of consolidating memories of emotionally salient events and stimuli—especially threats—is, in itself, a form of long-term vigilance. After all, consolidating what is dangerous is crucial for future survival (Damasio, 2012; Damasio, 2019).

*What if, instead of being rivals, the Sentinel Sleep Theory and the memory consolidation hypothesis are two sides of the same coin?* The neural activity during REM sleep could serve both purposes simultaneously: enhancing the organism’s alertness to present dangers (real or dreamed) and providing the neurochemical environment necessary for more effective memory consolidation. Such a synthesis would not weaken my theory. On the contrary, it would integrate what is probably the leading competing hypothesis, elevating it to a fundamental feature of the theory. This is very tempting, but we must proceed with caution.

To better evaluate this integrative possibility I just outlined, it is necessary to distinguish between the biological function of REM sleep, on the one hand, and the biological functions of memory, on the other. *Memory* here is understood in the general sense applied to living beings: the capacity of a nervous system to acquire, form, consolidate, and retrieve information (Bisaz et al., 2014; Izquierdo, 2018, pp. 1, 4).

One of the primary functions of memory is, unquestionably, to regulate life. For this reason, life management and memory storage are intimately connected (Bisaz et al., 2014; Damasio, 2012; Damasio, 2019; Forester et al., 2020; Thornton and Boogert, 2019). This clearly includes the ability to map threats that arise within the range of any sensory modality. Organisms that map threats have higher chances of survival, since encountering the same threat again will trigger a state of vigilance and, if necessary, a state of fight or flight (Chand et al., 2021; Damasio, 2012; Damasio, 2019; Oken et al., 2006). That is, it is true that consolidating information from emotionally competent events and stimuli—especially threats—constitutes a form of long-term surveillance, thereby ensuring biological regulation. However, what is pivotal here is recognizing that this is one of the functions *of memory*, but not necessarily of REM sleep. Thus, the most parsimonious explanation, based on the evidence I discussed, is that memory consolidation associated with REM sleep is indeed a byproduct, rather than one of its functions.

A final caveat: The arguments I developed and the evidence I presented to demonstrate that memory consolidation is not a function of REM sleep do not imply that such consolidation does not occur. In other words, I am not denying the fact that REM sleep contributes to memory consolidation. The evidence on this point is very clear. Therefore, to deny that REM sleep contributes to memory consolidation is strictly incorrect. Note, however, that claiming that this consolidation occurs is entirely different from claiming that it occurs because it is a function of REM sleep. Although REM sleep does contribute to memory consolidation, I believe I provided sufficient arguments and evidence to demonstrate clearly that this is not its biological function.

**S4. On the relationship between REM sleep duration and sentinel function**

A pertinent issue that I need to address concerns the duration of REM sleep in relation to its protective function. In each N-REM/REM cycle, the N-REM period lasts longer than the REM period (Brinkman et al., 2023; Kandel et al., 2013, p. 1143). Does this undermine my theory? Can REM sleep still be considered adaptive? The reason I devoted time to discussing the importance of N-REM sleep is precisely to demonstrate that it is a *non-negotiable necessity*. It cannot be dispensed with. As I argued in Sections 3 and 4.1, eliminating N-REM sleep is (apparently) impossible and non-adaptive; if it were relatively easy to eliminate it, such a characteristic would have spread, since it would certainly be adaptive. It logically follows that drastically reducing N-REM sleep time is also not an adaptive possibility—and that is what would need to happen for REM sleep to predominate. In other words, because N-REM sleep is so imperative, it consequently predominates during rest periods. It must predominate in order to fulfill its function. The result is that, with N-REM sleep necessarily occupying most of the sleep period, less time remains for REM sleep. However, this in no way undermines my theory.

For those who believe that the short duration of REM sleep impedes the sentinel function, it is worth recalling that 50% of an eye like ours is better than 35% of it. More importantly: 1% of that eye is better than having no eye at all (Dawkins, 2015, pp. 112-113). During sleep, an organism having *some* period in which the brain is distinctly more responsive to potentially harmful stimuli is better than *having no* such period at all. Even if the organism remains vulnerable for most of the rest period (due to N-REM sleep), possessing a state of heightened alertness—even if brief—is still advantageous. *The fact that REM sleep does not predominate over N-REM sleep does not imply that REM sleep is useless or non-adaptive*.

It is an absolutist line of thinking to assume that, in order to be functional, REM sleep must last longer than N-REM sleep. Arguing that REM sleep can only be adaptive if it predominates in duration is analogous to claiming that an eye can only be adaptive if it already possesses, for instance, a well-developed lens, optic nerve, and retina. Therefore, the presence of the sentinel function, even if only for a short period, is more adaptive than its absence. Indeed, this is why, as I argued in Section 4.2, REM sleep occurs repeatedly throughout the rest period.

**S5. Additional comments on scientific research linking REM sleep to body fat and muscle mass**

Next, I will comment on some of the studies I mentioned in Section 4.4. This will allow me to better explain some results from the perspective of Sentinel Sleep Theory.

In the study by Chamorro and colleagues (2014), the authors concluded that being overweight in childhood is associated with changes in total sleep duration, in N-REM sleep, and in REM sleep. Something particularly relevant to my theory is that *the amount by which REM sleep was reduced in overweight children was inversely proportional to body mass index*. The age of the children was almost identical in both groups, and sleep patterns were recorded under natural conditions. Therefore, as described by the researchers, these factors (which alter REM sleep) are unable to explain the discrepancy found in REM sleep between the two groups. This discrepancy is easily explained by the Sentinel Sleep Theory.

In the study by Kitamura and colleagues (2021), the difference found between men and women is exactly what would be expected due to the sentinel function. Since the men had greater muscle mass than the women, this made them more protected, thus requiring less time in REM sleep.

In the study by Liu and colleagues (2008), to determine which sleep stages (N1, N2, delta sleep, and REM sleep) were independently related to being overweight, the researchers conducted a multiple logistic regression analysis. The result was that only the reduction in REM sleep was independently and significantly related to being overweight (*p* = 0.03).

In the study by Myllymäki and colleagues (2011), the researchers reported that REM sleep had an average duration of 88 minutes in the exercise situation and an average duration of 101.3 minutes in the no-exercise situation (*p* = 0.155). From the perspective of Sentinel Sleep Theory, this result indicates that engaging in high-intensity exercise—even for just one day and within three hours before sleep—is enough to virtually reduce the organism's vulnerability. The mere fact of exercising intensely before sleep causes the neural mechanisms that regulate REM sleep to interpret this action as a small, but significant, increase in the organism's protection, which supports *hypothesis 20* from Table 2.

**S6. How to assess the adequacy of a scientific theory?**

Attempting to answer the question that titles this Section with a single criterion would be far too incompatible with best scientific practices—and also incorrect. We, scientists, must assess how adequate a theory is on the basis of multiple criteria rather than only one. And that is precisely how it should be. The quality and adequacy of a scientific theory cannot be evaluated by appealing to a single sufficient criterion.

In Section 2, I presented seven criteria that I employed to test my theory. In this Section, I will dwell somewhat more on the fifth criterion from that list—a theory’s capacity to solve problems. I will do so because many scientists, when considering a theory, tend to value almost exclusively its empirical support. I will argue that, although the extent to which a theory has been empirically corroborated is important, what matters somewhat more are the problems—especially the most significant ones—that it resolves.

When developing scientific theories, one hopes to coherently and adequately solve significant empirical and conceptual problems (Laudan, 1977, p. 70). Furthermore, scientists also develop theories to avoid or resolve the numerous conceptual and empirical problems that predecessor hypotheses or theories have (Laudan, 1977, p. 70). Larry Laudan (1977, pp. 29, 66) proposed that, if two theories compete with one another, we should regard as better and more preferable (or truer) the one that proves more successful both in solving significant problems in a convincing and meaningful way and in resolving a greater number of problems. Mayr (2001, pp. 54, 77) endorses this method of choosing between rival theories, as do I.

I agree with Laudan (1977, pp. 5, 13-14) when he states that a theory’s competence to solve *relevant* problems is more important than the extent to which it has been confirmed or refuted. However, I must clarify a few points. Laudan (1977) argues that the best criterion for evaluating a scientific theory is its capacity to solve significant problems. The issue is that Laudan goes so far as to dismiss confirmation or refutation as useful and necessary criteria. In other words, Laudan disregards the truth of a theory as a criterion for evaluating it. On this point, I disagree with him. *The criterion of problem-solving is certainly pragmatic, but it must be coupled with empirical corroboration in order to have scientific value*.

To make my position clear, I am not claiming that a theory’s problem-solving capacity outweighs its empirical testing. What I am in fact asserting is that we should place *a little more* value on problem-solving. Thus, both the degree of confirmation and the capacity to solve problems are relevant, but the slightly greater weight should fall on the resolution of *significant* problems. The extent to which a theory has been empirically corroborated is a necessary criterion for any serious scientific theory—and no properly trained scientist would dare to disagree. Nevertheless, I endorse the view that a theory’s capacity to solve relevant problems should be regarded as even more significant. And this is by no means incompatible with the pluralism of criteria that we ought to adopt in evaluating scientific theories. After all, advocating a plural criteria does not necessarily commit us to granting equal weight to each of them. *No individual criterion is sufficient, but some may carry more weight than others*.

Many scientists, when questioning a theory’s adequacy or robustness, tend merely to count the number of facts (or pieces of empirical evidence) that corroborate or contradict it. However, what we should also consider—and give greater weight to—is the theory’s capacity to adequately solve significant empirical problems (Laudan, 1977, pp. 13-14). Moreover, for a theory to be regarded as more adequate than its rivals, it must entail a smaller number of anomalous problems; a property that is closely related to the theory’s problem-solving capacity (Laudan, 1977, pp. 18, 30-31, 66).

The objectives of this Section are: (1) to assess whether my theory meets the criterion of adequately solving significant empirical problems in the domain of REM sleep; and (2) to compare my theory with rival hypotheses with regard to the number of anomalous problems.

According to Laudan (1977, p. 29), whenever a theory solves an empirical problem *P*, then *P* henceforth becomes an anomaly for every rival theory that is unable to solve *P*. An *anomaly* here denotes an empirical problem that a given theory cannot resolve but that one or more rivals are able to resolve (Laudan, 1977, p. 17). With respect to REM sleep, my theory lacks rival theories capable of explaining and resolving it—what exists are rival hypotheses. Considering the number of significant empirical problems my theory solves, all of them become anomalies for any rival hypotheses that are unable to resolve them convincingly. In other words, the Sentinel Sleep Theory creates a considerable quantity of anomalous problems—both empirical and conceptual—for all rival hypotheses.

Below I list (not exhaustively) many significant problems that the Sentinel Sleep Theory either solves or contributes to solving:

(1) What is the survival value of REM sleep;

(2) Why REM sleep is biased toward potentially dangerous stimuli;

(3) Why rapid eye movements occur during REM sleep;

(4) Why REM sleep intensifies activity in the visual cortex;

(5) Why REM sleep is energetically costly;

(6) Why pronounced muscle atonia evolved;

(7) Why animals need to alternate between deeper and more active sleep;

(8) Why REM sleep occurs in cycles throughout sleep;

(9) Why REM sleep activates neural regions associated with threat detection;

(10) Why REM sleep activates neural regions associated with emotional processing;

(11) Why REM sleep activates neural regions associated with attention;

(12) Why REM sleep activates neural regions associated with pain processing;

(13) Why the amygdala is more strongly activated during REM sleep than during wakefulness;

(14) Why REM sleep (with few exceptions) is absent in animals exhibiting unihemispheric sleep;

(15) Why, upon awakening from REM sleep, the organism exhibits full alertness as well as high motor and sensory efficiency;

(16) Why REM sleep facilitates awakening;

(17) Why the amount of body fat affects REM sleep parameters (e.g., why total REM sleep time is lower in individuals with greater body fat);

(18) Why muscle strength affects REM sleep parameters (e.g., why total REM sleep time is lower in individuals with greater muscle strength);

(19) Why depression affects REM sleep parameters;

(20) Why cortisol affects REM sleep parameters;

(21) Why stress (in general) affects REM sleep parameters;

(22) Why exposure to novel stimuli affects REM sleep parameters;

(23) Why body immaturity affects REM sleep parameters;

(24) Why the immune system (particularly during its peak activity) affects REM sleep parameters;

(25) Why REM sleep facilitates learning and memory;

(26) Why the brain inhibits the LC-NE system during REM sleep;

(27) Why inhibition or suppression of REM sleep does not impair cognition;

(28) Why REM sleep must have an arousal threshold analogous to or even higher than that of N-REM sleep;

(29) Why REM sleep also involved the co-evolution of mechanisms that prevent its propensity to facilitate awakening from becoming so pronounced that it would disrupt sleep;

(30) Why REM sleep is necessary when N-REM sleep occurs in both hemispheres, but is dispensable when N-REM occurs in only one hemisphere;

(31) Why, when other factors remain unchanged, combined bodily vulnerabilities produce stronger effects on REM sleep parameters (e.g., increasing REM sleep amount and reducing its latency);

(32) Why, when other factors remain unchanged, combined bodily protections produce stronger effects on REM sleep parameters (e.g., reducing REM sleep amount and increasing its latency);

(33) Why REM sleep density is directly proportional to the amount (or level) of attention;

(34) Why REM sleep is so strongly associated with dreams;

(35) Why brain temperature increases during REM sleep.

All the hypotheses that scientists proposed for REM sleep to date can resolve only a very limited number of empirical problems, failing to solve numerous other significant empirical and conceptual problems. None of them can explain all the significant empirical problems that my theory resolves (to verify this, simply select any rival hypothesis and test it against the non-exhaustive list I presented above). In other words, my theory resolves a much larger number of significant empirical problems.

Consider the hypothesis of the defensive activation of the visual cortex (Eagleman and Vaughn, 2021). It explains why the visual cortex becomes highly active during REM sleep. However, what can it say about all the other empirical and conceptual problems associated with REM sleep? Return to the non-exhaustive list above and you will see that the defensive activation hypothesis answers nothing with respect to numerous other problems. And what of the two hypotheses favored by researchers in the field—the learning hypothesis and the memory hypothesis? They can account for why REM sleep is so strongly associated with learning and the consolidation of memories—especially emotional ones. However, they are unable to resolve numerous other important problems. To cite a few, these hypotheses neither resolve nor help to resolve problems (2), (3), (6), (9), (11), (13), (14), (15), (16), (17), (18), (19), (24), (26), (27), (28), (29), (30), (31), (32), and (33) from my list.

Since my theory resolved a large number of significant empirical problems concerning the domain of REM sleep, it becomes irrational to deny its validity. This is because one would have to explain why some rival hypothesis—which now exhibits a greater number of anomalous problems—is more valid than my theory. Moreover, we must ask—and answer—why any previously proposed *hypothesis* should be given more weight than a *theory*. And even if my theory retains some remaining anomalies, the number of anomalies in the rival hypotheses is much greater. This is important because anomalous problems constitute evidence against a theory (Laudan, 1977, p. 18); or, in this case, against the rival hypotheses. Furthermore, clearly, the empirical problems that a theory resolves count in its favor (Laudan, 1977, p. 18). Consequently, considering all the empirical and conceptual problems that my theory resolves or helps to resolve, together with its smaller number of anomalies relative to any rivals, the rational choice is to accept its adequacy and robustness.

*When assessing the merits of a theory, the problems it fails to solve are irrelevant. What truly matters are the problems—empirical and conceptual—that it does solve* (Laudan, 1977, pp. 21-22). This does not mean that unsolved problems are unimportant, either for the theory or for science. After all, one reason theories make progress is that they come to solve a greater number of problems (Laudan, 1977, p. 21). What the foregoing assertion really means is that it is irrational to attach exclusive importance to the problems a theory has not solved. For *if a theory solves more empirical and conceptual problems—specialy important ones—than any rival, we already have sufficient rational grounds to adopt it despite any remaining anomalies it may possess*.

In sum, to deny the validity and robustness of my theory, one would have to disregard all the empirical and conceptual problems it resolves. Yet we cannot do so without offering good reasons, for no rival hypothesis is capable of accounting for all the problems that the Sentinel Sleep Theory resolves. Thus, if we reject my theory, such a rejection will lead us to a considerable loss in scientific efficiency in solving problems. This is because any rival hypothesis (so far proposed for REM sleep) can resolve far fewer empirical and conceptual problems than my theory and, as of this work, possesses a much greater number of anomalous problems.

**S7. How to refute my theory?**

In Section 2, I sought to demonstrate that we must consider many nuances before assuming that a biological theory has been refuted. However, understanding these nuances is not the same as immunizing the theory against refutations. Therefore, in order to demonstrate more clearly that I am not attempting to immunize my theory from refutations, it is useful to present hypothetical refuting cases that would pose a serious challenge to it. These cases will add to the long list of hypotheses I already proposed to test my theory (see the Tables in this article, especially Table 2).

***1. Discovering an animal lineage with bi-hemispheric sleep, highly vulnerable to predation, but that has completely lost REM sleep*.** If phylogenetic studies and sleep analyses were to reveal a lineage of mammals highly vulnerable to predation that demonstrably evolved from ancestors possessing REM sleep but eventually lost it, this would undermine the foundations of my proposal. Such a discovery would directly falsify my claim that REM sleep is a necessary adaptation for any organism that must sleep with both hemispheres. It would demonstrate that the “insurmountable vulnerability” of N-REM sleep is, in fact, surmountable, and that REM sleep is an optional evolutionary solution rather than an adaptive necessity. Thus, it would erode the pillar of evolutionary necessity upon which my theory rests. Especially if no other compensatory mechanism were evident, such a discovery would constitute a significant and fatal anomaly.
 ***2. Discovering abundant REM sleep in animals that exhibit strictly uni-hemispheric sleep and that are evolutionarily more recent*.** For example, if we were to discover that cetaceans, in fact, possess a form of REM sleep that has thus far eluded our ability to observe and measure it, and that this REM sleep is abundant, then my theory would face a serious challenge. (This is why part of my mission with this work is also to foster further empirical research on cetaceans based on the conclusions I presented here.)

***3. Demonstrating, through robust and independent research, that the primary function of REM sleep is anything other than the sentinel function*.** This would challenge my claim that the sentinel function is *primary*. In such a case, the high energetic cost of REM sleep would be justified by this other function, relegating the sentinel function to a secondary role. It should be noted that such a discovery would destabilize a considerable portion of the structure of my theory, since it is entirely grounded in the idea that REM sleep and its various physiological mechanisms evolved primarily to address the vulnerability of N-REM sleep.

***4. Demonstrating that vigilance and alertness can be dissociated from REM sleep*.** If the characteristics and behaviors of alertness, vigilance, and readiness are not intrinsic to REM sleep but rather components that can be dissociated from it, then REM sleep could exist without being tied to a sentinel function. In that case, vigilance and attention to threats could not be its primary and defining biological function, and my entire theory—which links the physiological characteristics of REM sleep to the sentinel function—would be invalidated.

***5. Identifying a highly efficient vigilance mechanism associated with N-REM sleep*.** This would dismantle the fundamental problem that my theory proposes to solve. If N-REM sleep already possessed a highly efficient and energetically cheaper sentinel mechanism, then the evolution of a second, far more costly system (REM sleep) to perform the same function would violate the principle of evolutionary parsimony. REM sleep would become a redundant mechanism and would possibly imply another primary function, thereby refuting my arguments that REM sleep evolved as a countermeasure to the vulnerability of N-REM sleep.

**References**

Abraham, W.C., Dragunow, M., Tate, W.P., 1991. The role of immediate early genes in the stabilization of long-term potentiation. Mol. Neurobiol. 5, 297-314. https://doi.org/10.1007/BF02935553.

Abrams, P.A., 1992. Adaptive foraging by predators as a cause of predator-prey cycles. Evol. Ecol. 6, 56-72.

Abrams, P.A., 2000. The evolution of predator-prey interactions: theory and evidence. Annu. Rev. Ecol. Syst. 31, 79-105.

Allison, T., Cicchetti, D.V., 1976. Sleep in mammals: ecological and constitutional correlates. Science (New York, N.Y.), 194(4266), 732–734. https://doi.org/10.1126/science.982039

Amici, R., Cerri, M., Ocampo-Garcés, A., Baracchi, F., Dentico, D., Jones, C.A., Luppi, M., Perez, E., Parmeggiani, P.L., Zamboni, G., 2008. Cold exposure and sleep in the rat: REM sleep homeostasis and body size. Sleep, 31, 708-715. https://doi.org/10.1093/sleep/31.5.708.

Amici, R., Zamboni, G., Perez, E., Jones, C.A., Parmeggiani, P.L., 1998. The influence of a heavy thermal load on REM sleep in the rat. Brain research, 781, 252-258. https://doi.org/10.1016/s0006-8993(97)01242-0.

Aston-Jones, G., Bloom, F.E., 1981. Activity of norepinephrine-containing locus coeruleus neurons in behaving rats anticipates fluctuations in the sleep-waking cycle. J. Neurosci. 1, 876-886. https://doi.org/10.1523/JNEUROSCI.01-08-00876.1981.

Atienza, M., Cantero, J.L., Escera, C., 2001. Auditory information processing during human sleep as revealed by event-related brain potentials. Clin. Neurophysiol. 112, 2031-2045. https://doi.org/10.1016/s1388-2457(01)00650-2.

Bahrami, S., Drabløs, F., 2016. Gene regulation in the immediate-early response process. Advances in biological regulation 62, 37-49. https://doi.org/10.1016/j.jbior.2016.05.001.

Bass, D.I., Manns, J.R., 2015. Memory-enhancing amygdala stimulation elicits gamma synchrony in the hippocampus. Behav. Neurosci. 129, 244-256. https://doi.org/10.1037/bne0000052.

Bass, D.I., Nizam, Z.G., Partain, K.N., Wang, A., Manns, J.R., 2014. Amygdala-mediated enhancement of memory for specific events depends on the hippocampus. Neurobiol. Learn. Mem. 107, 37-41. https://doi.org/10.1016/j.nlm.2013.10.020.

Bass, D.I., Partain, K.N., Manns, J.R., 2012. Event-specific enhancement of memory via brief electrical stimulation to the basolateral complex of the amygdala in rats. Behav. Neurosci. 126, 204-208. https://doi.org/10.1037/a0026462.

Bear, M.F., Connors, B.W., Paradiso, M.A., 2016. Neuroscience: Exploring the Brain, fourth ed. Wolters Kluwer, Philadelphia.

Becks, L., Ellner, S.P., Jones, L.E., Hairston, N.G., Jr, 2012. The functional genomics of an eco-evolutionary feedback loop: linking gene expression, trait evolution, and community dynamics. Ecol. Lett. 15, 492-501. https://doi.org/10.1111/j.1461-0248.2012.01763.x.

Bergel, A., Deffieux, T., Demené, C., Tanter, M., Cohen, I., 2018. Local hippocampal fast gamma rhythms precede brain-wide hyperemic patterns during spontaneous rodent REM sleep. Nat. Commun. 9, 5364. https://doi.org/10.1038/s41467-018-07752-3.

Bisaz, R., Travaglia, A., Alberini, C.M., 2014. The neurobiological bases of memory formation: from physiological conditions to psychopathology. Psychopathology, 47, 347-356. https://doi.org/10.1159/000363702.

Bisschops, L.L., Hoedemaekers, C.W., Simons, K.S., van der Hoeven, J.G., 2010. Preserved metabolic coupling and cerebrovascular reactivity during mild hypothermia after cardiac arrest. CCM, 38, 1542-1547. https://doi.org/10.1097/CCM.0b013e3181e2cc1e.

Bohannon, J. (2006). When Predators Attack: Carnivore-on-carnivore violence keeps competition in check. Science.org. Available at: https://www.science.org/content/article/when-predators-attack (accessed 26 Aug, 2025).

Boutrel, B., Monaca, C., Hen, R., Hamon, M., Adrien, J., 2002. Involvement of 5-HT1A receptors in homeostatic and stress-induced adaptive regulations of paradoxical sleep: studies in 5-HT1A knock-out mice. J. Neurosci. 22, 4686-4692. https://doi.org/10.1523/JNEUROSCI.22-11-04686.2002.

Brinkman, J. E., Reddy, V., Sharma, S., 2023. Physiology of Sleep. StatPearls, StatPearls Publishing. https://www.ncbi.nlm.nih.gov/books/NBK482512/ (accessed 26 Aug, 2025).

Brooks, P.L., Peever, J., 2016. A Temporally Controlled Inhibitory Drive Coordinates Twitch Movements during REM Sleep. Curr. Biol., 26, 1177-1182. https://doi.org/10.1016/j.cub.2016.03.013

Brown, R.E., Basheer, R., McKenna, J.T., Strecker, R.E., McCarley, R.W., 2012. Control of sleep and wakefulness. Physiol. Rev. 92, 1087-1187. https://doi.org/10.1152/physrev.00032.2011.

Burak, M.K., Monk, J.D., Schmitz, O.J., 2018. Eco-Evolutionary Dynamics: The Predator-Prey Adaptive Play and the Ecological Theater. Yale J. Biol. Med. 91, 481-489.

Campbell, S.S., Tobler, I., 1984. Animal sleep: a review of sleep duration across phylogeny. Neurosci. Biobehav. Rev. 8, 269-300. https://doi.org/10.1016/0149-7634(84)90054-x.

Capellini, I., Barton, R.A., McNamara, P., Preston, B.T., Nunn, C.L., 2008. Phylogenetic analysis of the ecology and evolution of mammalian sleep. Evol.; int. j. org. evol. 62, 1764-1776. https://doi.org/10.1111/j.1558-5646.2008.00392.x.

Cardis, R., Lecci, S., Fernandez, L.M., Osorio-Forero, A., Chu Sin Chung, P., Fulda, S., Decosterd, I., Lüthi, A., 2021. Cortico-autonomic local arousals and heightened somatosensory arousability during NREMS of mice in neuropathic pain. eLife 10, e65835. https://doi.org/10.7554/eLife.65835.

Carter, M.E., Yizhar, O., Chikahisa, S., Nguyen, H., Adamantidis, A., Nishino, S., Deisseroth, K., de Lecea, L., 2010. Tuning arousal with optogenetic modulation of locus coeruleus neurons. Nat. Neurosci. 13, 1526-1533. https://doi.org/10.1038/nn.2682.

Cerri, M., Ocampo-Garces, A., Amici, R., Baracchi, F., Capitani, P., Jones, C.A., Luppi, M., Perez, E., Parmeggiani, P.L., Zamboni, G., 2005. Cold exposure and sleep in the rat: effects on sleep architecture and the electroencephalogram. Sleep, 28, 694-705. https://doi.org/10.1093/sleep/28.6.694.

Chamorro, R., Algarín, C., Garrido, M., Causa, L., Held, C., Lozoff, B., Peirano, P., 2014. Night time sleep macrostructure is altered in otherwise healthy 10-year-old overweight children. IJO 38, 1120-1125. https://doi.org/10.1038/ijo.2013.238.

Chand, T., Alizadeh, S., Jamalabadi, H., Herrmann, L., Krylova, M., Surova, G., van der Meer, J., Wagner, G., Engert, V., Walter, M., 2021. EEG revealed improved vigilance regulation after stress exposure under Nx4 - A randomized, placebo-controlled, double-blind, cross-over trial. IBRO Neurosci. Rep. 11, 175-182. https://doi.org/10.1016/j.ibneur.2021.09.002. Erratum in: IBRO Neurosci. Rep. 12, 81.

Cortez, M., Ellner, S.P., 2010. Understanding rapid evolution in predator‐prey interactions using the theory of fast‐slow dynamical systems. Am. Nat. 176, E109-E127. https://doi.org/10.1086/656485.

Crick, F., Mitchison, G., 1983. The function of dream sleep. Nature 304, 111-114. https://doi.org/10.1038/304111a0.

Croughwell, N., Smith, L.R., Quill, T., Newman, M., Greeley, W., Kern, F., Lu, J., Reves, J.G., 1992. The effect of temperature on cerebral metabolism and blood flow in adults during cardiopulmonary bypass. J. Thorac. Cardiovasc. Surg. 103, 549-554.

Damasio, A.R., 1996. The somatic marker hypothesis and the possible functions of the prefrontal cortex. Philos. Trans. R. Soc. Lond. B Biol. Sci., 351, 1413-1420. https://doi.org/10.1098/rstb.1996.0125.

Damasio, A.R., 2003. Looking for Spinoza: Joy, Sorrow, and the Feeling Brain. Houghton Mifflin Harcourt, New York.

Damasio, A.R., 2012. Self Comes to Mind: Constructing the Conscious Brain. Vintage Books, New York.

Damasio, A.R., 2019. The Strange Order of Things: Life, Feeling, and the Making of Cultures. Vintage Books, New York.

Darwin, C.R., 1859. On the origin of species by means of natural selection, or the preservation of favoured races in the struggle for life. John Murray, London.

Datta, S., Siwek, D.F., 2002. Single cell activity patterns of pedunculopontine tegmentum neurons across the sleep-wake cycle in the freely moving rats. J. Neurosci. Res. 70, 611-621. https://doi.org/10.1002/jnr.10405.

Dauvilliers, Y., Arnulf, I., Mignot, E., 2007. Narcolepsy with cataplexy. Lancet (London, England) 369, 499-511. https://doi.org/10.1016/S0140-6736(07)60237-2.

Davis, S., Bozon, B., Laroche, S., 2003. How necessary is the activation of the immediate early gene zif268 in synaptic plasticity and learning?. Behav. Brain Res. 142, 17-30. https://doi.org/10.1016/s0166-4328(02)00421-7.

Dawkins, R., 1997. Climbing Mount Improbable. W. W. Norton & Company, New York.

Dawkins, R., 2015. The Blind Watchmaker: Why the Evidence of Evolution Reveals a Universe Without Design. W. W. Norton & Company, New York.

Decker, M.J., Rye, D.B., Lee, S.Y., Strohl, K.P., 2010. Paradoxical sleep suppresses immediate early gene expression in the rodent suprachiasmatic nuclei. Front. Neurol. 1, 122. https://doi.org/10.3389/fneur.2010.00122.

Denoyer, M., Sallanon, M., Buda, C., Delhomme, G., Dittmar, A., Jouvet, M., 1991. The posterior hypothalamus is responsible for the increase of brain temperature during paradoxical sleep. Exp. Brain Res. 84, 326-334. https://doi.org/10.1007/BF00231453

Desseilles, M., Dang-Vu, T.T., Sterpenich, V., Schwartz, S., 2011. Cognitive and emotional processes during dreaming: a neuroimaging view. Conscious. Cogn. 20, 998-1008. https://doi.org/10.1016/j.concog.2010.10.005.

Deurveilher, S. Semba, K., 2011. Basal forebrain regulation of cortical activity and sleep-wake states: Roles of cholinergic and non-cholinergic neurons. Sleep and Biol. Rhythms 9, 65-70. https://doi.org/10.1111/j.1479-8425.2010.00465.x.

de Feijter, M., Katimertzoglou, A., Tiemensma, J., Ikram, M.A., Luik, A.I., 2022. Polysomnography-estimated sleep and the negative feedback loop of the hypothalamic-pituitary-adrenal (HPA) axis. Psychoneuroendocrinology 141, 105749. https://doi.org/10.1016/j.psyneuen.2022.105749.

De Luca, R., Nardone, S., Grace, K.P., Venner, A., Cristofolini, M., Bandaru, S.S., Sohn, L.T., Kong, D., Mochizuki, T., Viberti, B., Zhu, L., Zito, A., Scammell, T.E., Saper, C.B., Lowell, B.B., Fuller, P.M., Arrigoni, E., 2022. Orexin neurons inhibit sleep to promote arousal. Nat. Commun. 13, 4163. https://doi.org/10.1038/s41467-022-31591-y.

de Lecea, L., Huerta, R., 2014. Hypocretin (orexin) regulation of sleep-to-wake transitions. Front. pharmacol. 5, 16. https://doi.org/10.3389/fphar.2014.00016.

Dragoi, G., Carpi, D., Recce, M., Csicsvari, J., Buzsáki, G., 1999. Interactions between hippocampus and medial septum during sharp waves and theta oscillation in the behaving rat. J. Neurosci. 19, 6191-6199. https://doi.org/10.1523/JNEUROSCI.19-14-06191.1999.

Eagleman, D.M., Vaughn, D.A., 2021. The Defensive Activation Theory: REM sleep as a mechanism to prevent takeover of the visual cortex. Front. Neurosci. 15, 632853. https://doi.org/10.3389/fnins.2021.632853.

Ebbinghaus, H., 1913. Retention as a function of the number of repetitions. In: Ebbinghaus H., Ruger H.A., Bussenius C.E. (Eds. & Trans.), Memory: A contribution to experimental psychology. Teachers College Press, New York, pp. 52-61. https://psycnet.apa.org/doi/10.1037/10011-006

Ermis, U., Krakow, K., Voss, U., 2010. Arousal thresholds during human tonic and phasic REM sleep. J. Sleep Res. 19, 400-406. https://doi.org/10.1111/j.1365-2869.2010.00831.x.

España, R.A., Scammell, T.E., 2011. Sleep neurobiology from a clinical perspective. Sleep 34, 845-858. https://doi.org/10.5665/SLEEP.1112.

Estabrooke, I.V., McCarthy, M.T., Ko, E., Chou, T.C., Chemelli, R.M., Yanagisawa, M., Saper, C.B., Scammell, T.E., 2001. Fos expression in orexin neurons varies with behavioral state. J. Neurosci. 21, 1656-1662. https://doi.org/10.1523/JNEUROSCI.21-05-01656.2001.

Fang, J., Sanborn, C.K., Renegar, K.B., Majde, J.A., Krueger, J.M., 1995. Influenza viral infections enhance sleep in mice. Proc. Soc. Exp. Biol. Med. 210, 242-252. https://doi.org/10.3181/00379727-210-43945.

Feng, H., Wen, S.Y., Qiao, Q.C., Pang, Y.J., Wang, S.Y., Li, H.Y., Cai, J., Zhang, K.X., Chen, J., Hu, Z.A., Luo, F.L., Wang, G.Z., Yang, N., Zhang, J., 2020. Orexin signaling modulates synchronized excitation in the sublaterodorsal tegmental nucleus to stabilize REM sleep. Nat. Commun. 11, 3661. https://doi.org/10.1038/s41467-020-17401-3.

Fogwe, L.A., Reddy, V., Mesfin, F.B., 2023. Neuroanatomy, Hippocampus. In: StatPearls. StatPearls Publishing.

Foote, S.L., Aston-Jones, G., Bloom, F.E., 1980. Impulse activity of locus coeruleus neurons in awake rats and monkeys is a function of sensory stimulation and arousal. PNAS 77, 3033-3037. https://doi.org/10.1073/pnas.77.5.3033.

Forester, G., Kroneisen, M., Erdfelder, E., Kamp, S.M., 2020. Adaptive Memory: Independent Effects of Survival Processing and Reward Motivation on Memory. Front. hum. neurosci. 14, 588100. https://doi.org/10.3389/fnhum.2020.588100.

Gazzaniga, M., Heatherton, T., Halpern, D., 2016. Psychological Science, fifth ed. W. W. Norton & Company, New York.

Geva‑Sagiv, M., Mankin, E.A., Eliashiv, D., Epstein, S., Cherry, N., Kalender, G., Tchemodanov, N., Nir, Y., Fried, I., 2023. Augmenting hippocampal-prefrontal neuronal synchrony during sleep enhances memory consolidation in humans. Nat. Neurosci. 26, 1100-1110. https://doi.org/10.1038/s41593-023-01324-5.

Giamarellos-Bourboulis, E.J., Raftogiannis, M., 2012. The immune response to severe bacterial infections: consequences for therapy. Expert review of anti-infective therapy, 10, 369–380. https://doi.org/10.1586/eri.12.2

Groch, S., Wilhelm, I., Diekelmann, S., Born, J., 2013. The role of REM sleep in the processing of emotional memories: evidence from behavior and event-related potentials. Neurobiology of learning and memory, 99, 1–9. https://doi.org/10.1016/j.nlm.2012.10.006

Han, H., Seong, M.J., Hyeon, J., Joo, E., Oh, J., 2024. Classification and automatic scoring of arousal intensity during sleep stages using machine learning. Sci. Rep. 14, 5983. https://doi.org/10.1038/s41598-023-50653-9.

Hebb, D.O., 1961. Distinctive features of learning in the higher animal. In: Delafresnaye, J.F. (Ed.), Brain mechanisms and learning. Blackwell, Oxford, pp. 37-46.

Horner, R.L., Sanford, L.D., Annis, D., Pack, A.I., Morrison, A.R., 1997a. Serotonin at the laterodorsal tegmental nucleus suppresses rapid-eye-movement sleep in freely behaving rats. J. Neurosci. 17, 7541-7552. https://doi.org/10.1523/JNEUROSCI.17-19-07541.1997.

Horner, R.L., Sanford, L.D., Pack, A.I., Morrison, A.R., 1997b. Activation of a distinct arousal state immediately after spontaneous awakening from sleep. Brain Res. 778, 127-134. https://doi.org/10.1016/s0006-8993(97)01045-7.

Ibayashi, S., Takano, K., Ooboshi, H., Kitazono, T., Sadoshima, S., Fujishima, M., 2000. Effect of selective brain hypothermia on regional cerebral blood flow and tissue metabolism using brain thermo-regulator in spontaneously hypertensive rats. Neurochem. Res. 25, 369-375. https://doi.org/10.1023/a:1007593004806.

Imeri, L., Opp, M.R., 2009. How (and why) the immune system makes us sleep. Nature reviews. Neuroscience, 10, 199-210. https://doi.org/10.1038/nrn2576.

Ito, H., Fukatsu, N., Rahaman, S.M., Mukai, Y., Izawa, S., Ono, D., Kilduff, T.S., Yamanaka, A., 2023. Deficiency of orexin signaling during sleep is involved in abnormal REM sleep architecture in narcolepsy. PNAS 120, e2301951120. https://doi.org/10.1073/pnas.2301951120.

Izquierdo, I., 2018. Memória [Memory]. Artmed, Porto Alegre.

Kahneman, D., 2011. Thinking, Fast and Slow. Farrar, Straus and Giroux, New York.

Kandel, E.R., Dudai, Y., Mayford, M.R., 2014. The molecular and systems biology of memory. Cell, 157, 163-186. https://doi.org/10.1016/j.cell.2014.03.001

Kandel, E.R., Koester, J.D., Mack, S.H., Siegelbaum, S.A. (Eds.), 2021. Principles of neural science, sixth ed. McGraw-Hill, New York.

Kandel, E.R., Schwartz, J.H., Jessell, T.M., Siegelbaum, S.A., Hudspeth, A.J. (Eds.), 2013. Principles of neural science, fifth ed. McGraw-Hill, New York.

Kingsley, E., 2024. 12 Animals Bold and Brave Enough to Take on a Lion. A-Z-Animals. Available at: https://a-z-animals.com/animals/lion/facts-lion/lion-threats/.

Kitamura, E., Kawasaki, Y., Kasai, T., Midorikawa, I., Shiroshita, N., Kawana, F., Ogasawara, E., Kitade, M., Itakura, A., Koikawa, N., Matsuda, T., 2021. The relationship between body composition and sleep architecture in athletes. Sleep Med. 87, 92-96. https://doi.org/10.1016/j.sleep.2021.08.028.

Kjaerby, C., Andersen, M., Hauglund, N., Untiet, V., Dall, C., Sigurdsson, B., Ding, F., Feng, J., Li, Y., Weikop, P., Hirase, H., Nedergaard, M., 2022. Memory-enhancing properties of sleep depend on the oscillatory amplitude of norepinephrine. Nat. Neurosci. 25, 1059-1070. https://doi.org/10.1038/s41593-022-01102-9.

Kollar, E.J., Pasnau, R.O., Rubin, R.T., Naitoh, P., Slater, G.G., Kales, A., 1969. Psychological, psychophysiological, and biochemical correlates of prolonged sleep deprivation. Am. J. Psychiatry 126, 488-497. https://doi.org/10.1176/ajp.126.4.488.

Koshmanova, E., Berger, A., Beckers, E., Campbell, I., Mortazavi, N., Sharifpour, R., Paparella, I., Balda, F., Berthomier, C., Degueldre, C., Salmon, E., Lamalle, L., Bastin, C., Van Egroo, M., Phillips, C., Maquet, P., Collette, F., Muto, V., Chylinski, D., Jacobs, H.I., … Vandewalle, G., 2023. Locus coeruleus activity while awake is associated with REM sleep quality in older individuals. JCI insight 8, e172008. https://doi.org/10.1172/jci.insight.172008.

Krueger, J.M., Majde, J.A., 1994. Microbial products and cytokines in sleep and fever regulation. Critical reviews in immunology, 14, 355-379. https://doi.org/10.1615/critrevimmunol.v14.i3-4.70.

Krueger, J.M., Pappenheimer, J.R., Karnovsky, M.L., 1982. Sleep-promoting effects of muramyl peptides. Proceedings of the National Academy of Sciences of the United States of America, 79, 6102–6106. https://doi.org/10.1073/pnas.79.19.6102

Laudan, L., 1977. Progress and Its Problems: Towards a Theory of Scientific Growth. Routledge & Kegan Paul, London.

Lee, A. K., Wilson, M. A., 2002. Memory of sequential experience in the hippocampus during slow wave sleep. Neuron, 36, 1183-1194. https://doi.org/10.1016/s0896-6273(02)01096-6

Lee, M.G., Hassani, O.K., Jones, B.E., 2005. Discharge of identified orexin/hypocretin neurons across the sleep-waking cycle. J. Neurosci. 25, 6716-6720. https://doi.org/10.1523/JNEUROSCI.1887-05.2005.

Le Bon, O., Staner, L., Rivelli, S.K., Hoffmann, G., Pelc, I., Linkowski, P., 2002. Correlations using the NREM-REM sleep cycle frequency support distinct regulation mechanisms for REM and NREM sleep. J. Appl. Physiol. (Bethesda, Md. : 1985) 93, 141-146. https://doi.org/10.1152/japplphysiol.00917.2001.

Le Bon, O. (2020). Relationships between REM and NREM in the NREM-REM sleep cycle: a review on competing concepts. Sleep Med. 70, 6-16. https://doi.org/10.1016/j.sleep.2020.02.004.

Lesku, J.A., Roth, T.C., 2nd, Amlaner, C.J., Lima, S.L., 2006. A phylogenetic analysis of sleep architecture in mammals: the integration of anatomy, physiology, and ecology. Am. Nat. 168, 441-453. https://doi.org/10.1086/506973.

Lima, S.L., Rattenborg, N.C., Lesku, J.A., Amlaner, C.J., 2005. Sleeping under the risk of predation. Anim. Behav. 70, 723-736.

Liu, X., Forbes, E.E., Ryan, N.D., Rofey, D., Hannon, T.S., Dahl, R.E., 2008. Rapid eye movement sleep in relation to overweight in children and adolescents. Arch. Gen. Psychiatry 65, 924-932. https://doi.org/10.1001/archpsyc.65.8.924.

Li, W., Ma, L., Yang, G., Gan, W.B., 2017. REM sleep selectively prunes and maintains new synapses in development and learning. Nature neuroscience, 20, 427–437. https://doi.org/10.1038/nn.4479

Luppi, P.H., Chancel, A., Malcey, J., Cabrera, S., Fort, P., Maciel, R.M., 2024. Which structure generates paradoxical (REM) sleep: The brainstem, the hypothalamus, the amygdala or the cortex?. Sleep Med. Rev., 74, 101907. https://doi.org/10.1016/j.smrv.2024.101907.

Magalhães, S., Janssen, A., Montserrat, M., Sabelis, M. W., 2005. Prey attack and predators defend: counterattacking prey trigger parental care in predators. Proc. Biol. Sci. 272, 1929-1933. https://doi.org/10.1098/rspb.2005.3127

Martin, J.M., Andriano, D.W., Mota, N.B., Mota-Rolim, S.A., Araújo, J.F., Solms, M., Ribeiro, S., 2020. Structural differences between REM and non-REM dream reports assessed by graph analysis. PloS one 15, e0228903. https://doi.org/10.1371/journal.pone.0228903.

Martin, S.J., Grimwood, P.D., Morris, R.G., 2000. Synaptic plasticity and memory: an evaluation of the hypothesis. Annu. Rev. Neurosci. 23, 649-711. https://doi.org/10.1146/annurev.neuro.23.1.649.

Mavanji, V., Perez-Leighton, C.E., Kotz, C.M., Billington, C.J., Parthasarathy, S., Sinton, C.M., Teske, J.A., 2015. Promotion of Wakefulness and Energy Expenditure by Orexin-A in the Ventrolateral Preoptic Area. Sleep 38, 1361-1370. https://doi.org/10.5665/sleep.4970.

Mayr, E., 1982. The Growth of Biological Thought: Diversity, Evolution, and Inheritance. Harvard University Press, Cambridge.

Mayr, E., 2001. This is Biology: The Science of the Living World. Harvard University Press, Cambridge.

Mileykovskiy, B.Y., Kiyashchenko, L.I., Siegel, J.M., 2005. Behavioral correlates of activity in identified hypocretin/orexin neurons. Neuron 46, 787-798. https://doi.org/10.1016/j.neuron.2005.04.035.

Mochizuki, T., Arrigoni, E., Marcus, J.N., Clark, E.L., Yamamoto, M., Honer, M., Borroni, E., Lowell, B.B., Elmquist, J.K., Scammell, T.E., 2011. Orexin receptor 2 expression in the posterior hypothalamus rescues sleepiness in narcoleptic mice. PNAS 108, 4471-4476. https://doi.org/10.1073/pnas.1012456108.

Mogavero, M.P., Godos, J., Grosso, G., Caraci, F., Ferri, R., 2023. Rethinking the Role of Orexin in the Regulation of REM Sleep and Appetite. Nutrients 15, 3679. https://doi.org/10.3390/nu15173679.

Monti, J.M., Jantos, H., 2008. The roles of dopamine and serotonin, and of their receptors, in regulating sleep and waking. Prog. Brain Res. 172, 625-646. https://doi.org/10.1016/S0079-6123(08)00929-1.

Montgomery, S.M., Sirota, A., Buzsáki, G., 2008. Theta and gamma coordination of hippocampal networks during waking and rapid eye movement sleep. J. Neurosci. 28, 6731-6741. https://doi.org/10.1523/JNEUROSCI.1227-08.2008.

Moruzzi, G., Eccles, J.C., 1966. Brain and conscious experience. Adv. Neurol. 77, 181-192.

Moyne, M., Legendre, G., Arnal, L., Kumar, S., Sterpenich, V., Seeck, M., Grandjean, D., Schwartz, S., Vuilleumier, P., Domínguez-Borràs, J., 2022. Brain reactivity to emotion persists in NREM sleep and is associated with individual dream recall. Cereb. Cortex Commun. 3, tgac003. https://doi.org/10.1093/texcom/tgac003.

Mrozek, S., Vardon, F., Geeraerts, T., 2012. Brain temperature: physiology and pathophysiology after brain injury. Anesthesiol. Res. Pract. 989487. https://doi.org/10.1155/2012/989487.

Myllymäki, T., Kyröläinen, H., Savolainen, K., Hokka, L., Jakonen, R., Juuti, T., Martinmäki, K., Kaartinen, J., Kinnunen, M.L., Rusko, H., 2011. Effects of vigorous late-night exercise on sleep quality and cardiac autonomic activity. J. Sleep Res. 20, 146-153. https://doi.org/10.1111/j.1365-2869.2010.00874.x.

Nádasdy, Z., Hirase, H., Czurkó, A., Csicsvari, J., Buzsáki, G., 1999. Replay and time compression of recurring spike sequences in the hippocampus. J. Neurosci., 19, 9497-9507. https://doi.org/10.1523/JNEUROSCI.19-21-09497.1999

Nishida, M., Pearsall, J., Buckner, R.L., Walker, M.P., 2009. REM sleep, prefrontal theta, and the consolidation of human emotional memory. Cereb. Cortex 19, 1158-1166. https://doi.org/10.1093/cercor/bhn155.

Nolan, M.F., 2023. Memory consolidation: Building influence over the entorhinal cortex. Curr. Biol. 33, R1160-R1162. https://doi.org/10.1016/j.cub.2023.09.072.

Okawa, H., Morokuma, S., Maehara, K., Arata, A., Ohmura, Y., Horinouchi, T., Konishi, Y., Kato, K., 2017. Eye movement activity in normal human fetuses between 24 and 39 weeks of gestation. PloS one 12, e0178722. https://doi.org/10.1371/journal.pone.0178722.

Oken, B.S., Salinsky, M.C., Elsas, S.M., 2006. Vigilance, alertness, or sustained attention: physiological basis and measurement. Clin. Neurophysiol. 117, 1885-1901. https://doi.org/10.1016/j.clinph.2006.01.017.

Okuno, H., 2011. Regulation and function of immediate-early genes in the brain: beyond neuronal activity markers. Neurosci. Res. 69, 175-186. https://doi.org/10.1016/j.neures.2010.12.007.

Ono, D., Yamanaka, A., 2017. Hypothalamic regulation of the sleep/wake cycle. Neurosci. Res. 118, 74-81. https://doi.org/10.1016/j.neures.2017.03.013.

Opp, M.R., 2005. Cytokines and sleep. Sleep medicine reviews, 9, 355-364. https://doi.org/10.1016/j.smrv.2005.01.002.

Osorio-Forero, A., Cardis, R., Vantomme, G., Guillaume-Gentil, A., Katsioudi, G., Devenoges, C., Fernandez, L.M.J., Lüthi, A., 2021. Noradrenergic circuit control of non-REM sleep substates. Curr. Biol. 31, 5009-5023.e7. https://doi.org/10.1016/j.cub.2021.09.041.

Osorio-Forero, A., Cherrad, N., Banterle, L., Fernandez, L.M.J., Lüthi, A., 2022. When the Locus Coeruleus Speaks Up in Sleep: Recent Insights, Emerging Perspectives. Int. J. Mol. Sci. 23, 5028. https://doi.org/10.3390/ijms23095028.

O’Leary, J.D., Bruckner, R., Autore, L., Ryan, T.J., 2024. Natural forgetting reversibly modulates engram expression. eLife 12, RP92860. https://doi.org/10.7554/eLife.92860.

Parmeggiani, P.L., 2007. REM sleep related increase in brain temperature: a physiologic problem. Arch. Ital. Biol. 145, 13-21.

Pastukhov, Y.F., Ekimova, I.V., 2012. The Thermophysiology of Paradoxical Sleep. Neurosci. Behav. Physi. 42, 933-947. https://doi.org/10.1007/s11055-012-9660-5.

Payne, J.D., Stickgold, R., Swanberg, K., Kensinger, E.A., 2008. Sleep preferentially enhances memory for emotional components of scenes. Psychol. Sci. 19, 781-788. https://doi.org/10.1111/j.1467-9280.2008.02157.x.

Pignatelli, M., Ryan, T.J., Roy, D.S., Lovett, C., Smith, L.M., Muralidhar, S., Tonegawa, S., 2019. Engram Cell Excitability State Determines the Efficacy of Memory Retrieval. Neuron, 101, 274-284.e5. https://doi.org/10.1016/j.neuron.2018.11.029

Pilon, M., Desautels, A., Montplaisir, J., Zadra, A., 2012. Auditory arousal responses and thresholds during REM and NREM sleep of sleepwalkers and controls. Sleep Med. 13, 490-495. https://doi.org/10.1016/j.sleep.2011.10.031.

Poe, G.R., Foote, S., Eschenko, O., Johansen, J.P., Bouret, S., Aston-Jones, G., Harley, C.W., Manahan-Vaughan, D., Weinshenker, D., Valentino, R., Berridge, C., Chandler, D.J., Waterhouse, B., Sara, S.J., 2020. Locus coeruleus: a new look at the blue spot. Nat. Rev. Neurosci. 21, 644-659. https://doi.org/10.1038/s41583-020-0360-9.

Poe, G.R., Nitz, D.A., McNaughton, B.L., Barnes, C.A., 2000. Experience-dependent phase-reversal of hippocampal neuron firing during REM sleep. Brain Res. 855, 176-180. https://doi.org/10.1016/s0006-8993(99)02310-0.

Pollmächer, T., Schreiber, W., Gudewill, S., Vedder, H., Fassbender, K., Wiedemann, K., Trachsel, L., Galanos, C., Holsboer, F., 1993. Influence of endotoxin on nocturnal sleep in humans. The American journal of physiology, 264, R1077-R1083. https://doi.org/10.1152/ajpregu.1993.264.6.R1077.

Pompeiano, M., Cirelli, C., Arrighi, P., Tononi, G., 1995. c-Fos expression during wakefulness and sleep. Neurophysiol. Clin. (NCCN) 25, 329-341. https://doi.org/10.1016/0987-7053(96)84906-9.

Pompeiano, M., Cirelli, C., Ronca-Testoni, S.P., Tononi, G., 1997. NGFI-A expression in the rat brain after sleep deprivation. Mol. Brain Res. 46, 143-153. https://doi.org/10.1016/S0169-328X(96)00295-1.

Pompeiano, M., Cirelli, C., Tononi, G., 1992. Effects of sleep deprivation on fos-like immunoreactivity in the rat brain. Arch. Ital. Biol. 130, 325-335.

Pompeiano, M., Cirelli, C., Tononi, G., 1994. Immediate-early genes in spontaneous wakefulness and sleep: expression of c-fos and NGFI-A mRNA and protein. J. Sleep Res. 3, 80-96. https://doi.org/10.1111/j.1365-2869.1994.tb00111.x.

Post, D.M., Palkovacs, E.P., 2009. Eco-evolutionary feedbacks in community and ecosystem ecology: interactions between the ecological theatre and the evolutionary play. Philos. Trans. R. Soc. B, Biol. Sci. 364, 1629-1640. https://doi.org/10.1098/rstb.2009.0012.

Price, L.J., Kremen, I., 1980. Variations in behavioral response threshold within the REM period of human sleep. Psychophysiology 17, 133-140. https://doi.org/10.1111/j.1469-8986.1980.tb00125.x.

Rattenborg, N.C., Martinez-Gonzalez, D., Roth, T.C., 2nd, Pravosudov, V.V., 2011. Hippocampal memory consolidation during sleep: a comparison of mammals and birds. Biol. Rev. Camb. Philos. Soc. 86, 658-691. https://doi.org/10.1111/j.1469-185X.2010.00165.x.

Ribeiro, S., Goyal, V., Mello, C.V., Pavlides, C., 1999. Brain gene expression during REM sleep depends on prior waking experience. Learn. Mem. 6, 500-508. https://doi.org/10.1101/lm.6.5.500.

Ribeiro, S., 2021. The Oracle of Night: The History and Science of Dreams. Pantheon, New York.

Ross, J.A., Van Bockstaele, E.J., 2021. The Locus Coeruleus- Norepinephrine System in Stress and Arousal: Unraveling Historical, Current, and Future Perspectives. Front. Psychiatry 11, 601519. https://doi.org/10.3389/fpsyt.2020.601519.

Ryan, T.J., Frankland, P.W., 2022. Forgetting as a form of adaptive engram cell plasticity. Nat. Rev. Neurosci., 23, 173-186. https://doi.org/10.1038/s41583-021-00548-3

Sallinen, M., Kaartinen, J., Lyytinen, H., 1996. Processing of auditory stimuli during tonic and phasic periods of REM sleep as revealed by event‐related brain potentials. J. Sleep Res., 5, 220-228.

Sasaki, K., Suzuki, M., Mieda, M., Tsujino, N., Roth, B., Sakurai, T., 2011. Pharmacogenetic modulation of orexin neurons alters sleep/wakefulness states in mice. PloS one 6, e20360. https://doi.org/10.1371/journal.pone.0020360.

Schwartz, J.R., Roth, T., 2008. Neurophysiology of sleep and wakefulness: basic science and clinical implications. Curr. Neuropharmacol. 6, 367-378. https://doi.org/10.2174/157015908787386050.

Shepherd, J.T., Rusch, N.J., Vanhoutte, P.M., 1983. Effect of cold on the blood vessel wall. Gen. Pharmacol. 14, 61-64. https://doi.org/10.1016/0306-3623(83)90064-2.

Siegel, J.M., 2005. Clues to the functions of mammalian sleep. Nature 437, 1264-1271. https://doi.org/10.1038/nature04285.

Siegel, J., Langley, T.D., 1965. Arousal threshold in the cat as a function of sleep phase and stimulus significance. Experientia 21, 740-741. https://doi.org/10.1007/BF02138511.

Simor, P., van der Wijk, G., Nobili, L., Peigneux, P., 2020. The microstructure of REM sleep: Why phasic and tonic?. Sleep Med. Rev. 52, 101305. https://doi.org/10.1016/j.smrv.2020.101305.

Snyder, F., 1966. Toward an evolutionary theory of dreaming. Am. J. Psychiatry 123, 121-142. https://doi.org/10.1176/ajp.123.2.121.

Solms M. (2000). Dreaming and REM sleep are controlled by different brain mechanisms. Behavioral and brain sciences 23, 843-1121. https://doi.org/10.1017/s0140525x00003988.

Squire, L.R., Genzel, L., Wixted, J.T., Morris, R.G., 2015. Memory consolidation. Cold Spring Harb. Perspect. Biol. 7, a021766. https://doi.org/10.1101/cshperspect.a021766.

Stickgold, R., Walker, M.P., 2007. Sleep-dependent memory consolidation and reconsolidation. Sleep Med. 8, 331-343. https://doi.org/10.1016/j.sleep.2007.03.011.

Swaab, D.F., Dubelaar, E.J., Hofman, M.A., Scherder, E.J., van Someren, E.J., Verwer, R.W., 2002. Brain aging and Alzheimer's disease; use it or lose it. Prog. Brain Res., 138, 343-373. https://doi.org/10.1016/S0079-6123(02)38086-5

Swift, K.M., Gross, B.A., Frazer, M.A., Bauer, D.S., Clark, K.J.D., Vazey, E.M., Aston-Jones, G., Li, Y., Pickering, A.E., Sara, S.J., Poe, G.R., 2018. Abnormal Locus Coeruleus Sleep Activity Alters Sleep Signatures of Memory Consolidation and Impairs Place Cell Stability and Spatial Memory. Curr. Biol. 28, 3599-3609.e4. https://doi.org/10.1016/j.cub.2018.09.054.

Takahara, M., Nittono, H., Hori, T., 2002. Comparison of the event-related potentials between tonic and phasic periods of rapid eye movement sleep. Psychiatry Clin. Neurosci. 56, 257-258. https://doi.org/10.1046/j.1440-1819.2002.00999.x.

Takahashi, K., Kayama, Y., Lin, J.S., Sakai, K., 2010. Locus coeruleus neuronal activity during the sleep-waking cycle in mice. Neuroscience 169, 1115-1126. https://doi.org/10.1016/j.neuroscience.2010.06.009.

Tempesta, D., Socci, V., De Gennaro, L., Ferrara, M., 2018. Sleep and emotional processing. Sleep medicine reviews, 40, 183–195. https://doi.org/10.1016/j.smrv.2017.12.005

Thornton, A., Boogert, N.J., 2019. Animal Cognition: The Benefits of Remembering. Curr. Biol. 29, R324-R327. https://doi.org/10.1016/j.cub.2019.03.055.

Tononi, G., Cirelli, C., 2014. Sleep and the price of plasticity: from synaptic and cellular homeostasis to memory consolidation and integration. Neuron 81, 12-34. https://doi.org/10.1016/j.neuron.2013.12.025.

Toth, L.A., Krueger, J.M., 1988. Alteration of sleep in rabbits by Staphylococcus aureus infection. Infection and immunity, 56, 1785-1791. https://doi.org/10.1128/iai.56.7.1785-1791.1988.

Tseng, Y.T., Zhao, B., Chen, S., Ye, J., Liu, J., Liang, L., Ding, H., Schaefke, B., Yang, Q., Wang, L., Wang, F., Wang, L., 2022. The subthalamic corticotropin-releasing hormone neurons mediate adaptive REM-sleep responses to threat. Neuron 110, 1223-1239.e8. https://doi.org/10.1016/j.neuron.2021.12.033.

Ungurean, G., Barrillot, B., Martinez-Gonzalez, D., Libourel, P.A., Rattenborg, N.C., 2020. Comparative Perspectives that Challenge Brain Warming as the Primary Function of REM Sleep. iScience, 23, 101696. https://doi.org/10.1016/j.isci.2020.101696

van der Helm, E., Walker, M.P., 2011. Sleep and Emotional Memory Processing. Sleep Med. Clin. 6, 31-43. https://doi.org/10.1016/j.jsmc.2010.12.010.

van Hasselt, S.J., Coscia, M., Allocca, G., Vyssotski, A.L., Meerlo, P., 2024. Sleep and Thermoregulation in Birds: Cold Exposure Reduces Brain Temperature but Has Little Influence on Sleep Time and Sleep Architecture in Jackdaws (Coloeus monedula). Biology, 13, 229. https://doi.org/10.3390/biology13040229.

Vazquez, J., Baghdoyan, H.A., 2001. Basal forebrain acetylcholine release during REM sleep is significantly greater than during waking. Am. j. physiol., Regul. integr. comp. physiol. 280, R598-R601. https://doi.org/10.1152/ajpregu.2001.280.2.R598.

Vgontzas, A.N., Chrousos, G.P., 2002. Sleep, the hypothalamic-pituitary-adrenal axis, and cytokines: multiple interactions and disturbances in sleep disorders. Endocrinol. Metab. Clin. North Am. 31, 15-36. https://doi.org/10.1016/s0889-8529(01)00005-6.

Voss, U., 2004. Functions of sleep architecture and the concept of protective fields. Rev. Neurosci., 15, 33-46. https://doi.org/10.1515/revneuro.2004.15.1.33

Watson, C.J., Baghdoyan, H.A., Lydic, R., 2010. Neuropharmacology of Sleep and Wakefulness. Sleep Med. Clin. 5, 513-528. https://doi.org/10.1016/j.jsmc.2010.08.003.

Wehr, T.A., 1992. A brain-warming function for REM sleep. Neurosci. Biobehav. Rev., 16, 379-397. https://doi.org/10.1016/s0149-7634(05)80208-8.

Weitzman, E.D., Nogeire, C., Perlow, M., Fukushima, D., Sassin, J., McGregor, P., Hellman, L., 1974. Effects of a prolonged 3-hour sleep-wake cycle on sleep stages, plasma cortisol, growth hormone and body temperature in man. J. Clin. Endocrinol. Metab. 38, 1018-1030. https://doi.org/10.1210/jcem-38-6-1018.

Weitzman, E.D., Zimmerman, J.C., Czeisler, C.A., Ronda, J., 1983. Cortisol secretion is inhibited during sleep in normal man. J. Clin. Endocrinol. Metab. 56, 352-358. https://doi.org/10.1210/jcem-56-2-352.

Williams, L., 2025. 10 animals that can kill a lion. Discoverwildlife. Available at: https://www.discoverwildlife.com/animal-facts/mammals/animals-that-can-kill-a-lion (accessed 26 Aug, 2025).

Yamaguchi, H., Hopf, F.W., Li, S.B., de Lecea, L. (2018). In vivo cell type-specific CRISPR knockdown of dopamine beta hydroxylase reduces locus coeruleus evoked wakefulness. Nat. Commun. 9, 5211. https://doi.org/10.1038/s41467-018-07566-3.

Yuksel, C., Denis, D., Coleman, J., et al., 2025. Both slow wave and rapid eye movement sleep contribute to emotional memory consolidation. Commun. Biol. 8, 485. https://doi.org/10.1038/s42003-025-07868-5.

Yu, W., Zadbood, A., Chanales, A.J.H., Davachi, L., 2024. Repetition dynamically and rapidly increases cortical, but not hippocampal, offline reactivation. Proceedings of the National Academy of Sciences of the United States of America, 121, e2405929121. https://doi.org/10.1073/pnas.2405929121

Zhong, Z., Yan, F., Xie, C., 2024. Waking Up Brain with Electrical Stimulation to Boost Memory in Sleep: A Neuroscience Exploration. Neurosci. Bull. 40, 852-854. https://doi.org/10.1007/s12264-024-01200-7.
